# ADVANCE-ID bacterial and fungal infections review: antimicrobial resistance research updates

**DOI:** 10.1093/jacamr/dlag198

**Published:** 2026-09-10

**Authors:** Abi Manesh, Yin Mo, Jennifer Wang, David L Paterson

**Affiliations:** ADVANCE-ID, Saw Swee Hock School of Public Health, National University of Singapore, Singapore; Department of Infectious Diseases, Christian Medical College (CMC) Vellore, Vellore, India; ADVANCE-ID, Saw Swee Hock School of Public Health, National University of Singapore, Singapore; Saw Swee Hock School of Public Health, National University of Singapore and National University Health System, Singapore; Infectious Diseases Translational Research Programme, Yong Loo Lin School of Medicine, National University of Singapore, Singapore; Mahidol-Oxford Tropical Medicine Research Unit, Mahidol University, Bangkok, Thailand; ADVANCE-ID, Saw Swee Hock School of Public Health, National University of Singapore, Singapore; ADVANCE-ID, Saw Swee Hock School of Public Health, National University of Singapore, Singapore; Infectious Diseases Translational Research Programme, Yong Loo Lin School of Medicine, National University of Singapore, Singapore

## Introduction

In this series, we summarize landmark trials published in the field of antimicrobial resistance (AMR) in the last 2 months. Here, we discuss the practice-changing impact of the SNAP trial on MSSA bacteraemia; a novel diazabicyclooctane (DBO) β-lactamase-inhibitor, nacubactam, completing Phase 3 evaluation; and a pragmatic drug-resistant TB trial that has influenced WHO management guidelines for resistant TB.

## SNAP trial

Many treatment guidelines recommend an antistaphylococcal penicillin as the preferred option for the management of MSSA bacteraemia. However, some clinicians use cefazolin as a practical alternative, but concerns about the cefazolin inoculum effect remain. SNAP is an ongoing global, adaptive, Bayesian platform trial, and the currently published MSSA treatment domain compares cefazolin with an antistaphylococcal penicillin, cloxacillin or flucloxacillin, in appropriate doses.^1^ The primary outcome was 90 day, all-cause mortality. The prespecified criterion for non-inferiority was an OR of <1.20 for 90 day mortality, with at least 97.5% posterior probability.

The primary outcome occurred in 15.0% (97 of 654) in the cefazolin group and 17.0% (109 of 642) in the antistaphylococcal penicillin group (adjusted OR, 0.81; 95% credible interval, 0.59–1.12) with a 99.2% probability of non-inferiority, and 89.8% probability of superiority. Importantly, the risk of renal injury was higher in the antistaphylococcal penicillin group (20% versus 14%). This pattern is consistent with observations from other trials—the penicillin susceptible *Staphylococcus aureus* (PSSA) domain of SNAP (acute kidney injury rate = 22%), CloCeBa (17%) and CAMERA 2 (27% when combined with vancomycin versus 6% in vancomycin-alone group) trials.^2^ Antistaphylococcal penicillins have a higher propensity to cause nephrotoxicity through several mechanisms, including accumulation in proximal tubular cells and acute interstitial nephritis.^3^

Central to the cefazolin versus antistaphylococcal penicillin debate is the unresolved question of the cefazolin inoculum effect.^4^ The *blaZ* gene, located in the accessory genome of *S. aureus,* encodes a β-lactamase that hydrolyses benzyl penicillin, amoxicillin and ampicillin. Antistaphylococcal penicillins like (flu)cloxacillin are stable against this enzyme, due to their bulky side chain, preventing access to the active site. Importantly, the hydrolysing ability of this enzyme against cefazolin is variable and dependent on the allotype, with several allotypes described (types A, B, C and D). Allotype A confers more cefazolin hydrolysis than others. This hydrolysing activity directly correlates with the cefazolin inoculum effect, resulting in elevated cefazolin MICs in high-burden infections. A recently published trial that explored the same clinical question comparing cloxacillin and cefazolin, the CloCeBa trial, identified type A *blaZ* gene as being associated with poor outcomes in the subgroup analysis, though numbers were small (58 patients in both arms in the *blaZ*-positive population for the 90 day survival outcome).^5^ The proportion of MSSA isolates without the *blaZ* gene, resulting in penicillin-susceptible isolates (PSSA), is increasing globally. The SNAP platform approached this problem by removing PSSA isolates from the domain, so likely all the isolates included in the MSSA domain had a functional gene and hence varying degrees of cefazolin inoculum effect, depending on the allotype. The MSSA domain results then argue against a large-scale inoculum effect across the population, which is reassuring. The trial results reflect an average treatment effect across the population and do not exclude heterogeneous effects within specific BlaZ allotypes. Further sub-analyses with specific *blaZ* allele types and outcomes are awaited.

## BEAT TB

BEAT is a pragmatic, non-inferiority, randomized controlled trial (RCT) evaluating a 6 month, all-oral antitubercular regimen for rifampicin-resistant TB versus a 9 month standard of care.^6^ This trial builds on the evidence from TB PRACTECAL, NixTB and ZeNix trials but with several important caveats. Unlike others, this is a pragmatic, strategy-based trial evaluating a 6 month regimen with bedaquiline, linezolid, delamanid, and levofloxacin or clofazimine (or both) for 6 months against a 9 month, oral, standard-of-care regimen in South Africa. This is the first drug-resistant TB trial to include children (>6 years), pregnant women and breast-feeding mothers in the primary population, substantially expanding the universal applicability of the approach. Fluoroquinolone-resistant pre-XDR TB patients were also included and regimens were modified accordingly.

The primary efficacy outcome was cure and completion of treatment at end of therapy and 76 weeks, as per standard WHO definitions. The non-inferiority margin was 10%. The primary safety outcome was grade 3 or higher adverse drug reactions. The pragmatic design enabled recruitment of 403 of the 432 screened patients. The majority of the patients in the intervention (86.1%) and control arm (86%) had clinical success (adjusted risk difference, −0.2 percentage points; 95% CI, −6.9 to 6.5; *P* = 0.001 for non-inferiority). The safety and relapse data were also comparable in the groups.

The BEAT TB trial replaced pretomanid with delamanid in the intervention arm. Pretomanid has significant access issues in many areas and currently lacks safety data for children under 14 years, and pregnant and lactating women.^7^ Having a regimen that can be used in all groups of patients improves uptake in government programmes and overall adherence. BEAT TB along with other trials show how evidence develops from single arm studies to parallel arm trials and finally a pragmatic RCT, with inclusive populations, strategy over strict regimens and improving access.

## Integral 1 trial

Integral 1 is a global, double-blind RCT evaluating the efficacy and safety of two β-lactam/β-lactamase inhibitor combinations, cefepime/nacubactam and aztreonam/nacubactam, against imipenem/cilastatin in a ratio of 2:1:1 among patients with carbapenem-susceptible Enterobacterales causing complicated urinary tract infection and pyelonephritis.^8^ The trial was funded by Meiji Seika Pharma and Japan Agency for Medical Research and Development. The primary outcome was a composite of clinical and microbiological success in the microbiological intention-to-treat population. The trial was powered for non-inferiority of both agents at a margin of 15% and for hierarchical testing for superiority with cefepime/nacubactam.

The primary outcome difference between the trial arms and the carbapenem comparator was stark: 21.3% (95% CI, 10.9 to 32.0) for cefepime/nacubactam (non-inferior and superior), and 11.4% (95% CI, −1.2 to 23.7) for aztreonam/nacubactam (non-inferior). The safety events were comparable across the trial arms. Subgroups showed numerically superior performance of the trial interventions in patients with ESBL-producing organisms and microbiological clearance.

Whether the intrinsic antibacterial action of nacubactam via PBP2 contributed to the differences in ESBL and microbiological clearance is unknown. But these combinations are particularly valuable in regions where PBP3 inserts increase aztreonam/avibactam and cefiderocol MICs, especially in combination with acquired CMY-42 and CTX-M-15 enzymes.^9^ The clinical efficacy of these agents against carbapenem-resistant Enterobacterales is being evaluated in the yet to be published Integral 2 trial (NCT05905055).

## Supplementary Material

dlag198_Supplementary_Data
