# Supplementary material for: ADVANCE-ID bacterial and fungal infections review: antimicrobial resistance research updates
Source: JAC Antimicrob Resist. 2026 Sep 10;8(5):dlag198. doi: 10.1093/jacamr/dlag198 (PMC13557829; doi:10.1093/jacamr/dlag198)
Supplement: Supplementary file 1 [file dlag198_supplementary_data.docx]

**Randomised Controlled Trials^[1]^**

| **Citation of Articles** | **PICO** | **Main Results** | **Risk of Bias** |
| --- | --- | --- | --- |
| [Banerjee R, Komarow L, Li Y, Mau D, Dodd A, Geres H, et al. Fast antimicrobial susceptibility testing for gram-negative bacteraemia: the FAST randomized clinical trial. JAMA. 2026 Apr 18; doi:10.1001/jama.2026.5487](https://ad-id.co/3QHKYlx)   - **Editorial Commentary:**   [Srinivasan A. A multinational trial of rapid antimicrobial susceptibility testing: is FASTer better? JAMA. 2026 Apr 18; doi:10.1001/jama.2026.5504](https://ad-id.co/4uh38sG) | **P:** 850 hospitalized patients (adults and children) with gram-negative bloodstream infections (median age 72 years, 43% female) from 7 centers across 4 countries with high antimicrobial resistance  **I:** Rapid phenotypic antimicrobial susceptibility testing (AST) directly from positive blood cultures plus standard testing  **C:** Standard antimicrobial susceptibility testing alone  **O:** Desirability of outcome ranking (DOOR) at day 30 (alive without deleterious events, alive with deleterious events, death); secondary outcomes included 30-day mortality, time to effective therapy, and time to antibiotic escalation/de-escalation | Rapid AST did not improve the primary outcome, with a 48.8% probability of more desirable DOOR outcomes compared with standard testing (95% CI 45.3–52.4%), failing to meet superiority criteria. There was no difference in median time to effective antibiotic therapy in the overall population, but time to antibiotic escalation or de-escalation was faster with rapid testing by 14 hours (95% CI 6–22). No significant differences were observed in mortality or other secondary outcomes. In the subgroup with carbapenem-resistant infections, rapid AST reduced time to effective therapy (9.5 vs 28 hours, difference −18 hours, 95% CI −42 to 6), although this was not statistically definitive. | Moderate risk: The open-label design introduces potential performance and detection bias, although objective outcomes such as mortality reduce this concern. Randomization across multiple international centers improves generalizability, but heterogeneity in stewardship practices may influence outcomes. High inclusion in primary analysis (850/899) limits attrition bias. The DOOR endpoint, while comprehensive, may introduce subjectivity in outcome prioritization. |
| [Llor C, Monfà R, Garcia-Sangenís A, Leiva A, Marín-Cañada J, Sánchez-Calavera MA, et al. Clinical and bacteriological effectiveness of three different short-course antibiotic regimens and single-dose fosfomycin for uncomplicated lower urinary tract infections in women (SCOUT): a pragmatic, multicentre, open-label, randomised clinical trial. Lancet. 2026 Apr 25;407(10539):1603-1613. doi:10.1016/S0140-6736(25)02171-3](https://ad-id.co/4uio9TW)   - **Editorial Commentary:**   [Rodríguez-Baño J, Retamar-Gentil P. Treatment of uncomplicated lower urinary tract infections in women. Lancet. 2026 Apr 25;407(10539):1574-1575. doi:10.1016/S0140-6736(25)02269-X](https://ad-id.co/48A2EFP) | **P:** 768 women (≥18 years) with uncomplicated UTI symptoms and positive urine dipstick in Spanish primary care (720 included in primary analysis; median age 48 years)  **I:** Single-dose fosfomycin (3 g) or two-dose fosfomycin (3 g ×2)  **C:** Nitrofurantoin (100 mg three times daily for 5 days) or pivmecillinam (400 mg three times daily for 3 days)  **O:** Clinical resolution at day 7 (complete symptom disappearance); adverse events | Nitrofurantoin achieved the highest clinical resolution (74%) compared with single-dose fosfomycin (59%), with a significant difference of 15.5 percentage points (95% CI 5.9–25.1; p=0.0168). Pivmecillinam (70%) and two-dose fosfomycin (67%) also showed higher resolution rates than single-dose fosfomycin, but differences were not statistically significant. Adverse events were similar across groups (19.9%–26.8%), mostly mild and gastrointestinal. Four serious adverse events occurred, with only one treatment-related (pyelonephritis in the pivmecillinam group). | Moderate risk: The open-label design introduces potential performance and reporting bias, particularly for subjective symptom resolution. Pragmatic multicentre design enhances generalizability, but lack of blinding may influence patient-reported outcomes. Attrition from 768 randomized to 720 analyzed may introduce some attrition bias. Comparable group sizes and randomization support internal validity. |
| [Narang H, Talukdar D, Kumar B, Mathur P, Ningombam A, Singh M, et al. Fecal microbiota transplant and multidrug-resistant organism decolonization in gastrointestinal disease: a randomized clinical trial. JAMA Intern Med. 2026 Apr 20; doi:10.1001/jamainternmed.2026.0655](https://ad-id.co/4ujF6gS) | **P:** 114 patients with gastrointestinal diseases and persistent multidrug-resistant organism (MDRO) colonization (mean age 40.6 years, 70.2% male) in a tertiary care center in India  **I:** Single-session fecal microbiota transplant (FMT) via colonoscopy  **C:** Sham intervention (sigmoidoscopy with saline injection)  **O:** MDRO decolonization and reduction in antimicrobial resistance (AMR) genes at 4 weeks; secondary outcomes included microbiome, virome, mycobiome changes, MDRO infections, and adverse events | FMT did not improve MDRO decolonization compared with sham (31.0% vs 30.4%, absolute difference 0.6%, 95% CI −16.2% to 17.6%; P=0.94) and showed no significant reduction in AMR gene abundance (median 2.5 vs 2.0 genes; P=0.68). Adverse events were comparable between groups. However, FMT increased gut bacterial diversity and enriched short-chain fatty acid–producing bacteria, changes not observed in the sham group. No significant differences were seen in virome diversity, and only modest, transient changes occurred in the mycobiome. | Low–moderate risk: Double-blind, sham-controlled design minimizes performance and detection bias. Small sample size limits statistical power to detect modest effects. Minimal loss to follow-up reduces attrition bias. Single-center setting may limit generalizability. Objective microbiological outcomes strengthen internal validity, though short follow-up (4 weeks) may not capture longer-term effects. |
| [Major Extremity Trauma Research Consortium (METRC). Intrawound tobramycin plus vancomycin to prevent surgical site infection in tibial fractures: the TOBRA randomized clinical trial. JAMA. 2026 Apr 15; doi:10.1001/jama.2026.4023](https://ad-id.co/4uhUxpN) | **P:** 1528 adults with operatively treated periarticular tibial fractures at elevated risk of infection (mean age 47 years, 39.5% female) across 39 US trauma centers  **I:** Intrawound tobramycin (1.2 g) plus vancomycin (1.0 g) powder at definitive fixation  **C:** Intrawound vancomycin (1.0 g) powder alone  **O:** Deep surgical site infection requiring surgical management within 182 days; secondary outcomes included microbiological subtypes of infection and cellulitis | The addition of tobramycin to vancomycin did not reduce deep surgical site infections compared with vancomycin alone (7.4% vs 6.6%; hazard ratio 1.11, 95% Bayesian credible interval 0.75–1.66; posterior probability of superiority 29.7%). No superiority was observed for any secondary outcomes, including infections by gram-negative or gram-positive organisms or polymicrobial infections. Overall infection rates were similar between groups, indicating no added benefit of combination therapy. | Moderate risk: Open-label design may introduce performance bias, although assessor masking reduces detection bias. Large sample size and multicenter design strengthen internal validity and generalizability. Objective primary outcome reduces measurement bias. Some attrition from 1660 randomized to 1528 analyzed may introduce minimal attrition bias. Bayesian analysis approach may affect interpretability but does not inherently increase bias. |
| [Jain A, Agarwal N, Anand R, Padhi P, Jindal A. 7-days versus 14-days antibiotic therapy in uncomplicated culture proven neonatal sepsis: a randomized control assessor-blinded trial. Eur J Pediatr. 2026 Apr 15;185:268. doi:10.1007/s00431-026-06921-7](https://ad-id.co/4tTiofy) | **P:** 140 neonates (≥1000 g) with culture-positive uncomplicated sepsis who improved after initial therapy in a tertiary NICU in Central India (70 per group)  **I:** 7-day antibiotic regimen  **C:** 14-day antibiotic regimen  **O:** Relapse of sepsis; secondary outcomes included hospital stay and need for respiratory support | The 7-day antibiotic regimen was non-inferior to the 14-day regimen, with no definitive relapses or deaths observed in either group and only a low incidence of probable relapse. The 7-day group had significantly shorter hospital stays and required less respiratory support (both p<0.05), suggesting potential clinical and resource benefits without compromising safety. | Moderate risk: Assessor-blinded design reduces detection bias, but lack of full blinding may introduce performance bias. Small sample size (n=140) limits statistical power and precision of non-inferiority conclusions. Selection of only clinically improving neonates may limit generalizability. Short follow-up period (35 days) may miss late relapses. Randomization after initial response could introduce selection bias. |
| [Hagman K, Hedenstierna M, Andersson Norlén E, Biasoletto K, Eklund Josephson M, Fraenkel CJ, et al. Adjunctive betamethasone treatment of hypoxaemic adults hospitalised with *Mycoplasma pneumoniae* community-acquired pneumonia: an open-label, multicentre, randomised, controlled trial. Lancet Reg Health Eur. 2026 Apr 19; doi:10.1016/j.lanepe.2026.101610](https://ad-id.co/4tLnkmS)   - **Editorial Commentary:**   [Teja B, Marshall JC. Corticosteroids for Mycoplasma pneumoniae community-acquired pneumonia: promising evidence from a randomised controlled trial. Lancet Reg Health Eur. 2026 Apr 19; doi:10.1016/j.lanepe.2026.101677](https://ad-id.co/48xomdG) | **P:** 70 adults hospitalised with Mycoplasma pneumoniae community-acquired pneumonia and hypoxaemia (SpO₂ <93%, RR >20) in 8 Swedish hospitals (median age 42 years, 57% male)  **I:** Adjunctive oral betamethasone (3 mg days 1–2, 2 mg days 3–5) plus standardised doxycycline  **C:** Standard care without corticosteroids plus standardised doxycycline  **O:** Time to resolution of hypoxaemia; safety outcomes | Adjunctive betamethasone significantly shortened time to resolution of hypoxaemia compared with standard care (HR 1.82, 95% CI 1.10–3.02; p=0.020), with median time 2.3 days (95% CI 1.8–2.7) vs 3.6 days (95% CI 1.9–5.3). Nearly all participants achieved resolution, and adverse events were similar between groups. Severe adverse events were rare and none were attributed to betamethasone, indicating good tolerability. | Moderate risk: Open-label design introduces potential performance and detection bias, especially for clinically assessed outcomes. Small sample size (n=70) limits statistical power and precision. Multicentre design supports generalizability, but exclusion of patients with asthma and diabetes may limit applicability. Objective outcome (oxygenation) partially mitigates bias. |
| [Heath P, Zuma-Gwala N, Helmig RB, Horne E, Kjærbye-Thygesen A, Wiinblad Crusell MK, et al. Immunogenicity and safety of a group B Streptococcus vaccine (GBS-AlpN) in pregnant women and their infants: a phase 2, multicentre, observer-blind, randomised, placebo-controlled study. Lancet Infect Dis. 2026 May;26(5):486-496. doi:10.1016/S1473-3099(25)00659-0](https://ad-id.co/4tOpAtI) | **P:** 272 pregnant women (21–24 weeks’ gestation, singleton pregnancies) across 10 centres in South Africa, Denmark, and the UK (269 received ≥1 dose)  **I:** GBS-AlpN maternal vaccination (one or two doses at varying schedules during pregnancy)  **C:** Placebo (saline) injections only  **O:** Infant cord blood (or neonatal) IgG concentrations against GBS AlpN proteins; proportion achieving predefined antibody thresholds; safety outcomes in mothers and infants | GBS-AlpN vaccination was immunogenic, with the highest infant IgG concentrations observed in the two-dose groups, particularly group 1. A high proportion of infants in vaccine groups achieved IgG >1.0 μg/mL (up to 94% in group 1), compared with none in the placebo group. One-dose vaccination also increased antibody levels, though lower than two-dose regimens, but still at least 21-fold higher than placebo. Adverse events were common across all groups (87–97%) but mostly mild to moderate. Nine infant deaths occurred, none related to vaccination, supporting an acceptable safety profile. | Low–moderate risk: Observer-blind, placebo-controlled design minimizes detection and performance bias. Randomization and multicentre setting enhance internal validity and generalizability. Moderate sample size limits precision for subgroup comparisons. Immunogenicity (surrogate endpoint) rather than clinical infection outcomes limits direct clinical applicability. High rates of reported adverse events may reflect active monitoring rather than true differences. |
| [Leitner DR, Walsh SR, Suzuki M, Desjardins M, Hannaford A, Sherman AC, et al. Safety and immunogenicity of PanChol, a single-dose live-attenuated oral cholera vaccine: results from a phase 1a, double-blind, randomised, placebo-controlled trial. Lancet Infect Dis. 2026 May;26(5):497-509. doi:10.1016/S1473-3099(25)00682-6](https://ad-id.co/4tI0AUV) | **P:** 57 healthy adults (18–55 years, median age 30.6 years, 53% female) without prior cholera infection or vaccination in a first-in-human phase 1 trial (36 in randomized module)  **I:** Single-dose oral live-attenuated PanChol vaccine (various doses, including 10⁷–10⁸ CFU in randomized phase)  **C:** Placebo (oral matching diluent)  **O:** Safety (adverse events) and immunogenicity (≥4-fold rise in vibriocidal antibody titres to Inaba and Ogawa at day 15); secondary outcome: stool shedding | PanChol was well tolerated, with mostly mild and transient adverse events; diarrhoea was the most common (39% in vaccine vs 38% in placebo). No serious safety concerns were identified. In the randomized phase, unsolicited adverse events were similar between groups and mostly mild. Immunogenicity was strong, with 100% of vaccine recipients receiving ≥10⁵ CFU achieving seroconversion against both serotypes. Stool shedding occurred in most vaccine recipients at ≥10⁵ CFU (96%), but not in placebo recipients. These results demonstrate robust immune response across a wide dose range with acceptable safety. | Moderate risk: Early-phase (phase 1) trial with small sample size limits generalizability and precision. Mixed design (open-label dose-escalation and randomized double-blind phase) introduces potential bias in non-randomized components. Randomized, placebo-controlled module strengthens internal validity. Use of surrogate immunogenicity endpoints rather than clinical protection limits applicability. Short follow-up duration may not capture longer-term safety or durability of response. |
| [Miller LG, Kolar S, Sanders J, Williamson J, Allyn PR, Baang J, et al. A phase 2a randomized, double-blind, controlled trial of the efficacy and safety of an intravenous (IV) bacteriophage cocktail (AP-SA02) vs placebo in combination with best available antibiotic therapy (BAT) in patients with complicated *Staphylococcus aureus* bacteraemia. Open Forum Infect Dis. 2026 Jan 11;13(Suppl 1):ofaf695.022. doi:10.1093/ofid/ofaf695.022](https://ad-id.co/495dfc1) | **P:** 42 patients with complicated Staphylococcus aureus bacteraemia (SAB) (including MRSA ~39–44%) across 17 sites (95% US)  **I:** Intravenous bacteriophage cocktail AP-SA02 every 6 hours for 5 days plus best available therapy (BAT)  **C:** Placebo plus best available therapy (BAT)  **O:** Clinical response at day 12 (test of cure), post-therapy and 4-week follow-up (relapse/non-response); safety outcomes | AP-SA02 improved clinical response at day 12 compared with placebo (88% vs 58% by investigators, p=0.047; 83% vs 58% by adjudication). At post-therapy and 4-week follow-up, relapse/non-response was 0% in the AP-SA02 group vs 23–25% in the placebo group (p<0.025). Trends favored AP-SA02 for faster normalization of inflammatory markers, shorter time to blood culture clearance, and reduced ICU and hospital stay. Adverse events were infrequent (6% vs 0%) and no major safety concerns were identified. | The results are only available as an abstract so caution is urged!  Moderate risk: Small sample size (n=42) limits statistical power and precision. Randomized, double-blind design with adjudicated outcomes reduces bias. Imbalance in group sizes (2:1 randomization) may affect comparisons. Use of adjunctive BAT introduces heterogeneity in background therapy. Early-phase (phase 2a) trial limits generalizability and requires confirmation in larger studies. |
| [Takahashi S, Tateda K, Yanagihara K, Huang H, Doi Y, Yasuda M, et al. Efficacy and safety of cefepime–nacubactam and aztreonam–nacubactam compared with imipenem–cilastatin for complicated urinary tract infection or acute uncomplicated pyelonephritis (Integral-1): a double-blind, randomised phase 3 trial. Lancet. 2026 May 16;407(10542):1929-1940. doi:10.1016/S0140-6736(26)00596-9](https://ad-id.co/4dkTcYw) | **P:** 614 adults with complicated urinary tract infection (cUTI) or acute uncomplicated pyelonephritis recruited from 79 centres across Europe and Asia; 431 patients with baseline qualifying pathogens included in the microbiological modified intention-to-treat analysis.  **I:** Intravenous cefepime 2 g plus nacubactam 1 g every 8 h or aztreonam 2 g plus nacubactam 1 g every 8 h for 5–14 days.  **C:** Intravenous imipenem 1 g plus cilastatin 1 g every 8 h for 5–14 days.  **O:** Composite clinical and microbiological success at test-of-cure; treatment-emergent adverse events. | Composite clinical and microbiological success at test-of-cure was achieved in 82% (176/214) of the cefepime–nacubactam group, 72% (81/112) of the aztreonam–nacubactam group, and 61% (64/105) of the imipenem–cilastatin group. Cefepime–nacubactam demonstrated both non-inferiority and superiority versus imipenem–cilastatin, with an absolute difference of 21.3% (95% CI 10.9–32.0). Aztreonam–nacubactam achieved non-inferiority with an absolute difference of 11.4% (95% CI –1.2 to 23.7). Treatment-emergent adverse events occurred in 33% of cefepime–nacubactam recipients, 30% of aztreonam–nacubactam recipients, and 43% of imipenem–cilastatin recipients; no treatment-related deaths were reported. | Low-to-moderate risk: The randomised, double-blind, multicentre phase 3 design with stratified randomisation strengthens internal validity and reduces selection and performance bias. Objective microbiological endpoints support reliability of outcome assessment. However, the primary efficacy analysis excluded a substantial proportion of randomised participants who lacked qualifying pathogens, which may limit generalisability. Unequal randomisation (2:1:1) and smaller sample size in the aztreonam–nacubactam arm may also reduce precision for comparative estimates. |
| [Fernández Moreno A, López de Ugarriza P, González Huerta AJ, Boluda Pascual B, Cáceres S, Solán Blanco L, et al. Fosfomycin versus ciprofloxacin for febrile neutropenia prophylaxis in high-risk haematological patients (FOVOCIP): a phase 3, open-label, multicentre, randomised, non-inferiority trial. Lancet Reg Health Eur. 2026 Jul;66:101698. doi:10.1016/j.lanepe.2026.101698](https://ad-id.co/3RdjDIa) | **P:** 166 high-risk haematological patients (50% female) with acute leukaemia or undergoing haematopoietic stem cell transplantation at 11 centres in Spain.  **I:** Oral fosfomycin prophylaxis during neutropenia.  **C:** Oral ciprofloxacin prophylaxis during neutropenia.  **O:** Febrile neutropenia; Gram-negative bacteraemia; multidrug-resistant Gram-negative colonisation; safety outcomes. | In the intention-to-treat population, febrile neutropenia occurred in 74.7% (62/83) of patients receiving fosfomycin and 73.5% (61/83) receiving ciprofloxacin, corresponding to success rates of 25.3% versus 26.5%, respectively (risk difference −1.2%, 95% CI −14.5 to 12.1); non-inferiority criteria were not met. In the per-protocol analysis, success rates were 17.8% versus 23.8% (risk difference −5.9%, 95% CI −18.8 to 6.9), again failing to demonstrate non-inferiority. Gram-negative bacteria accounted for 90% of bacteraemias in the fosfomycin arm compared with 10% in the ciprofloxacin arm. Multidrug-resistant Gram-negative colonisation occurred less frequently with fosfomycin than ciprofloxacin (3% vs 11%, P=0.04). Other safety outcomes were similar between groups. | Moderate risk: The randomised multicentre phase 3 design strengthens internal validity, but the open-label nature introduces potential performance and detection bias, especially for clinically assessed outcomes such as febrile neutropenia. The relatively small sample size limits precision and may reduce power for secondary outcomes. Use of both ITT and per-protocol analyses supports robustness, although failure to meet non-inferiority margins and differential microbiological outcomes may reflect variability in patient management and infection risk across centres. |
| [Cossa M, Ndege R, Meggi B, Mangu C, Leukes V, Sabi I, et al. Expanding Xpert MTB/RIF Ultra and lateral flow urine lipoarabinomannan testing for diagnosis of tuberculosis among adults living with HIV admitted to hospitals in Tanzania and Mozambique (EXULTANT): a randomised controlled trial. Lancet Infect Dis. 2026 May 6; doi:10.1016/S1473-3099(26)00133-7](https://ad-id.co/4eIgSbE)   - **Editorial Commentary:**   [Shapiro AE. Non-sputum testing: an incomplete bridge across the tuberculosis diagnostic gap. Lancet Infect Dis. 2026 May 6; doi:10.1016/S1473-3099(26)00184-2](https://ad-id.co/4dsg7Bg) | **P:** 1172 hospitalised adults living with HIV in Tanzania and Mozambique without existing tuberculosis diagnosis or recent tuberculosis treatment; 61% female, median CD4 count 232 cells/μL, 75% on antiretroviral therapy.  **I:** Expanded tuberculosis screening using Xpert MTB/RIF Ultra testing on sputum, stool, and urine plus urine lateral flow lipoarabinomannan (LF-LAM) testing irrespective of symptoms.  **C:** Standard-of-care symptom-based WHO-recommended sputum Xpert MTB/RIF Ultra and LF-LAM testing.  **O:** Microbiologically confirmed tuberculosis starting treatment within 72 h; 8-week all-cause mortality; time to tuberculosis treatment initiation. | Microbiologically confirmed tuberculosis with treatment initiation within 72 h occurred in 16.0% (93/582) of the intervention group and 15.3% (90/590) of the control group (difference 0.7%, 95% CI −3.4 to 4.8; p=0.73), showing no significant improvement with expanded screening. Eight-week all-cause mortality was 25.8% in the intervention group versus 28.8% in the control group (HR 0.86, 95% CI 0.69–1.07; p=0.18). Median time to tuberculosis treatment initiation was similar between groups at 0.98 versus 0.92 days (HR 1.05, 95% CI 0.82–1.33; p=0.72). Overall, expanded non-sputum-based screening did not improve tuberculosis treatment initiation or survival outcomes compared with standard symptom-based testing. | Low-to-moderate risk: The pragmatic, individually randomised multicentre design across 11 hospitals strengthens external validity and reduces selection bias. Objective microbiological and mortality outcomes reduce detection bias. However, the pragmatic implementation and incomplete sputum sample provision in the control arm may have introduced variability in diagnostic testing and intervention delivery. Lack of blinding could potentially influence clinical management decisions, although primary outcomes were largely objective. High mortality and heterogeneous disease severity may also limit the ability to detect modest intervention effects. |
| [Thorn N, Danos Z, Andrews NJ, Sutton N, Cathie K, Owens D, et al. Immunogenicity, reactogenicity, and safety of a reduced-interval 4CMenB primary immunisation schedule in UK infants: a multicentre, open-label, assessor-blinded, phase 4 randomised controlled trial. Lancet Infect Dis. 2026 May 6; doi:10.1016/S1473-3099(26)00119-2](https://ad-id.co/4dkXLSA) | **P:** 221 full-term, immunocompetent infants in 6 hospitals in England (50% male), enrolled in a phase 4 randomised controlled trial evaluating infant MenB vaccination schedules.  **I:** Reduced-interval 4CMenB schedule (8 and 12 weeks) with PCV13 at 16 weeks, plus booster doses at 1 year.  **C:** Standard schedule 4CMenB (8 and 16 weeks) with PCV13 at 12 weeks, plus booster doses at 1 year.  **O:** Immunogenicity (hSBA GMTs, protective titres ≥4), pneumococcal antibody responses, reactogenicity, and safety outcomes. | Protective hSBA titres 4 weeks post-primary vaccination were high in both groups and not significantly different for all MenB strains (e.g. fHbp 93% vs 97%; PorA 64% vs 75%). However, post-primary hSBA GMTs were lower in the reduced-interval group (GMRs 0.61–0.73 across strains), while pre-booster titres were similar between groups. Post-booster responses were generally comparable or higher in the reduced-interval group, significantly so for PorA (GMR 1.55, 95% CI 1.09–2.22). Pneumococcal responses were broadly similar post-booster across groups. Reactogenicity was significantly lower in the reduced-interval group, with reduced irritability (RD −0.10, p=0.0091) and crying (RD −0.19, p=0.0012). | Moderate risk: Randomised multicentre design with assessor blinding for immunological outcomes strengthens internal validity. However, open-label nature introduces potential performance and reporting bias for reactogenicity outcomes collected via parental diaries. Small sample size limits precision and generalisability. Timing deviations in blood sampling and inclusion of samples outside ideal windows may introduce measurement variability, although masked laboratory analysis mitigates detection bias. |
| [De Cock PA, Colman R, Smits A, Piersigilli F, Renard M, Van Damme A, et al. Bedside model-informed precision dosing of vancomycin in severely ill neonates and children in Belgium (the BENEFICIAL trial): a multicentre, randomised controlled trial. Lancet Child Adolesc Health. 2026 Jun;10(6):406-17. doi:10.1016/S2352-4642(25)00385-2](https://ad-id.co/4nWJ10Z) | **P:** 314 critically ill paediatric patients (<18 years) in intensive care or haemato-oncology units receiving intravenous vancomycin for suspected or confirmed Gram-positive infection  **I:** Model-informed precision dosing (MIPD) of vancomycin using Bayesian software with early therapeutic drug monitoring and AUC-guided dosing adjustments  **C:** Standard-of-care therapeutic drug monitoring (TDM) of vancomycin  **O:** Achievement of 24-h AUC-to-MIC ratio target of 400–600 mg·h/L at 24–48 h; acute kidney injury or all-cause mortality; serious adverse events | MIPD significantly improved target AUC-to-MIC attainment at 24–48 h compared with standard TDM (71.8% vs 53.9%; absolute difference 18.9%, 95% CI 1.7–34.7). Acute kidney injury or all-cause mortality was numerically lower in the MIPD group but not statistically significant (12.4% vs 16.9%; absolute difference –4.5%, 95% CI –11.6 to 3.5). Serious adverse events occurred at similar rates in both groups (5% each). Overall, AUC-guided MIPD improved pharmacokinetic target attainment with low risk of harm. | Moderate risk: The open-label design for treating clinicians and pharmacists could introduce performance bias, although randomisation, allocation concealment, and masking of patients, families, and biostatisticians reduce selection and detection bias. Objective pharmacokinetic outcomes strengthen internal validity. Exclusion of patients without deferred consent completion and incomplete availability of AUC data may introduce some attrition bias, though overall follow-up and safety reporting were robust. |
| [Vissing NH, Skajaa T, Grosen D, Albertsen BK, Als-Nielsen B, Hasle H, et al. Early discontinuation of empirical antibiotics versus extended treatment in children with cancer and high-risk febrile neutropenia in Denmark: an open-label, randomised controlled trial. Lancet Child Adolesc Health. 2026 Jun;10(6):418-28. doi:10.1016/S2352-4642(26)00039-8](https://ad-id.co/4dRY6fM) | **P:** 88 febrile neutropenia episodes in 70 children with cancer aged 0–17 years receiving intensive chemotherapy and experiencing high-risk febrile neutropenia without microbiologically documented infection  **I:** Early discontinuation of intravenous antibiotics after 48 h of defervescence and clinical stability regardless of neutrophil recovery  **C:** Continuation of intravenous antibiotics until neutrophil recovery or a maximum of 10 days  **O:** Duration of antibiotic exposure within 28 days; infection-related serious adverse events including bacteraemia, focal infection, sepsis, and death | Early discontinuation of antibiotics significantly reduced antibiotic exposure compared with standard continuation therapy, with a mean antibiotic duration of 8.1 days versus 13.5 days, respectively (estimated difference –4.0 days, 95% CI –6.0 to –1.9; p=0.0002). Serious adverse events occurred at similar rates between groups (22% vs 21%; risk ratio 1.06, 95% CI 0.46–2.47), and no deaths occurred. Overall, early discontinuation reduced unnecessary antibiotic use without evidence of compromised short-term safety. | Moderate risk: The open-label design could introduce performance bias, although allocation concealment and computer-generated randomisation strengthen internal validity. The small sample size and low event rates limit statistical power to detect rare safety outcomes. Use of febrile episodes rather than unique patients may introduce clustering effects, potentially affecting independence of observations and generalisability. |
| [Hay AD, Abbs S, Ridd M, Granier S, Lane JA, Muir P, et al. Rapid respiratory microbiological point-of-care testing and antibiotic use in primary care: a randomized clinical trial. JAMA Intern Med. 2026 May 18; doi:10.1001/jamainternmed.2026.1426](https://ad-id.co/4nYQa19)   - **Editorial Commentary:**   [Linder JA, Szymczak JE. Acute respiratory infections in primary care—precision microbiology fails to bring order to diagnostic disarray. JAMA Intern Med. 2026 May 18; doi:10.1001/jamainternmed.2026.1440](https://ad-id.co/4dEaVeZ) | **P:** 552 children and adults aged ≥12 months presenting to primary care with acute respiratory tract infection (≤21 days) where antibiotics were considered potentially necessary  **I:** Rapid multiplex microbiological point-of-care testing (RM-POCT) detecting 19 respiratory viruses and 4 atypical bacteria with results available in approximately 45 minutes  **C:** Usual care without RM-POCT  **O:** Same-day antibiotic prescribing; patient-reported symptom severity on days 2–4 | RM-POCT did not reduce same-day antibiotic prescribing compared with usual care, with antibiotics prescribed to 45% of participants in both groups (OR 1.00, 95% CI 0.71–1.41; p>0.99). There was no significant difference in symptom severity on days 2–4 between groups (difference in means 0.09, 95% CI –0.10 to 0.27; p=0.36). Prespecified subgroup analyses showed reduced antibiotic prescribing among patients with a detected viral pathogen (OR 0.35, 95% CI 0.20–0.63; interaction p<0.001) and those with chronic lung disease (OR 0.55, 95% CI 0.28–1.09; interaction p=0.046). Overall, RM-POCT did not improve overall antibiotic prescribing but appeared beneficial in selected subgroups without worsening clinical outcomes. | Moderate risk: The open-label nature for clinicians and patients could introduce performance bias, although outcome assessors and statisticians were masked to allocation. The primary outcome was objective and available for all participants, minimizing attrition bias. Safety outcome completion rates were moderate (74–78%), which may introduce some response bias. Subgroup findings should be interpreted cautiously as the study was not primarily powered for these analyses. |
| [Denninghoff KR, Casper TC, Zorc JJ, Ruddy RM, Satola S, Wendt WJ, et al. Azithromycin for preschoolers with wheezing in the emergency department. N Engl J Med. 2026 May 18; doi:10.1056/NEJMoa2516505](https://ad-id.co/4dQqMG5) | **P:** 840 preschool children aged 18–59 months presenting to emergency departments with moderate-to-severe wheezing episodes; 521 were nasopharyngeal pathogen-positive for *Streptococcus pneumoniae*, *Moraxella catarrhalis*, or *Haemophilus influenzae*  **I:** Azithromycin 12 mg/kg once daily for 5 days  **C:** Matching placebo for 5 days  **O:** Severity of wheezing symptoms measured by Asthma Flare-up Diary for Young Children (ADYC) over 5 days; secondary outcomes included ED/hospital length of stay, return visits, bacterial clearance, and antimicrobial resistance | Azithromycin did not reduce wheezing severity compared with placebo in either pathogen-positive or -negative cohorts. ADYC scores were similar between azithromycin and placebo groups in the positive cohort (median 9.59 vs 9.72; P=0.70) and negative cohort (9.30 vs 9.10; P=0.69). Despite significantly higher bacterial clearance in the azithromycin group (58.7% vs 11.4% in placebo among positives), this did not translate into improved clinical outcomes. Secondary outcomes, including healthcare utilization, resistance development, and adverse events, were also similar. | Low–moderate risk: Randomised, placebo-controlled, multicentre design with clear allocation to azithromycin vs placebo reduces selection and performance bias. Outcome assessment used standardized diary scoring, limiting detection bias. Early stopping for futility may reduce power for detecting smaller effects. Stratified analysis by bacterial status strengthens internal validity but increases risk of multiple subgroup comparisons. Overall attrition and safety reporting appear adequate. |
| [Portero-Prados FJ, Pabón-Carrasco M, Ponce-Blandón JA. Effectiveness of chlorhexidine gluconate–impregnated dressings in preventing central line–associated bloodstream infection in a paediatric intensive care unit: a randomised controlled trial. Intensive Crit Care Nurs. 2026 May 7; doi:10.1016/j.iccn.2026.104441](https://ad-id.co/4fk6N57) | **P:** 250 paediatric patients admitted to a tertiary paediatric intensive care unit with central venous catheters (125 per group)  **I:** Chlorhexidine gluconate (CHG)-impregnated central line dressings  **C:** Conventional transparent dressings  **O:** Central line–associated bloodstream infection (CLABSI) incidence; skin reactions; catheter-related risk factors including dwell time and vaccination status | CHG-impregnated dressings significantly reduced CLABSI incidence compared with conventional dressings (2.4% vs 18.4%; p<0.001). In multivariable analysis, CHG dressings remained independently protective against CLABSI (OR 0.15, 95% CI 0.04–0.58). Longer catheter dwell time increased infection risk (OR 1.13 per day, 95% CI 1.04–1.22), and incomplete vaccination status was associated with higher CLABSI risk (OR 8.68, 95% CI 2.48–30.47). Mild skin reactions were more frequent in the CHG group (16.8% vs 11.2%), particularly in younger infants, but did not require catheter removal. Overall, CHG dressings reduced infection risk with acceptable safety. | Moderate risk: Single-blind design may introduce performance bias, as clinicians were likely aware of dressing type. Randomisation and multivariable adjustment strengthen internal validity, but residual confounding cannot be excluded. Single-centre PICU setting limits generalisability. Outcome definition (CDC/NHSN CLABSI criteria) is standardized, reducing detection bias. Slight imbalance in skin reaction reporting may reflect partial unblinding. |
| [Faust K, Strecker F, Haug C, Felderhoff-Müser U, Stein A, Jensen R, et al. Extended barrier precautions vs hand hygiene alone and neonatal sepsis in intensive care patients: the BALTIC cluster-randomized clinical trial. JAMA Netw Open. 2026 May;9(5):e2612759. doi:10.1001/jamanetworkopen.2026.12759](https://ad-id.co/4ofzWkp) | **P:** 9731 neonates requiring intensive care across 12 tertiary neonatal intensive care units in Germany (cluster-randomized crossover design across 24 clusters)  **I:** Standard hand hygiene disinfection alone for infants colonized with third-generation cephalosporin–resistant gram-negative bacteria (3GCR-GNB)  **C:** Standard hand hygiene plus extended barrier precautions (gloves and gowns) for routine care  **O:** Health care–associated gram-negative bloodstream infections (primary outcome); transmission of 3GCR-GNB; overall infection rates | Standard hand hygiene was noninferior to extended barrier precautions for prevention of gram-negative bloodstream infections. Infection rates were identical between groups (0.5% vs 0.5%; risk difference −0.03%, 95% CI −0.43 to 0.38; noninferiority P<0.001). Transmission events were also similar between strategies (2.5% vs 3.0%; risk difference −0.44%, 95% CI −2.47 to 1.58). Overall infection rates did not differ meaningfully between groups (2.1% vs 2.0%). These findings indicate no added benefit of routine gown-and-glove precautions over standard hand hygiene in this setting. | Low–moderate risk: Strong cluster-randomized crossover design across multiple NICUs with large sample size enhances external validity. Prespecified noninferiority margin and CONSORT-adherent reporting strengthen methodological rigour. Lack of individual-level randomisation may introduce cluster-level confounding, though crossover design mitigates this. Open-label nature is unavoidable but outcome (bloodstream infection) is objective and registry-defined, reducing detection bias. Some potential for implementation variability across sites. |
| [Chang VWL, Li Q, Barnes D, Byrne AL, Cho JG, Foo SW, et al. Three months of weekly rifapentine and isoniazid versus four months of daily rifampicin for tuberculosis infection: a randomized controlled trial. Clin Infect Dis. 2026 May 15; doi:10.1093/cid/ciag306](https://ad-id.co/4nVBLTf) | **P:** 210 participants with tuberculosis infection (TBI) recruited from 7 tuberculosis clinics in Sydney, Australia (all ages)  **I:** 3 months of weekly isoniazid plus rifapentine (3HP) with SMS adherence reminders  **C:** 4 months of daily rifampicin (4RIF) with standard clinic follow-up  **O:** Treatment completion defined as ≥90% of prescribed doses ingested; adverse events; safety outcomes | Treatment completion was significantly higher with 3HP compared with 4RIF (84.9% vs 65.4%; RR 1.30, 95% CI 1.22–1.38; P<0.001). Adverse event rates were similar between groups (24.5% vs 20.2%), and no treatment-related deaths occurred. Overall, the weekly 3HP regimen with SMS support improved adherence without increasing safety concerns compared with daily rifampicin. | Moderate risk: Open-label design may introduce performance bias, particularly given differing dosing schedules and SMS adherence support in the intervention arm. Randomisation with site stratification and intention-to-treat analysis strengthen internal validity. Objective primary outcome (dose ingestion ≥90%) reduces detection bias, though self-administered dosing and self-reporting may introduce adherence misclassification. Sample size is relatively small, limiting precision for safety outcomes. |
| [Cicuttini FM, Wluka AE, Pan F, O’Sullivan R, Leder K, Cheng AC, et al. Efficacy of antibiotics for chronic low back pain with disc herniation: a randomized clinical trial. JAMA Netw Open. 2026 May;9(5):e2612848. doi:10.1001/jamanetworkopen.2026.12848](https://ad-id.co/4fO6WOc) | **P:** 170 community-based adults aged 18–60 years with chronic low back pain and MRI-confirmed disc herniation (with subgroup analysis including Modic changes)  **I:** Amoxicillin–clavulanate 500 mg/125 mg twice daily for 90 days  **C:** Identical placebo for 90 days  **O:** Pain intensity on Low Back Pain Rating Scale (0–10) at 12 months (primary); pain at 3 months; adverse events | Amoxicillin–clavulanate did not reduce pain compared with placebo at 12 months (adjusted difference 0.06; 95% CI −0.58 to 0.70) or at 3 months (adjusted difference 0.34; 95% CI −0.18 to 0.86). No benefit was observed in prespecified subgroup analyses including patients with Modic changes. Adverse events were more frequent in the antibiotic group (40.0% vs 23.5%), with one serious adverse event in each group possibly related to treatment. Overall, antibiotics provided no clinical benefit for chronic disc-related low back pain and were associated with higher adverse event rates. | Low risk: Double-blind, placebo-controlled randomized design with allocation concealment and intention-to-treat analysis reduces performance, detection, and selection bias. Adequate follow-up (89.4% at 12 months) limits attrition bias. Telemedicine adaptations due to COVID-19 may introduce minor variability in outcome assessment but primary outcome is patient-reported and standardized. Subgroup analyses increase risk of spurious findings but do not affect primary conclusion. |
| [Ornowska M, Wittmann J, Blitz S, Wong H, Vazquez-Grande G, Mitra AR, et al. 4% tetrasodium EDTA to prevent central venous access device–associated complications: a randomized clinical trial. JAMA. 2026 May 18; doi:10.1001/jama.2026.6025](https://ad-id.co/4dEVNhu) | **P:** 1468 adult intensive care unit patients (>18 years) with central venous access devices (CVADs) across 6 Canadian hospitals in a cluster-randomized crossover trial  **I:** 4% tetrasodium EDTA (t-EDTA) locking fluid instilled into unused CVAD lumens  **C:** Saline or 4% citrate locking fluid (standard care, depending on line type)  **O:** Composite CVAD complications including catheter-associated bloodstream infection, catheter occlusion requiring alteplase, and catheter removal due to occlusion | t-EDTA locking fluid significantly reduced the composite rate of CVAD complications compared with control (13.1 vs 19.9 per 1000 catheter-days; adjusted rate ratio 0.68, 95% CI 0.47–0.96; p=0.03). The reduction was primarily driven by fewer catheter occlusions requiring alteplase (11.67 vs 17.73 per 1000 catheter-days; rate ratio 0.66, 95% CI 0.46–0.96), while bloodstream infection rates did not significantly differ between groups. Overall, t-EDTA improved catheter patency and reduced composite CVAD-related complications. | Low–moderate risk: Strong multicentre cluster-randomized crossover design with triple blinding (patients, clinicians, and outcome assessors) reduces selection and performance bias. Objective outcomes (infection, occlusion, catheter removal) strengthen reliability. However, cluster design may introduce residual confounding and period effects despite crossover. Composite endpoint driven mainly by occlusion rather than infection may overestimate clinical impact on infectious outcomes. Minor risk of implementation variability across ICUs. |
| [Rossotti R, Baiguera C, Bernasconi DP, Bana NB, Nava A, Cavazza G, et al. A randomized clinical trial to compare moxifloxacin versus azithromycin for the treatment of Mycoplasma genitalium: the FARTHEST study. Clin Infect Dis. 2026 May 17; doi:10.1093/cid/ciag317](https://ad-id.co/4fe6XuL) | **P:** 358 adults with *Mycoplasma genitalium* infection detected by multiplex PCR in a monocentric setting  **I:** Moxifloxacin 400 mg once daily for 10 days  **C:** Azithromycin 500 mg once daily for 6 days  **O:** Microbiological cure at ≥28 days after treatment completion (test of cure) in ITT and per-protocol populations | Moxifloxacin achieved significantly higher microbiological cure rates than azithromycin in the ITT analysis (87.0% vs 61.2%; absolute risk difference 25.8%, 95% CI 16.5–35.2), with superiority confirmed in per-protocol analysis. Moxifloxacin was consistently more effective across most subgroups, while azithromycin showed comparable efficacy only in heterosexual participants. Both regimens were well tolerated with minimal discontinuation. Overall, moxifloxacin demonstrated clear superiority for eradication of *M. genitalium* infection in the absence of resistance testing. | Moderate risk: Open-label design may introduce performance and adherence bias, although microbiological cure (objective PCR-based outcome) reduces detection bias. Randomization supports internal validity, but monocentric setting limits generalisability. Lack of resistance testing may introduce unmeasured confounding related to baseline resistance patterns. Subgroup analyses increase risk of spurious findings. |
| [Mitanchez D, Lacoste-Badie R, Flamant C, Beuchée A, Tourneux P, Mokhtari M, et al. Procalcitonin-guided decision and antibiotic treatment duration in late onset sepsis of newborns: multicentre, randomised controlled trial (ProABIS). BMJ Med. 2026;5(1):e002602. doi:10.1136/bmjmed-2026-002602](https://ad-id.co/4nX2UFd) | **P:** 504 neonates (>24 weeks gestation, >700 g, postnatal age >4 days) with suspected or proven late-onset sepsis requiring antibiotics >48 hours across 33 neonatal units in France  **I:** Procalcitonin-guided antibiotic decision-making with serial PCT monitoring and recommendation to discontinue antibiotics when PCT ≤0.5 µg/L  **C:** Usual care based on local protocols without procalcitonin guidance  **O:** Duration of antibiotic therapy (primary); mortality at day 28; infection recurrence | Procalcitonin-guided therapy significantly reduced antibiotic duration compared with usual care (median 8 vs 10 days; absolute difference −2.0 days, P<0.001). There was no evidence of harm, with similar 28-day mortality (2.4% vs 3.9%; absolute difference −1.5%, 95% CI −5.0 to 1.8) and similar recurrence rates (2.8% vs 3.9%; absolute difference −1.1%, 95% CI −4.6 to 2.3). Overall, PCT-guided management safely reduced antibiotic exposure in neonatal late-onset sepsis. | Moderate risk: Open-label design may introduce performance bias, particularly in decisions regarding antibiotic discontinuation, although objective mortality and recurrence outcomes reduce detection bias. Multicentre randomised design strengthens external validity. Lack of blinding and reliance on a non-binding recommendation could influence clinician behaviour and adherence to protocol. However, low event rates and consistent safety outcomes support robustness of findings. |
| [Quilty BJ, Toizumi M, Nguyen HAT, Le LT, Le HH, Iwasaki C, et al. Sustained and indirect effects of PCV10 reduced-dose schedules on pneumococcal carriage in Viet Nam: a long-term follow-up of a cluster-randomised controlled trial. Lancet Infect Dis. 2026 May 27; doi:10.1016/S1473-3099(26)00172-6](https://ad-id.co/4dUXAOg) | **P:** 49,644 participants from 24 communes in Nha Trang, Viet Nam, including 10,423 infants (4–11 months), 10,988 toddlers (14–24 months), 9,580 preschool children (3–4 years), and 18,653 adult caregivers in a cluster-randomised controlled trial  **I:** Reduced-dose pneumococcal conjugate vaccine schedule 1p+1 (doses at 2 and 12 months)  **C:** Standard-dose PCV10 schedules (2p+1 and 3p+0); additional comparisons with 0p+1 and unvaccinated communes  **O:** Vaccine-type pneumococcal nasopharyngeal carriage prevalence at 5.5 years; indirect effects in unvaccinated age groups; safety outcomes | The 1p+1 schedule remained non-inferior to standard 2p+1 and 3p+0 schedules for vaccine-type carriage in both infants and toddlers at 5.5 years, with all confidence intervals within the prespecified 5% non-inferiority margin. Vaccine-type carriage declined substantially over time across all groups, including unvaccinated adult caregivers and preschool children, suggesting strong indirect effects regardless of dosing schedule. The lowest carriage proportions were consistently observed across vaccinated groups with minimal between-group differences. No vaccine-related serious adverse events were reported. Overall, reduced-dose schedules maintained long-term effectiveness and population-level protection comparable to standard regimens. | Low–moderate risk: Strong cluster-randomised design with long-term follow-up and large population base enhances external validity. Prespecified non-inferiority framework and clear microbiological outcomes reduce analytic bias. However, cluster-level allocation may introduce residual confounding and ecological effects, and unvaccinated comparison communes may differ systematically. Open-label vaccination status is unavoidable but outcome measurement (nasopharyngeal carriage) is objective. Loss to follow-up and sampling variability across years may introduce some uncertainty in long-term estimates. |
| [Wiesenfeld HC, Hong J, Xu T, Cuffe KM, Bernstein KT, Gift TL, et al. A staff-directed electronic medical record alert to increase chlamydia screening: a randomized clinical trial. JAMA Netw Open. 2026 May 29;9(5):e2615360. doi:10.1001/jamanetworkopen.2026.15360](https://ad-id.co/43DLdkO) | **P:** 18,028 office encounters among women aged 18–24 years (7,356 primary care encounters and 10,672 obstetrics-gynecology encounters) in a cluster-randomized trial across outpatient practices in western Pennsylvania  **I:** Real-time electronic medical record alert directed to medical assistants for women without documented chlamydia screening within the previous year  **C:** Usual care without electronic alert  **O:** Chlamydia screening test orders during office visits | In primary care practices, chlamydia testing was ordered more frequently in the alert group than in the control group (13.2% [497/3770] vs 3.8% [135/3586]). After adjustment for baseline screening differences, the alert significantly increased the odds of test ordering (AOR 2.74, 95% CI 1.94–3.88). The intervention was effective for both reproductive health–related visits (AOR 2.80, 95% CI 1.70–4.62) and visits for other medical reasons (AOR 2.94, 95% CI 1.78–4.85). No significant effect was observed in obstetrics-gynecology practices. | Moderate risk: The cluster-randomized design strengthens internal validity and reduces contamination between intervention and control practices. However, the intervention was not blinded, creating potential performance bias. The primary outcome (test ordering) was objectively captured through electronic medical records, minimizing detection bias. Baseline differences in screening rates required statistical adjustment, and the pragmatic single-region setting may limit generalizability to other healthcare systems. Attrition bias is likely low because outcomes were obtained from routine electronic records. |
| [Dawson R, Diacon AH, Variava E, Moloanto T, Brumskine W, Ngwanto T, et al. Efficacy and safety of a 4-month quabodepistat, delamanid, and bedaquiline regimen for drug-susceptible pulmonary tuberculosis: a multicentre, open-label, randomised, proof-of-concept, non-inferiority, phase 2b/c trial. Lancet Infect Dis. 2026 May 29; doi:10.1016/S1473-3099(26)00143-X](https://ad-id.co/43GGHSu) | **P:** 122 adults aged 18–65 years with newly diagnosed drug-susceptible pulmonary tuberculosis recruited from six sites in South Africa; 121 participants received at least one dose and were included in the modified intention-to-treat analysis  **I:** Four months of delamanid plus bedaquiline plus quabodepistat (DBQ) at one of three dose levels: quabodepistat 10 mg, 30 mg, or 90 mg once daily  **C:** Six months of standard therapy (RHEZ: rifampicin, isoniazid, ethambutol, and pyrazinamide for 8 weeks followed by rifampicin and isoniazid for 18 weeks)  **O:** Primary efficacy outcome: sputum culture conversion by end of treatment; safety and tolerability | End-of-treatment sputum culture conversion was achieved in 100.0% (20/20) of participants in the DBQ10 group, 92.9% (39/42) in the DBQ30 group, 97.4% (37/38) in the DBQ90 group, 96.0% (96/100) in the pooled DBQ group, and 100.0% (21/21) in the RHEZ group. The pooled DBQ regimen met the prespecified non-inferiority criterion versus standard therapy (difference −4.0%, 80% CI −7.4 to 3.4; non-inferiority margin 12%). Adverse events were predominantly mild to moderate, with no treatment-related serious adverse events or discontinuations. Grade ≥3 adverse events occurred in 20% of DBQ10, 14% of DBQ30, 11% of DBQ90, and 5% of RHEZ participants. One death in the DBQ90 group due to worsening tuberculosis with pneumonia was considered unrelated to treatment. Overall, the 4-month DBQ regimen demonstrated promising efficacy and acceptable tolerability compared with the standard 6-month regimen. | Moderate risk: The randomized design supports internal validity, and microbiology laboratory staff were blinded to treatment allocation, reducing outcome assessment bias. However, the open-label design introduces potential performance and detection bias. The proof-of-concept nature, small sample size (particularly the control arm with only 21 participants), and use of an 80% confidence interval for the non-inferiority analysis limit precision and robustness of conclusions. The primary endpoint was a surrogate microbiological outcome measured at end of treatment rather than long-term relapse-free cure, limiting assessment of durable efficacy. |
| [Freiberg JA, Qian ET, Nairon KG, Ereshefsky BJ, Hennessy C, Van Winkle G, et al. Swab testing to optimize pneumonia treatment with empiric vancomycin: a randomized controlled trial. Clin Infect Dis. 2026 Jun 2; doi:10.1093/cid/ciag295](https://ad-id.co/4vM2xzY) | **P:** 277 adult ICU patients with suspected community-acquired pneumonia (CAP) requiring empiric vancomycin therapy  **I:** Addition of MRSA nasal PCR testing after ICU admission to guide vancomycin de-escalation  **C:** Usual care without MRSA nasal PCR testing  **O:** Primary outcome: vancomycin-free hours alive within the first 7 days; secondary outcomes included duration of vancomycin use and 30-day mortality | MRSA nasal PCR testing demonstrated a high negative predictive value (98.9%) for ruling out MRSA pneumonia. However, there was no significant improvement in the primary outcome of vancomycin-free hours alive, with 109.7 hours in the intervention group versus 105.7 hours in the control group (adjusted difference 4.0 hours, 95% CI −9.5 to 18.2; P=0.458). MRSA PCR-guided management also did not reduce overall vancomycin exposure or 30-day mortality compared with usual care, suggesting that availability of PCR results alone was insufficient to change prescribing behavior in this ICU population. | Moderate risk: Randomized prospective trial design strengthens internal validity, but the non-blinded, single-center design could introduce performance bias and limit generalizability. Clinician awareness of group allocation may have influenced antibiotic prescribing decisions. The sample size was moderate, but the study may have been underpowered to detect small differences in vancomycin use. Low risk of attrition bias is suggested by the objective outcome measures and pragmatic trial conduct. The findings may reflect implementation challenges rather than lack of diagnostic utility of MRSA PCR testing. |
| [White NM, Russo PL, Matterson G, Browne K, Cheng AC, Kiernan M, et al. Effectiveness of oral care for the prevention of non-ventilator hospital-acquired pneumonia (HAPPEN): a multicentre, stepped-wedge, cluster-randomised trial in Australia. Lancet Infect Dis. 2026 Jun 8; doi:10.1016/S1473-3099(26)00235-5](https://ad-id.co/3QlPTZu)   - **Editorial Commentary:**   [Cilloniz C, Klompas M. Can better oral care reduce non-ventilator hospital-acquired pneumonia? Lancet Infect Dis. 2026 Jun 8; doi:10.1016/S1473-3099(26)00282-3](https://ad-id.co/4vho1oS) | **P:** 8,870 hospitalized adult patients (≥18 years) admitted for at least 48 hours across 9 wards in 3 Australian hospitals; 4,523 exposed to usual care and 4,347 exposed to the intervention  **I:** Enhanced oral care intervention comprising improved oral care delivery and products, patient and staff education, and audit and feedback delivered by dedicated research nurses  **C:** Usual oral care practice according to hospital policy  **O:** Primary outcome: incidence of non-ventilator hospital-acquired pneumonia (NV-HAP); secondary outcomes: hospital-acquired lower and upper respiratory tract infections and oral cavity infections | NV-HAP occurred in 78/8,870 patients (0.9%), including 46/4,523 (1.0%) in the control group and 32/4,347 (0.7%) in the intervention group. Enhanced oral care significantly reduced NV-HAP incidence, with a cumulative hazard ratio (HR) of 0.40 (95% CI 0.19–0.82), corresponding to a reduction from 1.00 to 0.41 infections per 100 admission-days at risk. No significant differences were observed for lower or upper respiratory tract infections (HR 1.64, 95% CI 0.61–4.39) or oral cavity infections (HR 1.08, 95% CI 0.66–1.77). Adherence to the oral care protocol improved substantially following intervention implementation, increasing from 15.9% (474/2,988) to 61.9% (1,727/2,791). | Moderate risk: The cluster-randomized stepped-wedge design and multicenter implementation strengthen external validity and reduce selection bias. Outcome assessors were blinded, limiting detection bias. However, the intervention was not blinded to staff or patients, introducing potential performance bias. Although adherence improved markedly, it remained incomplete, which may have diluted treatment effects. The relatively low event rate and reliance on medical record review for outcome ascertainment may introduce some misclassification risk. Overall, the large sample size and pragmatic design support the robustness and generalizability of the findings. |
| [Sivanandam P, Syenina A, Chan YFZ, Ng DHL, Gan ES, Yee JX, et al. Aminoglycosides induce immune activation but is insufficient for systemic viral control: a double-blind randomized-controlled clinical trial. Commun Med. 2026 May 23; doi:10.1038/s43856-026-01678-4](https://ad-id.co/4vJyKIk) | **P:** 32 healthy adults in Singapore randomized 1:1 and challenged with live-attenuated yellow fever 17D (YF17D) virus  **I:** Single-dose intravenous gentamicin 5 mg/kg administered before YF17D challenge  **C:** Placebo administered before YF17D challenge  **O:** Primary outcome: proportion of participants with detectable YF17D RNAemia; secondary outcomes: RNAemia levels, symptom rates, neutralizing antibody titers, and innate immune transcriptomic responses | Intravenous gentamicin did not significantly reduce detectable YF17D RNAemia compared with placebo (62.5% [10/16] vs 68.8% [11/16], p>0.99), nor did it affect mean RNAemia levels (3.93 vs 3.51 log10 AUC, p=0.81), symptom rates (56% vs 25%, p=0.15), or neutralizing antibody titers (mean log10 PRNT50 titer 3.89 vs 3.71, p=0.37). Despite the absence of measurable antiviral efficacy, transcriptomic analyses demonstrated upregulation of interferon-stimulated genes (RSAD2, OASL) and transcription factor IRF3 in the gentamicin group, accompanied by increased expression of immune mediators including CCL2, CXCL12, and IL-27, suggesting modest immunomodulatory activity without clinically significant antiviral effects. | Low to moderate risk: The randomized, double-blind, placebo-controlled design minimizes selection, performance, and detection bias. Objective virologic and immunologic endpoints further strengthen internal validity. However, the small sample size (n=32) substantially limits statistical power and increases the risk of type II error, potentially obscuring modest clinical effects. The single-center setting and use of healthy volunteers rather than patients with natural viral infection may limit generalizability. Overall, the trial is methodologically robust but exploratory in nature due to its limited sample size and experimental challenge model. |
| [Fehring TK, Otero JE, Fehring KA, Curtin BM, Springer BD, Della Valle CJ, et al. One-stage versus two-stage exchange arthroplasty for periprosthetic joint infection: a prospective randomized trial. J Bone Joint Surg. 2026 Apr 1; doi:10.2106/JBJS.25.00713](https://ad-id.co/4fLGDse) | **P:** 323 patients with chronic periprosthetic joint infection (PJI) following primary total hip or knee arthroplasty and a known causative organism; included patients with draining sinuses, comorbidities, and antibiotic-resistant organisms. After deaths and losses to follow-up, 258 patients (135 one-stage, 123 two-stage) had 2-year follow-up.  **I:** One-stage revision arthroplasty with implant exchange and standardized irrigation and antibiotic protocols  **C:** Two-stage revision arthroplasty with standardized irrigation and antibiotic protocols  **O:** Clinical success at 2 years, defined as no clinical failure or reinfection, no reoperation for PJI, and no PJI-related death | At 2 years, one-stage revision achieved a higher clinical success rate than two-stage revision (97% [131/135] vs 91% [112/123]; p=0.04). Patients undergoing one-stage treatment had more than threefold greater odds of overall success compared with two-stage treatment (unadjusted OR 3.22, 95% CI 1.0–10.38). This benefit remained after adjustment for McPherson host grade, presence of resistant organisms, and draining sinuses. Sixteen patients in the one-stage group and nine in the two-stage group died before 2-year follow-up. Overall, the findings demonstrated that one-stage revision was noninferior to two-stage revision when performed using the specified treatment protocol in selected patients with chronic PJI. | Moderate risk: The prospective, multicenter randomized design strengthens internal validity and improves generalizability compared with previous studies. Inclusion of patients with resistant organisms, draining sinuses, and comorbidities enhances real-world applicability. However, attrition due to death and loss to follow-up reduced the evaluable population from 323 to 258 patients, introducing potential attrition bias. Blinding was likely not feasible given the nature of the surgical interventions, creating a risk of performance and detection bias. The analysis was based on patients with available follow-up rather than a strict intention-to-treat approach, which may slightly overestimate treatment success. Nonetheless, balanced baseline characteristics and consistent protocols across groups support the robustness of the findings. |
| [Angkanavisan K, Sakboonyarat B, Ungthammakhun C. Oral fosfomycin after carbapenems as de-escalating therapy in complicated urinary tract infections: a randomized controlled trial. Int J Infect Dis. 2026 May 29; doi:10.1016/j.ijid.2026.108826](https://ad-id.co/4w9FUGf) | **P:** 124 adults with complicated urinary tract infections (cUTIs) caused by third-generation cephalosporin-resistant Enterobacterales (3GCRE) who had received 3 days of intravenous carbapenem therapy; mean age 73 years, 62.1% female, 91% caused by *Escherichia coli*  **I:** Oral fosfomycin trometamol step-down therapy to complete a 7-day treatment course  **C:** Continued carbapenem therapy to complete a 7-day treatment course  **O:** Primary outcome: clinical cure at day 7; secondary outcomes: clinical cure at day 28, microbiological cure at days 7 and 28, readmission, and hospital length of stay | Both groups achieved a cure rate of 98.4% (risk difference 0.0, 95% CI −0.04 to 0.04). Clinical cure at day 28 was also identical in both groups (91.9%). Microbiological cure rates were comparable, with day-7 cure achieved in 100% of fosfomycin-treated patients versus 98.1% of carbapenem-treated patients, and day-28 cure in 90.3% versus 85.5%, respectively. | High risk: The trial design has many limitations. The open-label design introduces potential performance and detection bias, particularly for subjective clinical outcomes. The sample size was small (124 patients), and its justification poorly defined. The predominance of *E. coli* infections (91%) may reduce generalizability to other Enterobacterales species. |
| [Adubra L, Luoma J, Fan YM, Cheick Haidara F, Samaké O, Ihamuotila R, et al. Infant growth after mass administration of azithromycin: secondary outcomes of a cluster randomized clinical trial. JAMA Netw Open. 2026 Jun 10;9(6):e2617425; doi:10.1001/jamanetworkopen.2026.17425](https://ad-id.co/3Q2GVAx) | **P:** 1,205 infants aged 1–11 months from 1,151 villages in southwestern Mali enrolled in a cluster-randomized trial; growth substudy assessed 1,789 children aged 6–8 months and 12–14 months at follow-up  **I:** Mass administration of azithromycin (20 mg/kg orally) given quarterly to infants aged 1–11 months  **C:** Placebo at all treatment rounds or twice-yearly azithromycin (azithromycin during January–June rounds and placebo during July–December rounds)  **O:** Growth outcomes including weight, length, weight-for-age (WA), length-for-age (LA), weight-for-length (WL), and mid-upper-arm circumference (MUAC) z scores, as well as prevalence of underweight, stunting, and wasting | In this prespecified secondary analysis, neither quarterly nor twice-yearly azithromycin improved infant growth compared with placebo. Mean weight, length, WA, LA, WL, and MUAC z scores were similar across all groups. For example, mean weight was 7.8 kg in all groups, and WA z scores were −1.12, −1.10, and −1.12 in the placebo, twice-yearly, and quarterly azithromycin groups, respectively. Likewise, the prevalence of underweight, stunting, and wasting did not differ between treatment groups. Overall, mass azithromycin administration showed no measurable benefit on anthropometric growth outcomes despite previous evidence suggesting reductions in childhood mortality. | Low risk: This was a large cluster-randomized clinical trial with masked participants and study personnel, reducing selection, performance, and detection bias. Outcomes were prespecified and analyzed on an intention-to-treat basis, further strengthening validity. Anthropometric measurements are objective and less susceptible to assessment bias. Limitations include the growth substudy being conducted in only 59 villages and the use of cross-sectional rather than longitudinal growth assessments, which may reduce sensitivity to detect small growth effects. However, the large sample size, rigorous methodology, and consistency of findings across multiple growth indicators support a high degree of confidence in the results. |
| [Gohil SK, Avery TR, Kleinman K, Septimus E, Mauricio A, Sands KE, et al. Improving empiric antibiotic selection for patients with cancer hospitalized with infection: secondary analysis of the INSPIRE cluster randomized trials. JAMA Netw Open. 2026 Jun 10;9(6):e2616611; doi:10.1001/jamanetworkopen.2026.16611](https://ad-id.co/4vOYnYi) | **P:** 36,861 non–critically ill hospitalized adults with hematologic or solid organ malignancies and community-acquired pneumonia, urinary tract infection (UTI), skin and soft tissue infection (SSTI), or abdominal infection enrolled in four INSPIRE cluster-randomized clinical trials (18,272 baseline and 18,589 intervention patients)  **I:** Antibiotic stewardship bundle incorporating computerized provider order entry (CPOE) prompts using real-time patient-specific MDRO risk estimates, recommending standard-spectrum antibiotics when predicted MDRO risk was <10%, plus education and feedback  **C:** Usual prescribing practices without CPOE-guided stewardship prompts  **O:** Primary outcome: extended-spectrum antibiotic days of therapy; secondary outcomes: ICU transfer, hospital length of stay, hospital readmission, and in-hospital mortality | CPOE-guided stewardship significantly reduced empiric extended-spectrum antibiotic use across all infection syndromes. Extended-spectrum antibiotic days of therapy decreased by 27% for pneumonia (RR 0.73, 95% CI 0.67–0.80; P<0.001), 24% for UTI (RR 0.76, 95% CI 0.68–0.84; P<0.001), 17% for SSTI (RR 0.83, 95% CI 0.74–0.92; P<0.001), and 24% for abdominal infections (RR 0.76, 95% CI 0.69–0.84; P<0.001). Importantly, reductions in broad-spectrum antibiotic use were not associated with increases in ICU transfers, hospital length of stay, readmissions, or in-hospital mortality, supporting the safety of this targeted stewardship approach in hospitalized patients with cancer. | Moderate risk: The analysis leveraged four large cluster-randomized clinical trials, providing a robust sample size and enhancing generalizability across multiple infection syndromes. Objective outcomes such as antibiotic utilization and hospital outcomes reduce detection bias. However, this was a secondary analysis restricted to patients with cancer rather than a trial specifically randomized within the cancer population, introducing potential subgroup-analysis limitations. Cluster-randomized designs may also be susceptible to contamination and secular trends despite adjusted difference-in-difference analyses. The absence of observed safety differences is reassuring, although the study may not have been powered specifically for rare adverse clinical outcomes. Overall, the large sample size and consistent findings across trials support moderate-to-high confidence in the results. |
| [Hamani M, Pinto R, Lee TC, MacFadden D, McDonald EG, Sligl W, et al. Duration of antibiotic therapy for bacteremia in immune compromised hosts—a post hoc subgroup analysis of the BALANCE randomized clinical trial. Clin Microbiol Infect. 2026 Jun 9; doi:10.1016/j.cmi.2026.06.003](https://ad-id.co/4emJPrD) | **P:** 1,037 immunocompromised adults with bacteremia enrolled in the BALANCE randomized clinical trial; 530 randomized to 7 days and 507 to 14 days of antibiotic therapy. Common immunocompromising conditions included solid malignancies (75.4%), leukemia/lymphoma (9.7%), chemotherapy use (15.4%), corticosteroid therapy (4.7%), HIV/AIDS (2.3%), and a small number of neutropenic patients (2.5%).  **I:** 7 days of appropriate antibiotic therapy for bacteremia  **C:** 14 days of appropriate antibiotic therapy for bacteremia  **O:** Primary outcome: all-cause 90-day mortality; sensitivity analysis in the modified intention-to-treat population surviving at least 7 days | Among immunocompromised patients with bacteremia, shorter-duration therapy produced outcomes comparable to longer treatment. In the intention-to-treat analysis, 90-day mortality was 20.1% (106/528) in the 7-day group versus 23.7% (120/507) in the 14-day group (risk difference −3.6%, 95% CI −8.6% to 1.4%; p=0.16). Similar findings were observed in the modified intention-to-treat analysis, with mortality of 19.8% versus 23.2%, respectively (risk difference −3.4%, 95% CI −8.5% to 1.6%; p=0.18). Patients received a median of 8 days versus 14 days of antibiotics in the two groups. Overall, there was no evidence that longer treatment improved survival, supporting the adequacy of a 7-day treatment course in this subgroup. | Moderate risk: This was a post-hoc subgroup analysis of a large randomized controlled trial, so randomization was preserved but the analysis was not prespecified for immunocompromised patients and may have been underpowered to detect smaller subgroup-specific differences. The intention-to-treat and modified intention-to-treat analyses yielded consistent results, strengthening reliability. However, the immunocompromised population was heterogeneous and predominantly composed of patients with solid malignancies, limiting applicability to severely immunosuppressed groups such as neutropenic patients, transplant recipients, or those with profound immune dysfunction. As a post-hoc analysis, findings should be considered hypothesis-supporting rather than definitive for all immunocompromised populations. |
| [Beales E, Clements MN, Feasey NA, Lissauer D, Banda M, Maseko B, et al. Antiseptic cleansing to reduce vertical transmission of pathogens to neonates: the NeoVT-AMR randomized clinical trial. JAMA Netw Open. 2026 Jun 11;9(6):e2615665. doi:10.1001/jamanetworkopen.2026.15665](https://ad-id.co/4a2BJ6p) | **P:** 149 laboring women and 147 facility-born neonates (<24 hours old, birth weight >1000 g) in Malawi  **I:** Topical antisepsis with chlorhexidine 1%, chlorhexidine 2%, or octenidine 0.1% with phenoxyethanol 2%, applied once or multiple times during labour/postpartum or neonatal period  **C:** Standard of care (sterile water for mothers; no cleansing for neonates) and between-arm comparisons of different antiseptics and dosing frequencies  **O:** Change in skin bacterial load (log10 CFU), skin condition scores, serious adverse events, neonatal temperature | In mothers, 1% chlorhexidine significantly reduced bacterial load compared with octenidine (adjusted difference +1.7 log10 CFU worse with OHP, 95% CI 0.9–2.5) and standard care (+3.5, 95% CI 2.4–4.6), while showing no clear difference vs 2% chlorhexidine (−0.6, 95% CI −1.4 to 0.2). In neonates, 1% chlorhexidine was more effective than standard care (difference +1.3 log10 CFU, 95% CI 0.2–2.4) but similar to 2% CHG and OHP. Repeated applications did not clearly improve outcomes overall, although frequency×time interaction suggested increasing benefit in neonates. No meaningful differences in skin adverse effects or serious adverse events were observed, and no neonatal hypothermia signal was detected. Overall, 1% chlorhexidine appeared effective at reducing colonisation in both populations without safety concerns. | Moderate risk: Open-label factorial design introduces potential performance bias, although laboratory staff were blinded to outcomes, reducing detection bias. Randomisation strengthens internal validity. Relatively small sample size and exploratory, multi-arm factorial structure may reduce power for between-group comparisons. Attrition and safety follow-up were adequate with no major losses reported, but generalisability beyond a single-site Malawian hospital may be limited. |
| [Yende S, Talisa VB, Mayes K, Williams K, Malito A, Mayr FB, et al. Remote monitoring approaches to reduce readmissions after infection and sepsis: a randomized clinical trial. JAMA Netw Open. 2026 Jun 11;9(6):e2616641. doi:10.1001/jamanetworkopen.2026.16641](https://ad-id.co/3QEMm8U) | **P:** 1,286 adults discharged after hospitalization for sepsis or lower respiratory tract infection across 19 hospitals (median age 63 years; 32.6% ICU-treated)  **I:** Remote monitoring (RPM) combining low- vs high-intensity questionnaires with standard vs enhanced clinical response teams (4 active strategies)  **C:** Usual postdischarge care  **O:** Primary outcome was days alive at home at 90 days; secondary outcomes included readmission, mortality, functional status, quality of life, and healthcare utilisation | Remote monitoring did not increase days alive at home compared with usual care, with median 90 home days across all arms and no meaningful differences in cumulative odds ratios (0.86–1.01 vs usual care; credible intervals all crossing null). Readmission rates were similar across groups (approximately 36%–44%). In patients ≥65 years, both standard and enhanced RPM were associated with fewer home days than usual care (COR 0.56 and 0.67, respectively), with high probabilities of inferiority. Overall, RPM as implemented did not improve postdischarge outcomes and may have been harmful in older adults. | Moderate risk: Large pragmatic multicentre randomised design with adequate sample size strengthens internal validity, but substantial non-adherence (only ~60% enrolment in RPM arms) introduces potential dilution bias. Response-adaptive randomisation may introduce imbalance over time. Blinding was not feasible, raising risk of performance and detection bias for patient-reported outcomes. However, objective primary outcome (days at home) reduces outcome measurement bias. Subgroup findings (older adults) may be hypothesis-generating. |
| [Van Rie A, Conceição EC, Ndebele F, Wells F, Paulse A, Mekonnen EG, et al. Whole genome sequencing precision medicine strategy to shorten treatment for rifampicin-resistant tuberculosis (SMARTT): a pragmatic, randomised, single-blind phase 4 trial. Lancet Respir Med. 2026 Jun;14:521-532. doi:10.1016/S2213-2600(26)00095-0](https://ad-id.co/4etI5gB) | **P:** 204 adults with pulmonary rifampicin-resistant tuberculosis in South Africa randomized 1:1; modified intention-to-treat (mITT) analysis included 162 culture-positive participants (84 WGS-guided, 78 standard care). Most participants were male (74%), living with HIV (63%), and had multidrug-resistant tuberculosis (88%).  **I:** Whole genome sequencing (WGS)-guided individualized 4-drug regimen selected using a feature-based artificial intelligence model, targeting a 6-month treatment duration  **C:** Standard of care treatment (WHO-recommended all-oral 9-month regimen or individualized 18-month regimen)  **O:** Primary outcome: bacteriological effectiveness (time to culture conversion/mycobacterial load decline); secondary outcomes: unfavourable treatment outcomes (bacteriological failure, loss to follow-up, or death), treatment duration, and serious adverse events | WGS-guided therapy shortened median treatment duration by 2.6 months and generated 15 individualized regimens. Bacteriological response was similar between groups, with a mean mycobacterial load half-life of 0.30 weeks (95% CI 0.27–0.33) in the WGS group versus 0.31 weeks (95% CI 0.31–0.31) in the standard-care group (relative difference 0.97, 95% CI −0.86 to 1.07; p=0.26). Unfavourable treatment outcomes were significantly less frequent with WGS-guided treatment (24% [20/82] vs 42% [32/77]; adjusted risk difference −18.4 percentage points, 95% CI −35.6 to −1.2; superiority p=0.018), largely driven by lower loss to follow-up. Serious adverse events occurred at similar rates in both groups (27% vs 33%; risk difference −6 percentage points; p=0.41). Overall, WGS-guided therapy achieved comparable bacteriological efficacy, shorter treatment duration, and improved clinical outcomes without additional safety concerns. | Moderate risk: The randomized, multicenter pragmatic phase 4 design enhances external validity and reflects real-world practice. However, the study was single-blind, creating potential performance bias. The primary planned analysis was affected by operational constraints, resulting in reliance on a modified intention-to-treat population rather than the full randomized cohort, which may introduce selection bias. The relatively small sample size and exclusion of culture-negative participants from the efficacy analysis may limit precision and generalizability. Nevertheless, balanced groups, objective microbiological endpoints, and consistent safety findings support the credibility of the results. |
| [Heysell SK, Mpagama SG, Nuwagira E, Said B, Conaway M, Null M, et al. Immediate or high-dose antituberculosis therapy for HIV-related sepsis in Tanzania and Uganda (ATLAS): a phase 3, open-label, randomised, controlled, 2×2 factorial, superiority trial. Lancet Infect Dis. 2026;26(6):601-613. doi:10.1016/S1473-3099(25)00747-9](https://ad-id.co/3SrrLW5) | **P:** 395 adults living with HIV hospitalized with sepsis in Tanzania and Uganda (from 437 randomized participants); 52% subsequently had confirmed tuberculosis  **I:** Immediate empiric antituberculosis therapy initiated at enrollment (conventional-dose or high-dose regimen) and/or high-dose antituberculosis therapy (rifampicin ~30 mg/kg and isoniazid ~7.5 mg/kg) as part of a 2×2 factorial design  **C:** Diagnosis-dependent antituberculosis therapy initiated only after clinical or microbiological diagnosis of tuberculosis and/or conventional WHO-recommended weight-based antituberculosis therapy  **O:** Primary outcome: 28-day mortality; secondary outcomes: mortality among patients with confirmed tuberculosis and adverse events | Among all participants with HIV-associated sepsis, neither immediate empiric antituberculosis therapy nor high-dose antituberculosis therapy reduced 28-day mortality. Mortality was identical in the immediate and diagnosis-dependent groups (25% vs 25%; adjusted HR 0.99, 95% CI 0.67–1.46; p=0.95) and similar between high-dose and conventional-dose groups (26% vs 25%; adjusted HR 1.07, 95% CI 0.72–1.59; p=0.73). However, among participants with microbiologically confirmed tuberculosis, immediate conventional-dose therapy significantly reduced mortality compared with diagnosis-dependent conventional-dose therapy (12% vs 34%; adjusted HR 0.32, 95% CI 0.13–0.82; p=0.015). High-dose therapy did not provide a significant mortality benefit. Adverse event rates were similar across groups, although drug-induced liver injury occurred numerically more often in the immediate high-dose group. | Moderate risk: The multicenter randomized controlled factorial design with concealed allocation strengthens internal validity and allows simultaneous assessment of treatment timing and dosing strategies. However, the open-label design may introduce performance bias, although mortality is an objective endpoint less susceptible to assessment bias. The primary analysis was conducted in a modified intention-to-treat population rather than the full randomized cohort, which may introduce some selection bias. The significant benefit observed in the subgroup with confirmed tuberculosis was based on a secondary subgroup analysis and should be interpreted cautiously. Overall, the trial was well conducted, but subgroup findings require confirmation in future studies specifically targeting patients with confirmed tuberculosis. |
| [The Staphylococcus aureus Network Adaptive Platform (SNAP) Trial Group. Cefazolin for methicillin-susceptible Staphylococcus aureus bacteremia. N Engl J Med. 2026 Jun 17;394(23):2329-2339. doi:10.1056/NEJMoa2506905](https://ad-id.co/4vcSNyV) | **P:** 1,308 adults with penicillin-resistant, methicillin-susceptible *Staphylococcus aureus* bacteremia (645 evaluable in cefazolin group; 642 evaluable in antistaphyloccal-penicillin group)  **I:** Cefazolin  **C:** Antistaphylococcal penicillin (flucloxacillin or cloxacillin)  **O:** Primary: all-cause mortality at 90 days. Secondary: acute kidney injury within 14 days. | Cefazolin met the prespecified criterion for noninferiority for 90-day mortality, with deaths occurring in 15.0% of patients versus 17.0% with flucloxacillin/cloxacillin (adjusted OR 0.81, 95% credible interval [CrI] 0.59–1.12; probability of noninferiority 99.2%; probability of superiority 89.8%). Acute kidney injury occurred less frequently with cefazolin (13.9% vs 19.6%; adjusted OR 0.67, 95% CrI 0.50–0.89), with a 99.7% probability of superiority. Overall, cefazolin was noninferior for survival and demonstrated a superior renal safety profile. | Moderate risk: Randomized design and large sample size strengthen internal validity, but the open-label design may introduce performance bias and influence co-interventions. The primary outcome of 90-day mortality is objective and unlikely to be affected by lack of blinding, reducing detection bias. The adaptive Bayesian platform design is appropriate but adds analytical complexity. Attrition appeared low, and the multicenter international setting enhances generalizability. Overall risk of bias is moderate, primarily due to the open-label nature of the trial. |
| [The Staphylococcus aureus Network Adaptive Platform (SNAP) Trial Group. Benzylpenicillin versus flucloxacillin or cloxacillin for the treatment of penicillin-susceptible Staphylococcus aureus bacteraemia (SNAP): an international, multicentre, open-label, non-inferiority randomised controlled trial. Lancet. 2026 Jun 17; doi:10.1016/S0140-6736(26)00761-0](https://ad-id.co/3Sf7xyN)   - **Editorial Commentary:**   [Westgeest AC, Fowler VG Jr. Treating penicillin-susceptible *Staphylococcus aureus* bacteraemia: confusing the issue with facts. Lancet. 2026 Jun 17; doi:10.1016/S0140-6736(26)00857-3](https://ad-id.co/3QsVLjG) | **P:** 281 adults with penicillin-susceptible *Staphylococcus aureus* (PSSA) bacteraemia from 67 hospitals across eight countries (median age 67 years; 31% female)  **I:** Intravenous benzylpenicillin  **C:** Intravenous flucloxacillin or cloxacillin  **O:** Primary: all-cause mortality at 90 days. Secondary: acute kidney injury (AKI), serious adverse reactions. | At trial closure, 90-day mortality occurred in 14% (21/152) of patients receiving benzylpenicillin versus 22% (26/121) receiving flucloxacillin/cloxacillin (adjusted OR 0.67, 95% CrI 0.35–1.28), corresponding to a 96.1% posterior probability of non-inferiority and 88.9% probability of superiority, although the prespecified non-inferiority threshold was not formally reached. AKI occurred less frequently with benzylpenicillin (11% vs 22%; adjusted OR 0.50, 95% CrI 0.26–0.94), with a 98.4% probability of superiority. Serious adverse reactions were also less common with benzylpenicillin (4% vs 7%). Overall, benzylpenicillin showed similar survival outcomes with substantially improved renal safety. | Moderate risk: The randomized multicentre design strengthens internal validity, but the open-label design introduces potential performance bias and may influence co-interventions or reporting of adverse events. The primary outcome of all-cause mortality is objective and unlikely to be affected by lack of blinding. Early trial termination due to excess AKI in the comparator arm may have reduced statistical power and prevented formal achievement of the prespecified non-inferiority threshold. The Bayesian adaptive platform design is appropriate but complex. Overall risk of bias is moderate, driven mainly by the open-label design and early stopping. |

**Trial Protocols / Trial Ideas**

- [Rose N, Bernasconi NS, Schumacher M, Werlen L, Büchel D, Weisser M, et al. Linezolid in addition to standard antibiotic treatment for *Staphylococcus aureus* bacteraemia: study protocol for a randomised, placebo-controlled trial. BMJ Open. 2026 Apr 20;16(4):e118509. doi:10.1136/bmjopen-2026-118509](https://ad-id.co/4uor3H4)
  - Summary: This multicentre randomized controlled trial in Switzerland will evaluate whether adjunctive linezolid (600 mg twice daily for 5 days) added to standard antibiotic therapy improves patient-centered outcomes, measured by a desirability of outcome ranking (DOOR) at 90 days, in 606 adults with Staphylococcus aureus bacteraemia.

**Phage Therapy**

- [Gordillo Altamirano F, Subedi D, Beiers M, Bucher M, Dahlman S, Patel DM, et al. Cross-reactive anti-prophage antibodies and bacterial heteroresistance implicated in phage therapeutic failure. Nat Med. 2026 Apr 24; doi:10.1038/s41591-026-04301-0](https://ad-id.co/421Lb5F)
  - Summary: At last, a published case which talks about failure of phage therapy. This case study of a 22-year-old patient with cystic fibrosis and fatal Bordetella bronchialis infection found that pre-existing antiphage antibodies and bacterial heteroresistance likely contributed to failure of compassionate-use phage therapy, highlighting the need to assess host immunity and bacterial susceptibility before treatment.

**PK/PD and Drug Dosing**

- [Lodise TP, Turner NA, Hamasaki T, Fishbane N, Ghazaryan V, Wall A, et al. Pharmacokinetics of dalbavancin in complicated *Staphylococcus aureus* bacteraemia: a secondary analysis of the DOTS randomized clinical trial. JAMA Netw Open. 2026 Apr 18;9(4):e2611652. doi:10.1001/jamanetworkopen.2026.11652](https://ad-id.co/4u0LA4J)
  - Summary: This secondary analysis of 97 adults with complicated Staphylococcus aureus bacteraemia found that dalbavancin pharmacokinetics were influenced by kidney function, weight, albumin, and age, and that higher drug exposure (day 22 concentration >32 μg/mL) was associated with improved day 70 clinical success without increased serious adverse events, supporting exploration of exposure-guided dosing.
- [Chiriac U, Münchow M, Röhr AC, Frey OR, Frey AT, Frisch D, et al. Pharmacokinetics, target attainment and outcomes of piperacillin/tazobactam in critically ill patients receiving continuous infusion with therapeutic drug monitoring: a retrospective analysis. Clin Microbiol Infect. 2026 Apr 16; doi:10.1016/j.cmi.2026.04.010](https://ad-id.co/4tJzexJ)
  - Summary: This large study of 1538 critically ill patients receiving continuous piperacillin/tazobactam found that pharmacokinetic/pharmacodynamic-guided dosing with therapeutic drug monitoring improved target attainment (45.7% to 62.4%), minimized subtherapeutic and supratherapeutic exposure, and was associated with lower 28-day mortality in patients within the therapeutic range compared with supratherapeutic levels, supporting individualized dosing strategies in routine care.
- [Gatti M, Bonazzetti C, Secci B, Giorgi B, Rinaldi M, Cojutti PG, et al. Aggressive joint pharmacokinetic/pharmacodynamic target attainment of TDM-guided continuous infusion meropenem-vaborbactam monotherapy: a valuable strategy for maximizing the microbiological outcome of documented KPC-producing Enterobacterales infections? Int J Antimicrob Agents. 2026 Apr 17;107811. doi:10.1016/j.ijantimicag.2026.107811](https://ad-id.co/4uj8L9Q)
  - Summary: This retrospective cohort study of 55 patients with KPC-producing Enterobacterales infections found that therapeutic drug monitoring–guided continuous infusion of meropenem-vaborbactam achieved aggressive PK/PD targets in 96.3% of cases, with microbiological failure in 20.5% and low resistance emergence (4.5%), while continuous renal replacement therapy and intra-abdominal infections were independent predictors of failure.
- [Setiawan E, Gonzalez A, Wölky SA, Cotta MO, Liu X, Sosilya H, et al. Population pharmacokinetics of total and free ceftriaxone in critically ill and non-critically ill hospitalised adult patients. Int J Antimicrob Agents. 2026 Apr 21;107817. doi:10.1016/j.ijantimicag.2026.107817](https://ad-id.co/4uk4XW3)
  - Summary: This pharmacokinetic study of 53 Indonesian patients found that renal function was the main determinant of ceftriaxone exposure, with standard once-daily dosing often inadequate in patients with normal or augmented renal clearance, supporting a renal-adjusted regimen of 1 g every 24 hours for eGFR ≤60 mL/min/1.73 m² and 1 g every 12 hours for eGFR >60 mL/min/1.73 m² to optimize efficacy and safety.

**Antibiotic Stewardship**

- [Russo A, Geremia N, Fiore Bavaro D, Seo SK, Laracy J, Mazzitelli M, et al. Comparing large language models for antibiotic prescribing: an updated analysis using the same clinical benchmark. Clin Microbiol Infect. 2026 Apr 16; doi:10.1016/j.cmi.2026.04.012](https://ad-id.co/3P9HR5u)
  - Summary: This comparative evaluation of five large language models across 60 infectious disease scenarios found that antibiotic prescribing accuracy remained modest (57.3% correct overall) with persistent overtreatment (~22%), with ChatGPT-o1 outperforming newer models, highlighting limited improvement over time and reinforcing the need for clinician oversight in antimicrobial stewardship decisions.

**Infection Prevention / Antibiotic Prophylaxis**

- [Collins CD, Hartsfield E, Cleary RK, Veve MP, Brockhaus KK. β-Lactam vs non–β-lactam prophylaxis in elective colorectal surgery. JAMA Netw Open. 2026 Apr 13;9(4):e266708. doi:10.1001/jamanetworkopen.2026.6708](https://ad-id.co/4uEi5Wj)
  - Summary: This large multicenter cohort study of 20,140 colorectal surgeries found that β-lactam prophylaxis was associated with significantly lower 30-day surgical site infection risk compared with non–β-lactam alternatives (ARR 0.74, 95% CI 0.63–0.87), independent of differences in guideline-concordant dosing and timing.
- [Elangovan S, Senanayake S, Venkatachalam I, Ling ML, Graves N, Kularatna S. Preferences for infection prevention in hospital-acquired infections. JAMA Netw Open. 2026 Apr 15;9(4):e267432. doi:10.1001/jamanetworkopen.2026.7432](https://ad-id.co/4uk4jry)
  - Summary: This global survey study of 256 healthcare professionals found that cost-effectiveness was the most influential factor in selecting infection prevention and control practices, with distinct preferences between “evidence seekers” in low- and middle-income countries prioritizing strong clinical evidence and “efficiency seekers” in high-income countries emphasizing economic value, highlighting important contextual differences in adoption decisions.
- [Hammond NE, Devaux A, Vlok R, Adigbli D, Cuthbertson BH, Finfer SR, et al. Selective decontamination of the digestive tract in adult mechanically ventilated patients—an updated systematic review with Bayesian meta-analysis. NEJM Evid. 2026 Apr 15;5(5). doi:10.1056/EVIDoa2500264](https://ad-id.co/492CADq)
  - Summary: This updated Bayesian meta-analysis of 32 randomized clinical trials including 27,687 mechanically ventilated ICU patients found that selective decontamination of the digestive tract (SDD) was associated with a probable reduction in hospital mortality compared with standard care (RR 0.91, 95% credible interval 0.82–0.99), with a 99.2% posterior probability of benefit.
- [Souza Júnior EV, da Silva Alonso C, Campos CC, Cruz DP. Effects of daily bathing with 2% chlorhexidine compared to bathing with soap and water on risk of death, clinical severity, and intensive care unit length of stay in critically ill patients: an updated systematic review and meta-analysis. Eur J Clin Microbiol Infect Dis. 2026 Apr 18; doi:10.1007/s10096-026-05513-5](https://ad-id.co/4unpd9l)
  - Summary: This systematic review and meta-analysis of 10 studies including 33,860 ICU patients found that daily bathing with 2% chlorhexidine gluconate (CHG) compared with traditional soap and water bathing was not associated with reduced mortality (RR 0.93, 95% CI 0.86–1.00) or improved clinical severity scores, and was linked to a very small but statistically significant increase in ICU length of stay (mean difference 0.07 days), although overall certainty of evidence was low.
- [Phillips C, Bahra M, Metodiev Y, Bamber JH, Ó Cróinín D, Fang L, et al. Use of sterile gowns for single-shot spinal anaesthesia: consensus guidelines from the Association of Anaesthetists, Royal College of Anaesthetists, Obstetric Anaesthetists' Association, Regional Anaesthesia UK, College of Anaesthesiologists of Ireland and Australian and New Zealand College of Anaesthetists. Anaesthesia. 2026 Apr 16; doi:10.1111/anae.70212](https://ad-id.co/4tD2l5v)
  - Summary: This evidence review and modified Delphi consensus involving 32 experts and one patient representative developed updated recommendations for infection prevention during single-shot spinal anaesthesia, concluding that routine use of sterile gowns in uncomplicated adult cases should not be mandatory, while reinforcing essential aseptic measures such as hand hygiene and facemask use to support safe, cost-effective, and environmentally sustainable practice.

**Gastrointestinal Tract Infections**

- [Chapman MM, Cochran RL, Krishnan K, Hysell KM, Alkashash A. Case 12-2026: an 86-year-old woman with anorexia, weight loss, and liver lesions. N Engl J Med. 2026 Apr 22;394:1635-1645. doi:10.1056/NEJMcpc2412525](https://ad-id.co/424EL5O)
  - Summary: This case describes an 86-year-old woman presenting with subacute anorexia, weight loss, cholestatic liver injury, and multifocal hepatic lesions in the setting of choledocholithiasis and biliary ductal dilatation on imaging.
- [Tarr GAM, Finical W, Rounds JM, Panek A, Smith K. The conundrum of Shiga toxin-producing *Escherichia coli* O157:H7 persistence: evidence for locally persistent lineages. J Infect Dis. 2026 Apr 23;jiag223. doi:10.1093/infdis/jiag223](https://ad-id.co/4tHK47h)
  - Summary: This ecological genomics study of Shiga toxin-producing Escherichia coli (STEC) O157:H7 in Minnesota identified 15 locally persistent lineages that persisted for 1.3–8.6 years, accounted for 35.3% of reported cases, were associated with multiple local outbreaks without multi-state spread, and showed spatial clustering, supporting ecosystem-level persistence as a key driver of regional disease burden.
- [Liao TE, Chuang C, Liu SY, Ho YC, Chin W, Chou SH, et al. *Klebsiella pneumoniae* liver abscess as the herald of underlying colorectal neoplasm. Open Forum Infect Dis. 2026 Apr 2;13(4):ofag191. doi:10.1093/ofid/ofag191](https://ad-id.co/3OVCN4C)
  - Summary: This retrospective cohort study of 489 patients with Klebsiella pneumoniae liver abscess in Taiwan found a high prevalence of underlying colorectal neoplasia among those undergoing colonoscopy within 6 months (42.1% adenomas and 11.4% adenocarcinomas), with age ≥50 years being the only independent risk factor, supporting routine colonoscopy after KPLA diagnosis to detect occult colorectal cancer.
- [Chen J, Wang T, Liu S, Xiang J, Qi Y, Zhou H, et al. Performance of a rapid, visual fingerstick serology self-test for *Helicobacter pylori* detection and typing: a prospective multicenter diagnostic accuracy study. Clin Microbiol Infect. 2026 Apr 28; doi:10.1016/j.cmi.2026.04.020](https://ad-id.co/4tKIX6T)
  - Summary: This multicenter diagnostic accuracy study of 1,330 participants evaluated a novel latex agglutination test (LAT) self-testing kit for identifying type I Helicobacter pylori infection using finger-prick blood, demonstrating high diagnostic performance compared with combined reference standards (13C-urea breath test, immunofluorescence, and immunoblotting), with 93.7% sensitivity, 97.9% specificity, and 97.4% accuracy, supporting its potential use for home-based and large-scale screening.

**Bone and Joint Infections**

- [Tande AJ, Currier BL, Osmon DR. Spinal epidural abscess. N Engl J Med. 2026 Apr 22;394:1621-1633. doi:10.1056/NEJMra2412728](https://ad-id.co/4tK9XmZ)
  - Summary: Spinal epidural abscess is a serious epidural space infection most commonly caused by Staphylococcus aureus (>50% of cases), typically presenting with back or neck pain with fever and/or neurologic deficits, requiring prompt MRI with contrast for diagnosis, microbiologic confirmation via blood or tissue cultures, and urgent multidisciplinary management with spine surgery and infectious disease input, with treatment consisting of surgery and/or targeted antimicrobial therapy depending on patient selection.
- [Beenken KE, Campbell MJ, Reyes-Pardo H, O’Brien CA, Smeltzer MS. Mechanistic insights into the pathogenesis and therapeutic recalcitrance of *Staphylococcus aureus* osteomyelitis. Clin Microbiol Rev. 2026 Apr 16; doi:10.1128/cmr.00259-25](https://ad-id.co/4tAZxpA)
  - Summary: This review summarizes osteomyelitis as a bone infection most commonly caused by Staphylococcus aureus, highlighting its ability to produce severe and treatment-refractory disease through mechanisms such as cortical bone destruction with impaired vascularity, biofilm formation, intracellular persistence in osteoblasts/osteoclasts/osteocytes, formation of small-colony variants and persister cells, and invasion of the osteocyte lacuno-canalicular network, all of which contribute to its dominance as a major orthopedic pathogen and its resistance to conventional antibiotic therapy.

**Bacteraemia and endocarditis**

- [Guideline working group of Chinese Society of Critical Care. Practice guideline on the prevention and treatment of central line-associated bloodstream infection: Part 1––diagnosis and prevention. J Intensive Med. 2026 Apr;6(2):83-92. doi:10.1016/j.jointm.2025.10.008](https://ad-id.co/4tGnHyS)
  - Summary: Updated guidelines from the Chinese Society of Critical Care Medicine provide evidence-based, GRADE-formulated recommendations for the diagnosis and prevention of central line-associated bloodstream infections in adult ICU patients with central venous catheters, aiming to standardize best practices and reduce infection-related morbidity, mortality, and healthcare burden.

**Sexually transmitted infections**

- [Lisco A, Borgognone A, Vanpouille C, Bricker-Holt K, Callier V, Nason M, et al. Bacterial dysbiosis, cervicovaginal human papillomaviruses and inflammation persist in women living with HIV-1 after a year of antiretroviral treatment. J Infect Dis. 2026 Apr 18;jiag211. doi:10.1093/infdis/jiag211](https://ad-id.co/4ubdRow)
  - Summary: In a longitudinal cohort of antiretroviral-naïve Ugandan women living with HIV-1, initiation of ART did not restore cervicovaginal microbiome health, reduce local inflammation, or decrease HPV prevalence over one year, with persistent Gardnerella-dominant dysbiosis and stable high- and low-risk HPV infections despite systemic viral suppression.
- [Logan N, Birhane MG, McDonald SL, Alarcón J, Amador S, Anand M, et al. Emergence of extensively drug-resistant shigellosis — United States, 2011–2023. MMWR Morb Mortal Wkly Rep. 2026 Apr 9;75(13):173–178. doi:10.15585/mmwr.mm7513a1](https://ad-id.co/49licgW)
  - Summary: Extensively drug-resistant (XDR) Shigella infections, defined by resistance to ampicillin, azithromycin, ceftriaxone, ciprofloxacin, and trimethoprim-sulfamethoxazole, have emerged as a growing public health threat in the United States with no FDA-approved oral treatment options, driven by increasing transmission of resistant strains that can also spread resistance genes to other enteric bacteria; analysis of nearly 17,000 isolates from 2011–2023 showed a rise in XDR prevalence from 0% to 8.5% in 2023, with most cases occurring in adult men, a high proportion of Shigella sonnei and Shigella flexneri isolates, frequent absence of travel exposure, and a substantial burden of hospitalization and HIV co-infection, underscoring the need for strengthened surveillance, susceptibility testing, and rapid public health response to control ongoing transmission.

**CNS Infections**

- [Duerlund LS, Larsen L, Storgaard M, Mens H, Wiese L, Grønholm Jepsen MP, et al. Early versus late diagnosis in infectious encephalitis: a population-based cohort study. Clin Microbiol Infect. 2026 Apr 16; doi:10.1016/j.cmi.2026.04.009](https://ad-id.co/4ueyhgw)
  - Summary: In a nationwide Danish cohort of 495 adults with infectious encephalitis, 63% experienced delayed diagnosis (>6 hours after admission), which was more common in older patients and those without altered mental status, and while overall late diagnosis was not independently associated with worse 6-month functional outcome, very delayed diagnosis (>24 hours) was linked to significantly increased 30-day and 6-month mortality.
- [GBD 2023 Meningitis & Antimicrobial Resistance Collaborators. Global, regional, and national burden of meningitis, its risk factors, and aetiologies, 1990–2023: a systematic analysis for the Global Burden of Disease Study 2023. Lancet Neurol. 2026 May;25(5):451-468. doi:10.1016/S1474-4422(26)00101-8](https://ad-id.co/421t1AX)
  - Summary: A Global Burden of Diseases 2023 analysis estimated that meningitis caused 259,000 deaths and 2.54 million cases worldwide in 2023, with children under 5 years bearing a disproportionate burden, and identified Streptococcus pneumoniae, Neisseria meningitidis, and non-polio enteroviruses as leading causes, highlighting that despite declines since 1990, substantial disease burden persists and additional progress toward WHO 2030 targets will require strengthened vaccination, surveillance, and health system interventions, particularly in high-burden regions.

**Respiratory Tract Infections**

- [White AT, Goetz MB, Jones M, Neuhauser MM, Ciarkowski C, Ratz D, et al. Electronic clinical quality measures of antibiotic use in hospitalized adults with uncomplicated community-acquired pneumonia. Clin Infect Dis. 2026 Apr 21;ciag273. doi:10.1093/cid/ciag273](https://ad-id.co/48rSVBy)
  - Summary: Two electronically derived clinical quality measures developed for hospitalized patients with uncomplicated community-acquired pneumonia demonstrated high reliability, validity, and feasibility in large multi-center datasets, revealing that unsupported antibiotic durations beyond 5 days occurred in 40–55% of cases and unnecessary broad-spectrum empiric coverage for MRSA and/or Pseudomonas aeruginosa occurred in 17–56% of cases, supporting their potential use to benchmark and improve antibiotic stewardship practices.
- [Savage TJ, Butler AM, Kronman MP, Durkin MJ, Kattinakere Sreedhara S, Kabbani S, et al. Amoxicillin-clavulanate vs amoxicillin for acute sinusitis in adults. JAMA. 2026 Apr 18; doi:10.1001/jama.2025.26902](https://ad-id.co/4up2R7n)
  - Summary: In a large retrospective cohort of 521,244 adults with uncomplicated acute sinusitis, standard-dose amoxicillin-clavulanate showed no difference in treatment failure compared with amoxicillin (3.0% vs 3.1%), but was associated with a higher risk of secondary infections including yeast infections and Clostridioides difficile infection, supporting standard-dose amoxicillin as a preferred first-line empiric therapy in adults under 65 years when antibiotics are indicated.
- [Deshpande A, Schulte R, Walker RE, Pallotta AM, Wang M, Kadri SS, et al. Impact of procalcitonin testing on antibiotic use in patients hospitalized with community-acquired pneumonia: a multi-hospital cohort study. Clin Infect Dis. 2026 Apr 15;ciag165. doi:10.1093/cid/ciag165](https://ad-id.co/4tDXNMf)
  - Summary: In a multicentre retrospective cohort of 17,290 non-ICU patients hospitalized with community-acquired pneumonia, procalcitonin testing within 24 hours of admission was associated with a modest reduction in antibiotic duration—particularly in patients with low procalcitonin levels—while showing no significant effect on early de-escalation of extended-spectrum antibiotics, suggesting limited but measurable real-world impact on antibiotic stewardship.
  - **Editorial Commentary:**
    [Politis PA, File TM Jr. Use of procalcitonin as a tool to enhance antimicrobial stewardship. Clin Infect Dis. 2026 Apr 15;ciag166. doi:10.1093/cid/ciag166](https://ad-id.co/424RPbe)
- [Hao SY, Lin HY, Yeh PI, Hsiao HC, Lin CY, Huang WH, et al. Multidrug-resistant *Acinetobacter baumannii* pneumonia in clinical therapy: a systematic review and network meta-analysis on effectiveness and safety of different antibiotic combination regimens. Int J Antimicrob Agents. 2026 Apr 21;107815. doi:10.1016/j.ijantimicag.2026.107815](https://ad-id.co/4tD1yBH)
  - Summary: A network meta-analysis of 17 cohort studies (1,420 patients) evaluating treatment regimens for multidrug-resistant Acinetobacter baumannii pneumonia found that colistin-based inhalation therapy ranked highest for clinical response, sulbactam-based regimens showed comparable effectiveness to colistin for clinical and microbiological outcomes, tigecycline-based regimens performed less favorably, and no significant differences in mortality or nephrotoxicity were observed across most regimens except a possible higher mortality signal with colistin–sulbactam combinations, supporting sulbactam-based options as viable alternatives where newer agents are limited.

**Infections in Children**

- [Ramachandran V, Okaro JO, Prasad N, Park S, McGee L, Chochua S, et al. Epidemiology of pediatric invasive group A streptococcal infections in 10 U.S. states, 2004–2023. Clin Infect Dis. 2026 Apr 27;ciag284. doi:10.1093/cid/ciag284](https://ad-id.co/3QTZzua)
  - Summary: In a 2004–2023 US Active Bacterial Core surveillance study of 2,856 pediatric invasive group A Streptococcus cases, incidence remained stable pre–COVID-19, declined during the pandemic, and then rebounded sharply to more than double baseline levels in 2023, with increasing ICU admissions but stable mortality, no penicillin resistance, and a rising trend in clindamycin non-susceptibility alongside shifts in dominant emm1 strains, underscoring the importance of continued surveillance and vaccine development.
- [Mand N, Lieftüchter V, Westhoff JH, Hort S, Biedermann R, Müller-Brandes C, et al. Pediatric intensive care core outcomes–a modified Delphi consensus process (PIC-CO). Crit Care. 2026 Mar 9;30:160. doi:10.1186/s13054-026-05878-1](https://ad-id.co/421jMke)
  - Summary: A Delphi consensus study in German-speaking pediatric intensive care experts developed the first standardized Pediatric Intensive Care Core Outcome (PIC-CO) set, comprising seven validated questionnaires across six domains and 11 short-term outcomes with a structured follow-up schedule, designed to harmonize long-term outcome assessment of PICU survivors in both clinical practice and research, and showing substantial alignment with international efforts while differing mainly in cognitive and domain structuring approaches.

**Miscellaneous Bacterial Infections**

- [Lee N, Tanaka T, Koizumi N, Coughlan C, Sayo AR, Ariyoshi K, et al. Leptospirosis—clinical review and updates on therapeutics. Lancet Infect Dis. 2026 Apr 17; doi:10.1016/S1473-3099(26)00060-5](https://ad-id.co/3QSk5LK)
  - Summary: Leptospirosis is a globally prevalent but underdiagnosed zoonotic infection that disproportionately affects low- and middle-income tropical regions, where outbreaks are driven by environmental and infrastructural factors, and this state-of-the-art review highlights persistent gaps in diagnostics, weak and methodologically limited evidence for antimicrobial therapy, and the need for improved clinical recognition, supportive care, and coordinated research efforts to guide future management strategies.

**Mycobacterial Infections**

- [Barreto Duarte B, Ellner J, Wright AD, Hatherill M, Gupta A, Sterling TR, et al. Asymptomatic tuberculosis across the disease spectrum: a scoping review of epidemiology, diagnosis, and public health implications. Clin Infect Dis. 2026 Apr 15;82(Suppl 2):S75-S84. doi:10.1093/cid/ciag150](https://ad-id.co/3QEYVRf)
  - Summary: A scoping review of asymptomatic tuberculosis highlights that up to 30–50% of bacteriologically confirmed TB cases may present without symptoms yet still harbor active disease and potential transmissibility, with 20–30% progressing to symptomatic TB within two years, underscoring major limitations of symptom-based screening and the need for improved diagnostic tools, including radiographic and biomarker-based approaches, to better detect and control this hidden but epidemiologically important stage of TB.
- [Araújo-Pereira M, Bhavaraju R, Hatherill M, Gupta A, Ellner JJ, Sterling TR, et al. From Exposure to Disease: Predicting Tuberculosis Progression Free. Clinical Infectious Diseases. 2026 Apr 15;82(Suppl_2):S68–S74. doi:10.1093/cid/ciag125](https://ad-id.co/48vND7Y)
  - Summary: Mycobacterium tuberculosis infects approximately one-quarter of the global population, yet only 5–10% progress to active tuberculosis, and this review synthesizes evidence from the RePORT International consortium on transmission dynamics, host and genetic biomarkers, and predictive models for identifying high-risk individuals—particularly close contacts of TB cases—highlighting both the promise and limitations of current approaches and emphasizing the need for improved, context-specific tools to enable targeted prevention strategies and advance tuberculosis elimination efforts.
- [Gupte AN, Boisson-Walsh A, Huaman MA, Bhavaraju R, Gao Y, Hom D, et al. Long-term Sequelae of Pulmonary Tuberculosis: A Narrative Review. Clinical Infectious Diseases. 2026 Apr 15;82(Suppl_2):S60–S67. doi:10.1093/cid/ciag123](https://ad-id.co/4uluyhj)
  - Summary: Tuberculosis (TB) survivors experience substantial long-term morbidity and mortality despite successful treatment, including frequent persistent ventilatory defects (affecting over half of patients), impaired lung diffusion capacity, airway and parenchymal damage, and common complications such as bronchiectasis and chronic pulmonary aspergillosis in about one-third of cases, alongside increased risks of cardiovascular disease (including myocardial infarction, stroke, and peripheral arterial disease) and lung cancer independent of smoking, while current evidence suggests that dysregulated host inflammation contributes to these sequelae but major gaps remain in identifying high-risk individuals, defining disease phenotypes and trajectories, and developing prognostic biomarkers and targeted immunomodulatory interventions.
- [Smith JP, O’Connor S, Date A, Moonan PK. Tuberculosis Cases and Deaths Averted by PEPFAR. N Engl J Med. 2026 Mar 24;394(16):1655–1656. doi:10.1056/NEJMc2506284](https://ad-id.co/4tSoMUv)
  - Summary: Using a country-level negative binomial modelling framework, this study estimates that the U.S. President’s Emergency Plan for AIDS Relief (PEPFAR), through both indirect effects of HIV epidemic control and expanded direct tuberculosis interventions (including symptom screening, diagnostic scale-up, and preventive therapy), averted approximately 11.0 million tuberculosis cases and 2.1 million tuberculosis-related deaths among people living with HIV from 2003–2024, with a growing contribution from direct TB-specific measures in recent years, highlighting the substantial and increasing role of integrated HIV–TB programming in global tuberculosis control and elimination efforts.
- [Wilber E, Schechter MC, Rebolledo PA, Kandiah S, Aldred B, Wang YF, et al. Real-World Outcomes of Chemiluminescence Immunoassay Implementation for Mycobacterium tuberculosis Infection Screening. Open Forum Infectious Diseases. 2026 Apr 22;ofag234. doi:10.1093/ofid/ofag234](https://ad-id.co/48wc5Gk)
  - Summary: The implementation of a fully automated CLIA-based interferon-gamma release assay (IGRA) for tuberculosis infection testing was associated with a substantial increase in borderline-positive results (87.8% relative increase, 95% CI 31.1–170.0%) compared with semi-automated assays, and this rise in equivocal positivity rates subsequently led to changes in clinical management pathways, raising concerns about potential impacts on test interpretation and downstream TB infection care.
- [Zhang H, Medina-Jaudes N, Forcada-Nadal A, Harrison EM, Coll F. In Host Mutational Adaptation of Mycobacterium Tuberculosis Complex Strains During Tuberculosis Infection. J Infect Dis. 2026 Apr 28;jiag209. doi:10.1093/infdis/jiag209](https://ad-id.co/425gtZm)
  - Summary: A meta-analysis of 5,882 Mycobacterium tuberculosis genomes from 1,044 patients using a within-host convergent evolution approach identified 21 genes, 25 operons, and 27 promoter regions significantly enriched for mutations, revealing both known and novel bacterial adaptations mainly associated with drug resistance across first-, second-, and last-line TB therapies as well as virulence pathways including the ESX-1 locus, and further demonstrating that fluoroquinolone resistance is acquired more frequently during treatment than resistance to other anti-TB drugs, highlighting key evolutionary mechanisms underlying treatment failure and TB pathogenesis.
- [Chen R, Li B. Mind the Postpartum Trough: Closing the Clofazimine Exposure Gap After Delivery in Rifampicin-Resistant Tuberculosis. J Infect Dis. 2026 Apr 23;jiag230. doi:10.1093/infdis/jiag230](https://ad-id.co/48wlFcn)
  - Summary: Hughes and colleagues report intensive pharmacokinetic data from 11 pregnant and postpartum women treated with clofazimine for rifampicin-resistant tuberculosis in South Africa, showing that third-trimester drug exposure (AUClast and Cmax) was approximately 30% higher than in the postpartum period, with postpartum Cmax only modestly exceeding the commonly cited bactericidal threshold (0.25 µg/mL) even after prolonged therapy, and with one-third of postpartum women falling below lower historical exposure benchmarks; these findings highlight a potential postpartum pharmacokinetic “dip” that may represent a clinically important window of vulnerability for suboptimal tuberculosis treatment exposure, potentially influenced by physiological changes, drug–drug interactions (e.g., CYP3A4 and P-glycoprotein modulation by antiretrovirals such as lopinavir/ritonavir), and unmeasured adherence factors.
- [Jones-López EC, Miller NS, Orr B, White LF, Vinhas S, Mpeirwe M, et al. Unexpected detection of Mycobacterium tuberculosis DNA in US-born patients in putative association with clinical syndromes. Nat Commun. 2026 Apr 14;17:2709. doi:10.1038/s41467-026-70890-6](https://ad-id.co/4ujF6xo)
  - Summary: In early development work evaluating an ultrasensitive molecular assay for tuberculosis detection, three clinical studies involving 297 respiratory samples from hospitalized patients in Boston unexpectedly identified a high prevalence of Mycobacterium tuberculosis DNA (confirmed by alternative molecular targets and sequencing) even among US-born individuals, along with a potential association between TB-DNA detection and acute chest syndrome in patients with sickle cell disease, highlighting possible undetected paucibacillary or subclinical TB signals in low-incidence settings and underscoring the need for prospective studies with integrated clinical, radiological, immunological, and microbiological correlation to clarify the significance of these findings.
- [Chancharoenthana W, Siripoon T, Kamolratanakul S, Luvira V, Phumratanaprapin W, Schultz MJ, et al. Management of latent tuberculosis infection in patients with kidney disease. Clin Microbiol Rev. 2026 Apr 20; doi:10.1128/cmr.00353-25](https://ad-id.co/42pBXjT)
  - Summary: This narrative review synthesizes evidence on latent tuberculosis infection (LTBI) management in patients with chronic kidney disease across pre-dialysis, dialysis, and kidney transplant populations, highlighting that immunosuppression and uremia increase TB reactivation risk, that interferon-gamma release assays are preferred over tuberculin skin testing in advanced CKD, and that screening should be risk-based in pre-dialysis patients, systematic at dialysis initiation, and mandatory before transplantation; it further recommends exclusion of active TB prior to preventive therapy and supports short-course rifamycin-based regimens (4R, 3HP, 3HR) for improved adherence in most CKD patients, while favoring 9 months of isoniazid in transplant recipients due to drug–drug interactions with immunosuppressants, emphasizing structured monitoring, pyridoxine use, and tailored implementation strategies to reduce progression to active TB and improve renal and transplant outcomes.
- [Larsson L, Olaru ID, Behnke AL, Marambire ET, Chipinduro M, Calderwood CJ, et al. Diagnostic and prognostic accuracy of the Mycobacterium tuberculosis host response 3-gene cartridge among tuberculosis household contacts in Mozambique, Tanzania, and Zimbabwe: a prospective, longitudinal, diagnostic and prognostic accuracy cohort study. Lancet Infect Dis. 2026 Apr 20; doi:10.1016/S1473-3099(26)00114-3](https://ad-id.co/48vb1T7)
  - Summary: In a prospective longitudinal study of 2,079 household contacts of tuberculosis patients in Mozambique, Tanzania, and Zimbabwe, a host-response assay (Cepheid MTB-HR) demonstrated good diagnostic accuracy for active tuberculosis (AUROC 0.86) when tested within 30 days of disease confirmation, but showed more limited and declining prognostic performance for predicting incident TB over time (AUROC 0.80 at 1–6 months, 0.64 at 6–12 months, and 0.71 at 1–12 months), with low positive predictive value (~7.5% at 6 months), indicating that while host-response assays may be useful for detecting prevalent disease in high-risk contacts, their ability to reliably predict future tuberculosis development remains limited.
- [Stein CM, Benchek P, Mantshoyane L, Ciesielski T, McHenry ML, Wilson-Sesay H, et al. Human Genetic Variation Associates With Infection by Derived Ugandan *Mycobacterium tuberculosis* Lineage. J Infect Dis. 2026 Apr 18;jiag218. doi:10.1093/infdis/jiag218](https://ad-id.co/49hhP72)
  - Summary: In a genome-wide association study of 276 culture-confirmed tuberculosis cases from a Ugandan household contact cohort, investigators examined human genetic variants in relation to infection with the geographically restricted M. tuberculosis L4-Uganda sub-lineage versus other L4 lineages and found multiple loci with suggestive associations supported by convergent functional evidence (including gene expression in immune and lung tissues, eQTL signals, signatures of natural selection, and CRISPR immune screens), alongside replication of previously reported host–pathogen interaction effects, providing further evidence that host genetic susceptibility to tuberculosis is shaped by strain-specific host–pathogen co-evolution involving immune-relevant pathways.
- [Ghanem M, Srivastava R, Ektefaie Y, Hoppes D, Rosenfeld G, Yaniv Z, et al. Tuberculosis Disease Severity Assessment Using Clinical Variables and Radiology Enabled by Artificial Intelligence. J Infect Dis. 2026 Apr 16;jiag149. doi:10.1093/infdis/jiag149](https://ad-id.co/4uhWv9J)
  - Summary: In an analysis of 2,809 patients from the TB Portals real-world dataset, chest X-ray–derived percent of lung involved (PLI) measured by human readers was identified as the most predictive radiological marker of unfavorable tuberculosis treatment outcomes across drug resistance and HIV subgroups, outperforming cavitation and commercially available AI-derived imaging features, and providing additional predictive value when combined with age, sex, and smear grade; furthermore, convolutional neural network models could accurately automate PLI estimation from imaging data (AUC 0.85 for PLI >25%), supporting scalable integration of radiological severity scoring into TB risk stratification and outcome prediction.
- [Yerlikaya S, Chirwa M, Ajide B, Castro MDM, Ha H, Kato-Maeda M, et al. Pulmonary Tuberculosis Detection with MiniDock MTB Using Swab Samples. N Engl J Med. 2026 Apr 29;394(17):1710–1722. doi:10.1056/NEJMoa2509761](https://ad-id.co/4u0LAlf)
  - Summary: In a multicountry prospective diagnostic accuracy study across seven high-burden settings, the MiniDock MTB point-of-care test using sputum and tongue swabs demonstrated high sensitivity (85.7% and 79.6%, respectively) and high specificity (>97.5%) for pulmonary tuberculosis detection compared with sputum culture, performed comparably to Xpert MTB/RIF Ultra, exceeded smear microscopy sensitivity, and met WHO target product profile criteria while showing good usability (median system usability score 75), supporting its potential role as a scalable near–point-of-care TB diagnostic in peripheral health centers.

**Fungal Infections and antifungal agents**

- [Gómez Londoño LF, Oliveira Souza AC, Burt B, Das S, Ge W, Meza-Perez V, et al. Prevalence of cyp51A variants in a US collection of *Aspergillus fumigatus* clinical isolates and comparative analysis of their contribution to triazole antifungal resistance. J Antimicrob Chemother. 2026 May;81(5):dkag124. doi:10.1093/jac/dkag124](https://ad-id.co/4tKfwBS)
  - Summary: In a genomic and functional analysis of 87 triazole-resistant Aspergillus fumigatus clinical isolates from the USA collected between 2007 and 2020, researchers identified 15 cyp51A gene variants accounting for reduced susceptibility to mould-active triazole antifungals in 60% of isolates—most commonly TR34/L98H and TR46/Y121F/T289A—affecting resistance to short-, long-, and multiple-chain triazoles, while 16% of isolates showed reduced susceptibility without mutations in known resistance genes (cyp51A, hmg1, or hapE), highlighting substantial genetic diversity in antifungal resistance mechanisms and underscoring the need for further discovery of unexplained resistance pathways.
- [Bing J, Li S, Ji L, Du H, Shamoon NM, Nobile CJ, et al. Global emergence and rapid spread of *Candidozyma auris* (syn. *Candida auris*): epidemiology, biology, and antifungal resistance. Clin Microbiol Rev. 2026 Apr 22; doi:10.1128/cmr.00394-25](https://ad-id.co/3QWkpsV)
  - Summary: This review summarizes the global emergence and rapidly expanding epidemiology of the multidrug-resistant fungal pathogen Candidozyma auris (syn. Candida auris), which since its first identification in Japan in 2009 has spread to 82 countries across six continents with 84,941 reported colonization or infection cases as of December 2025, noting a marked post–COVID-19 pandemic acceleration; it highlights key biological and genetic features underlying its environmental persistence, transmissibility, antifungal resistance, and virulence, and discusses evolving global transmission patterns alongside strategies needed to mitigate further spread of this high-consequence healthcare-associated pathogen.
- [Chorafa E, Iosifidis E, Tzika C, Papathanasiou AI, Roilides E. Antifungal Stewardship in the Pediatric Intensive Care Unit: Time is of the Essence. Infect Drug Resist. 2026 Apr 24;19:541588. doi:10.2147/IDR.S541588](https://ad-id.co/4tLnQRU)
  - Summary: Invasive fungal infections in pediatric intensive care units remain a major cause of morbidity and mortality, with rising proportions of non-albicans Candida species and emerging antifungal resistance, while antifungal use is still largely empiric rather than diagnostic-driven despite available fungal biomarkers; this review highlights limited evidence and lack of clear guidance for antifungal prophylaxis outside traditional high-risk groups, identifies gaps in antifungal stewardship implementation in PICUs (with only nine reported interventions, mostly embedded in broader hospital programs and relying on audit-and-feedback strategies), and emphasizes the need for structured antifungal stewardship frameworks focused on improving diagnostic-driven therapy, appropriate prophylaxis selection, and therapeutic drug monitoring to optimize antifungal use in critically ill children.
- [Cohen SH, Allyn P, Chow FC, Rathe J, Nanayakkara DD, Hutfles G, et al. Fosmanogepix treatment for coccidioidomycosis under an expanded access program. Clin Infect Dis. 2026 Apr 21;ciag271. doi:10.1093/cid/ciag271](https://ad-id.co/4tJ3OHu)
  - Summary: In an expanded-access program (NCT06433128), the novel antifungal fosmanogepix demonstrated promising efficacy and tolerability in severe coccidioidomycosis, achieving clinical success in 8 of 11 patients—including 75% (6/8) of those with meningitis and 66% (2/3) with non-meningeal disseminated disease—with a median treatment duration of 208 days, suggesting potential utility for difficult-to-treat invasive fungal infections.
- [Monforte A, Los-Arcos I, Martín-Gómez MT, Berastegui C, Márquez-Algaba E, Sacanell J, et al. Breakthrough Mold Infections During Long-term Nebulized Liposomal Amphotericin B Prophylaxis in Lung Transplant Recipients: A Single-center Cohort (NEBULA Study). Transplantation. 2026 May;110(5):e1141–e1151. doi:10.1097/TP.0000000000005686](https://ad-id.co/49m0PfZ)
  - Summary: Invasive fungal infections in pediatric intensive care units and transplant populations remain a major cause of morbidity and mortality, driven by rising non-albicans Candida species, emerging antifungal resistance (including multidrug-resistant Candida auris with global spread exceeding 84,000 cases across 82 countries by 2025), and high-risk settings such as lung transplantation where breakthrough mold infections persist despite prophylaxis; evidence highlights challenges in diagnosis and stewardship, limited use of diagnostic-driven antifungal strategies and prophylaxis guidance in PICUs, and the need for antifungal stewardship interventions alongside improved prevention approaches such as lifelong nebulized liposomal amphotericin B in lung transplant recipients, novel agents like fosmanogepix showing encouraging outcomes in invasive coccidioidomycosis, and ongoing surveillance of azole resistance mechanisms in Aspergillus fumigatus.
- [Buil JB, Sterenborg RBTM, Verweij PE, Oude Elferink P, van der Heijden WA, van de Veerdonk FL, et al. Funguria with severe complications in diabetic patients receiving SGLT2 inhibitors. Clin Microbiol Infect. 2026 Apr 25; doi:10.1016/j.cmi.2026.04.019](https://ad-id.co/4tTiow4)
  - Summary: Sodium-glucose cotransporter-2 inhibitors (SGLT2i), including canagliflozin, dapagliflozin, and empagliflozin, are widely used in type 2 diabetes and induce glycosuria that alters the urinary microenvironment, potentially increasing susceptibility to yeast colonization and urinary tract infections; while candiduria has traditionally been considered benign and often not requiring antifungal treatment except in high-risk settings such as neutropenia or urological procedures, emerging evidence suggests that the increasing use of SGLT2i may shift this paradigm by raising clinical concern for more complicated fungal urinary tract disease, including pyelonephritis, obstructive fungal balls, and candidemia.

**Virulence**

- [Schaufler K, Schmidt N, Schwabe M, Heiden SE, Fickenscher H, Woh PY, et al. Rethinking virulence screening in *Klebsiella pneumoniae*: a case for a standardised *Galleria mellonella* infection model. Lancet Microbe. 2026 Apr 28;101421. doi:10.1016/j.lanmic.2026.101421](https://ad-id.co/3QJhQdG)
  - Summary: Genome-based Klebsiella pneumoniae classification tools such as Kleborate enable rapid assignment of isolates into virulence and antimicrobial resistance–defined pathotypes, but phenotypic validation has been limited by the scalability and ethical constraints of murine models, prompting interest in the Galleria mellonella larval infection model as a mid-throughput alternative; in this study, a rigorously standardised G. mellonella workflow applied to 80 genomically defined isolates demonstrated reproducible survival differences that clearly distinguished classic low-virulence strains (regardless of resistance status) from hypervirulent and convergent high-virulence/high-resistance strains, supporting the model’s utility in phenotypically validating major Kleborate-defined pathotypes across phylogenetically diverse backgrounds.

**Host Factors in Infection**

- [Kottom TJ, Pellegrino M, Achilonu C, Stelzig KE, Limper AH. The tyrosine kinase receptor EphA2 in alveolar macrophages provides a protective role in host defense against Pneumocystis pneumonia. J Infect Dis. 2026 Apr 23;jiag228. doi:10.1093/infdis/jiag228](https://ad-id.co/49jEQ9r)
  - Summary: EphA2, a β-glucan–binding receptor on alveolar macrophages, regulates host immune responses during Pneumocystis pneumonia by promoting proinflammatory cytokine production that supports pathogen clearance but also drives lung inflammation; in a CD4-depleted mouse model, EphA2 deficiency or pharmacologic inhibition reduced pulmonary inflammation but increased Pneumocystis burden in untreated infection, while EphA2 inhibition during antimicrobial therapy attenuated lung inflammatory responses, suggesting that EphA2 signaling is a key determinant of the balance between antimicrobial defense and immunopathology and may represent a potential therapeutic target to modulate inflammation in severe Pneumocystis pneumonia.

**Diagnostics**

- [Gador-Whyte AP, Sherry NL, Brischetto A, Andersson P, Bond KA, van Hal SJ, et al. Implementation of pathogen genomics in clinical microbiology laboratories. Clin Microbiol Rev. 2026 Apr 27; doi:10.1128/cmr.00177-25](https://ad-id.co/4ujwnLt)
  - Summary: Pathogen genomics, including whole-genome sequencing and clinical metagenomics, is increasingly being adopted in clinical microbiology to improve rapid infection diagnosis, enhance healthcare-associated infection control, and potentially replace traditional microbiology workflows, but real-world implementation in hospital laboratories remains limited compared with public health settings due to barriers such as funding constraints and shortages of genomics-trained personnel; this review outlines key clinical use cases and implementation models, proposes a framework for laboratories adopting sequencing, and highlights future opportunities including direct-from-specimen sequencing, machine learning–enabled genomic analysis, and integration of pathogen genomics into clinical decision support systems.
- [Glover JS, Humphries R. Novel Rapid Phenotypic Susceptibility Testing Techniques That Enhance Antimicrobial Stewardship and Clinical Decision-Making. Clin Chem. 2026 Apr 24;hvag033. doi:10.1093/clinchem/hvag033](https://ad-id.co/4ubOmDM)
  - Summary: Antimicrobial resistance continues to drive the need for faster diagnostic approaches that enable timely and targeted therapy, and rapid phenotypic antimicrobial susceptibility testing (AST) has emerged as a key innovation by providing earlier susceptibility results than conventional methods, particularly in severe infections such as sepsis where delays in effective antibiotics worsen outcomes; although promising in improving antimicrobial stewardship and reducing time to appropriate therapy, challenges remain including variability in test performance, lack of standardised protocols, and cost-effectiveness considerations, and this review highlights emerging growth-based phenotypic AST technologies and their clinical and operational implications as a pivotal step toward improving evidence-based antimicrobial management.
- [Langford BJ, Leung E, Jeong E, Wong J, Scherer A, Matukas LM, et al. Nudging in MicroBiology Laboratory Evaluation (NiMBLE): a systematic review and meta-analysis. Clin Microbiol Infect. 2026 Apr 17; doi:10.1016/j.cmi.2026.04.008](https://ad-id.co/4tHMWkn)
  - Summary: In response to rising antimicrobial resistance, clinical microbiology laboratories play a central role in antimicrobial stewardship by providing timely and reliable antimicrobial susceptibility testing (AST) results that guide appropriate prescribing decisions such as initiation, de-escalation, escalation, or discontinuation of antibiotics; within this context, selective or cascade reporting of susceptibility results has emerged as a behavioural “nudge” intervention that subtly modifies clinicians’ choice architecture without removing autonomy, aiming to improve prescribing behaviour, though current guideline support remains weak due to limited high-quality evidence; building on earlier observational and heterogeneous studies suggesting potential benefits, this systematic review and meta-analysis evaluates the impact of microbiology report nudging strategies on antimicrobial utilisation and prescribing appropriateness as well as associated clinical outcomes.
- [Hommel B. Beyond sensitivity and specificity: designing outcome-driven evidence for infectious disease in vitro diagnostics. Clin Microbiol Infect. 2026 Apr 22; doi:10.1016/j.cmi.2026.04.016](https://ad-id.co/4ul3z5r)
  - Summary: Diagnostic tests are traditionally evaluated using sensitivity and specificity, but these performance metrics often fail to reflect their real-world clinical impact, creating a gap that is increasingly important for the in vitro diagnostics (IVD) industry where reimbursement, regulatory approval, and market adoption depend on demonstrating patient and health system value; in the current landscape of outcome-focused payer expectations and growing acceptance of real-world evidence by regulators, emphasis is shifting from purely analytical accuracy toward whether diagnostic tests meaningfully improve clinical outcomes, and this commentary aims to help clinicians, microbiologists, and researchers interpret diagnostic evidence beyond accuracy metrics while recognising the practical constraints in generating robust outcome-based evidence for IVDs.
- [Lamb G, Pallett SJCP, Charani E, Kanu EM, Alqahtani SA, Villegas MV, et al. Assessment of the Prioritization of Diagnostics in National Action Plans for Antimicrobial Resistance. Clin Infect Dis. 2026 Apr 29;ciag020. doi:10.1093/cid/ciag020](https://ad-id.co/4tN7bgI)
  - Summary: In a targeted analysis of 142 World Health Organization member state national action plans (NAPs) for antimicrobial resistance, diagnostics were included in the vast majority (95.8%), primarily focused on surveillance and laboratory capacity strengthening, but point-of-care and molecular diagnostics were underrepresented (each 33.1%, with lower uptake in low- and middle-income countries), and only a minority of NAPs (24.6%) included diagnostic-specific key performance indicators—highlighting substantial gaps in implementation planning and evaluation frameworks needed to translate AMR diagnostic strategies into measurable real-world impact.
  - **Editorial Commentary:**
    [Mo Y. Transforming Antimicrobial Resistance Diagnostics From Conceptual Priority to Operational Reality. Clin Infect Dis. 2026 Apr 29;ciag021. doi:10.1093/cid/ciag021](https://ad-id.co/4tLnkDo)

**Improving Clinical Research**

- [Normand SLT. Interpreting Evidence with Bayesian Meta-Analysis. NEJM Evid. 2026 Apr 15;5(5). doi:10.1056/EVIDe2600097](https://ad-id.co/48yemAZ)
  - Summary: This commentary discusses a Bayesian meta-analysis of 32 trials (nearly 28,000 ICU patients) evaluating selective decontamination of the digestive tract in mechanically ventilated adults, which found an association with reduced in-hospital mortality (risk ratio 0.91, 95% credible interval 0.82–0.99), while highlighting methodological considerations in Bayesian versus frequentist inference, including interpretation of prior distributions, random-effects modelling assumptions, prediction intervals, heterogeneity across trial designs, and the implications of multiple subgroup analyses without formal multiplicity adjustment; it also emphasizes that despite evidence of mortality reduction, residual confounding and between-trial heterogeneity inherent in meta-analyses limit definitive causal inference, even when using advanced Bayesian statistical frameworks.

**General Interest**

- [Rosseau N, Beatty NL. *Vibrio vulnificus* Necrotizing Soft-Tissue Infection. N Engl J Med. 2026 Apr 22;394:e31. doi:10.1056/NEJMicm2600207](https://ad-id.co/4unpbhG)
  - Summary: A 74-year-old man developed rapidly progressive necrotizing soft-tissue infection after sustaining a leg laceration while swimming in warm Gulf Coast waters, later presenting with contralateral arm involvement and systemic signs including ecchymosis, crepitus, and hemorrhagic bullae; blood and tissue cultures identified Vibrio vulnificus, a marine pathogen found in warm low-salinity coastal environments typically acquired through wound exposure to seawater or ingestion of undercooked seafood, with infection risk expected to increase due to climate change–driven ocean warming and ecological shifts. Despite urgent surgical debridement, above-knee amputation, forearm autografting, and antibiotic therapy with ceftriaxone and doxycycline, the case illustrates the fulminant nature and high morbidity of V. vulnificus necrotizing infection.
- [Green AS, Feldman WB, Solomon DA. Ethical Considerations in the Use of Indwelling Lines in Persons Who Use Injection Drugs. Clin Infect Dis. 2026 Apr 28;ciag109. doi:10.1093/cid/ciag109](https://ad-id.co/3QFFHei)
  - Summary: Clinicians and hospital systems frequently face ethical and clinical uncertainty when considering indwelling intravenous catheters for long-term antibiotic therapy in persons who inject drugs (PWID), due to concerns about potential catheter misuse for non-prescribed drug administration and associated risks of infection, thrombosis, and exacerbation of substance use disorder; this review synthesizes emerging real-world evidence on outcomes of long-term intravenous access in PWID and proposes a case-based ethical framework grounded in principles of beneficence, nonmaleficence, and patient autonomy to guide decision-making, including consideration of when, if ever, forcible line removal may be justified.
- [Cotton D. “Never More Than 15 Feet From the Respirator”: Housestaff Research During the 1955 Boston Polio Epidemic. Ann Intern Med. 2026 Apr 28; doi:10.7326/ANNALS-25-05440](https://ad-id.co/48yTrxA)
  - Summary: This historical account describes how the 1955 Massachusetts polio epidemic, occurring shortly after the Salk vaccine rollout, overwhelmed local health systems and led Massachusetts General Hospital residents and a medical student to manage a rapidly established polio ward and laboratory under crisis conditions, where they conducted patient-oriented research alongside intensive bedside care of patients often supported by iron lungs; enabled by extended work hours, a strong institutional culture of bedside research, and minimal separation between clinical and research roles in an era without formal research consent requirements, this experience shaped subsequent careers and is contrasted with modern restrictions on trainee autonomy during recent pandemic responses such as COVID-19.
- [Zeltina E, Krastina J, Buce-Satoba I, Zelve Z, Pastare D, Ptašekas J, et al. From sore throat to sepsis: unmasking life-threatening Lemierre syndrome. BMJ Case Rep. 2026 Apr;19(4):e269553. doi:10.1136/bcr-2026-269553](https://ad-id.co/4ue7HnD)
  - Summary: Lemierre syndrome is a rare but severe complication of typically oropharyngeal bacterial infection in otherwise healthy young individuals, characterised by progression to septic thrombophlebitis of the internal jugular vein with potential septic embolisation; this case describes a young male who developed sepsis following sore throat and fever, with imaging confirming internal jugular vein thrombosis and blood cultures identifying Fusobacterium necrophorum, and who was subsequently found to have underlying thrombophilia likely contributing to disease severity, with successful treatment achieved through broad-spectrum antibiotics and anticoagulation, underscoring the importance of early recognition and intervention despite declining incidence in the antibiotic era.

**Target Trial Emulation**

- [Aslan AT, Ezure Y, Tanriverdi ES, Dağ O, Cimen C, Kaya Kalem A, et al. Emulating a target trial of early compared with late initiation of appropriate antibiotic therapy for hospital-acquired monobacterial Gram-negative bloodstream infections. J Antimicrob Chemother. 2026 Jun;81(6):dkag161. doi:10.1093/jac/dkag161](https://ad-id.co/4ug6ZXA)
  - Summary: In a multi-centre prospective cohort study of 680 adults with hospital-acquired bloodstream infections caused by Gram-negative bacteria across 22 Turkish hospitals, emulated target trial analysis showed that early appropriate antimicrobial therapy compared with late appropriate therapy was associated with significantly lower 14-day all-cause mortality (14.7% vs 42.9%, aHR 0.41) and 28-day mortality (26.1% vs 49.5%, aHR 0.66), with consistent survival benefits also observed in carbapenem-resistant subgroups, although residual confounding could not be excluded.
- [Li P, Xu S, Chen C, Hu X, Li Y, Wang J, et al. Early antifungal prophylaxis fails to prevent influenza-associated pulmonary aspergillosis in the ICU: a target trial emulation. Clin Microbiol Infect. 2026 May 9; doi:10.1016/j.cmi.2026.05.002](https://ad-id.co/4fhQXru)
  - Summary: In a multicenter target-trial emulation involving 2919 critically ill adults with influenza across 48 ICUs, early antifungal prophylaxis within 24 hours of ICU admission was not associated with a reduction in influenza-associated pulmonary aspergillosis after inverse probability weighting adjustment (HR 0.88), showed benefit only in ICUs with the highest baseline aspergillosis incidence, and was linked to increased hepatotoxicity without improvements in ICU discharge rates, nephrotoxicity, or 28-day mortality, suggesting that routine universal prophylaxis is not supported.

**Antibiotic Therapy Reviews**

- [Pallotta RH, Spivak ES. Use of long-acting lipoglycopeptide antibiotics—beyond convenience. JAMA Intern Med. 2026 Apr 27; doi:10.1001/jamainternmed.2026.0977](https://ad-id.co/49uSFC8)
  - Summary: This review describes how long-acting lipoglycopeptides such as dalbavancin and oritavancin, which are FDA-approved for acute bacterial skin and skin structure infections, are increasingly used off-label for complex gram-positive infections including bacteremia, osteomyelitis, and endocarditis due to their long half-lives and once-weekly dosing, particularly in situations where prolonged intravenous therapy or hospitalisation is challenging, and it summarises their pharmacology alongside emerging clinical evidence supporting these broader applications.

**PK/PD and Drug Dosing**

- [Fresán D, Sorlí L, Barceló-Vidal J, Rodrigo-Moreno A, Muñoz R, Gracia MP, et al. Early adequate linezolid concentrations and clinical outcomes in confirmed Gram-positive infections: the role of therapeutic drug monitoring. J Antimicrob Chemother. 2026 Jun;81(6):dkag137. doi:10.1093/jac/dkag137](https://ad-id.co/491e5GX)
  - Summary: In a retrospective cohort of 400 patients with confirmed Gram-positive infections receiving linezolid therapeutic drug monitoring, only 34% achieved therapeutic trough concentrations (2–8 mg/L) at first measurement, and achieving this target was independently associated with higher clinical cure rates (OR 1.78), while higher comorbidity burden, liver cirrhosis, and ICU-level sepsis were linked to treatment failure, supporting the potential role of early TDM-guided dosing despite no independent association with mortality outcomes.
- [Gatti M, De Paola R, Rinaldi M, Laici C, Siniscalchi A, Viale P, et al. ARC in critical ill OLT recipients: true prevalence, risk factors, and impact on early aggressive PK/PD target non-attainment of CI beta-lactam therapy for posttransplant Gram-negative infections. Transpl Infect Dis. 2026 May 7:e70237. doi:10.1111/tid.70237](https://ad-id.co/4dppvpk)
  - Summary: In a retrospective study of 112 critically ill orthotopic liver transplant recipients, augmented renal clearance (ARC) was found in 23.2% of patients and was associated with younger age, lower disease severity, and fewer intraoperative complications, while standard eGFR equations poorly estimated measured creatinine clearance; importantly, in those receiving continuous-infusion beta-lactams, ARC was the only independent factor linked to early pharmacokinetic/pharmacodynamic target non-attainment, highlighting its clinical impact on antibiotic dosing adequacy in this population.

**Antibiotics - In vitro susceptibility**

- [Karlowsky JA, Estabrook M, Suzuki E, Shimizu A, Okade H. In vitro activity of aztreonam–nacubactam and cefepime–nacubactam against a global collection of clinically important Gram-negative bacilli collected in 2021–2023. JAC Antimicrob Resist. 2026 Jun;8(3):dlag061. doi:10.1093/jacamr/dlag061](https://ad-id.co/3P1acuU)
  - Summary: In a large in vitro surveillance study of 2021–2023 global clinical isolates of Gram-negative bacilli, aztreonam–nacubactam and cefepime–nacubactam demonstrated potent activity against Enterobacterales, including carbapenem-resistant strains (99.8% inhibited at ≤4 and ≤8 mg/L respectively), with substantial MIC reductions even in aztreonam- and cefepime-resistant isolates and retained activity against many isolates resistant to other β-lactam/β-lactamase inhibitor combinations, supporting their continued clinical development for difficult-to-treat Gram-negative infections, although activity was limited against Pseudomonas aeruginosa and poor against Acinetobacter baumannii.

**Beta-Lactamases and Other Resistance Mechanisms**

- [Gu J, Gao L, Chen S, Xu M, Goyal J, Haider R, et al. Deep learning insights into β-lactamase dynamics and resistance evolution. Biochem J. 2026 May 8;483(6):861-879. doi:10.1042/BCJ20260196](https://ad-id.co/3Pr1bvd)
  - Summary: This review discusses how emerging deep learning approaches applied to molecular dynamics simulations are advancing the understanding of β-lactamase evolution and antimicrobial resistance by capturing conformational heterogeneity, identifying metastable states and cryptic binding pockets, and modelling active-site remodeling and allosteric networks, with methods such as convolutional variational autoencoders, supervised DiffNets, and graph neural networks enabling improved prediction of mutational effects, substrate specificity, and inhibitor susceptibility across β-lactamase classes A–D.
- [Ono D, Harada S, Ishii Y, Mojica MF, Bonomo RA. Treatment strategies for imipenemase-producing Gram-negative infections: lessons from Japan. Lancet Microbe. 2026 May 11; doi:10.1016/j.lanmic.2026.101413](https://ad-id.co/494i7hL)
  - Summary: This personal view highlights the global clinical challenge of carbapenem-resistant Enterobacterales driven by diverse carbapenemase families, emphasizing that while new β-lactam/β-lactamase inhibitor combinations and cefiderocol have expanded treatment options, their efficacy varies significantly across serine (KPC, OXA-48) and metallo-β-lactamases (NDM, VIM, IMP), with IMP-producing organisms—particularly prevalent in East Asia and under-surveilled globally—showing distinct biochemical and therapeutic response patterns that remain poorly defined, underscoring the need for tailored, regionally informed treatment and surveillance strategies.
- [David S, Kohlenberg A, Yeats C, Abu-Dahab K, Albiger B, Alikhan NF, et al. Emergence of carbapenemase-producing Escherichia coli in acute care hospitals in 32 European countries (the CCRE survey): a prospective, multicentre, cross-sectional, epidemiological, microbiological, and genomic surveillance study. Lancet Microbe. 2026 May 13; doi:10.1016/j.lanmic.2025.101321](https://ad-id.co/4eOoKZe)
  - Summary: In a large 2019 European multicountry genomic surveillance study of 548 Escherichia coli isolates from 32 countries, carbapenem-resistant or reduced-susceptibility strains were frequently associated with high-risk sequence types such as ST131, ST410, ST38, ST167, and ST648, with 86.3% carrying carbapenemase genes—most commonly blaNDM-5 and blaOXA-48—with a marked rise in blaNDM-5 compared to earlier surveys, while phylogenetic analysis indicated multiple international introductions but limited onward transmission within Europe, highlighting the ongoing global spread of carbapenem-resistant E. coli and the need for strengthened genomic surveillance and control measures.
- [Fröding I, David S, Yeats C, Abu-Dahab K, Albiger B, Alikhan NF, et al. Persistent spread of carbapenemase-producing Klebsiella pneumoniae in acute care hospitals in 36 European countries (the CCRE survey): a prospective, multicentre, cross-sectional, epidemiological, microbiological, and genomic surveillance study. Lancet Microbe. 2026 May 13; doi:10.1016/j.lanmic.2025.101320](https://ad-id.co/4ueY9tb)
  - Summary: In a large 2019 genomic surveillance study across 37 European countries involving 1566 carbapenem-resistant or reduced-susceptibility Klebsiella pneumoniae complex isolates from 302 hospitals, researchers found continued circulation of high-risk lineages (including ST11, ST15, ST101, and ST258/512) alongside expanding emerging clones such as ST147 and ST307, a marked increase in carbapenemase gene carriage (89.3% vs 69.6% in earlier surveillance), and rising acquisition of virulence factors, particularly within multidrug-resistant lineages, indicating ongoing and evolving hospital transmission of carbapenemase-producing K. pneumoniae and underscoring the need for strengthened genomic surveillance and infection control strategies.

**Antibiotic Stewardship and Hospital in the Home**

- [Bobbitt L, Tao L, Ereshefsky B, Ing A, Humphries R. Impact of a rapid change in beta-lactam resistance epidemiology on the management of patients with bloodstream infections. Open Forum Infect Dis. 2026 May;13(5):ofag225. doi:10.1093/ofid/ofag225](https://ad-id.co/4ufCUrd)
  - Summary: In a single-centre study of 163 patients with ceftriaxone-resistant Escherichia coli and Klebsiella pneumoniae bloodstream infections, a recent epidemiological shift was observed with an increasing proportion of blaCTX-M–negative resistance (rising to 20.3% by 2024), driven by alternative mechanisms including AmpC enzymes and non-CTX-M ESBLs (e.g., blaCMY-2, blaDHA-1, blaFOX-5, blaTEM, blaSHV variants), which led to delayed initiation of optimal therapy compared with CTX-M–positive cases despite a high overall negative predictive value (94%) for molecular CTX-M testing, supporting continued use of rapid molecular diagnostics for antimicrobial de-escalation while emphasizing the need for ongoing local surveillance as resistance epidemiology evolves.
- [Castagnola E, Mariani M, Saffioti C, Ricci E, Santaniello M, Bandettini R, et al. Artificial intelligence for analysing antibiotic use: an exploratory study in a tertiary care paediatric hospital in Italy. J Antimicrob Chemother. 2026 Jun;81(6):dkag162. doi:10.1093/jac/dkag162](https://ad-id.co/4eNQahU)
  - Summary: In a retrospective 2020–2024 study at an Italian tertiary paediatric hospital, AI-assisted analysis of antibiotic consumption and bloodstream infection resistance patterns showed increased use of Access antibiotics (>60% by 2024), reduced Watch antibiotic use, stable or declining normalized antibiotic consumption despite rising activity, and overall improvements in MRSA rates and drug resistance indices for Staphylococcus aureus, while Enterobacterales showed mixed resistance trends, with findings indicating that AI can support antimicrobial stewardship surveillance but still requires expert validation and further multicentre paediatric validation.

**Infection Prevention / Antibiotic Prophylaxis**

- [Hurley JC. Which trial do we need? The selective digestive decontamination herd effects estimation trial (SHEET) study. Clin Microbiol Infect. 2026 May 14; doi:10.1016/j.cmi.2026.05.013](https://ad-id.co/4doFuUj)
  - Summary: Selective digestive decontamination (SDD) remains a controversial ICU intervention for preventing infection in mechanically ventilated patients despite extensive evidence from over 40 randomized controlled trials and more than 30 meta-analyses showing highly variable results ranging from strong mortality benefit in early small studies (OR 0.10) to no significant effect in large contemporary multicentre trials involving ~20,000 ICU patients (OR 0.94), highlighting persistent uncertainty about its effectiveness, potential herd effects, and role in preventing ventilator-associated pneumonia and mortality.
- [Okomo U, Fitzgerald F, Feasey N, Mehtar S, Dramowski A. Infection prevention and control in low- and middle-income countries: policy, practice, and implementation challenges in sub-Saharan Africa. Clin Microbiol Rev. 2026 May 14; doi:10.1128/cmr.00142-22](https://ad-id.co/3RaFT5w)
  - Summary: This review highlights that infection prevention and control programs in sub-Saharan Africa are hindered by chronic underfunding, weak WASH infrastructure, limited laboratory and workforce capacity, and inconsistent implementation despite expanded policy frameworks, leading to a high and underreported burden of healthcare-associated infections driven mainly by Gram-negative pathogens and resistant Staphylococcus aureus; it emphasizes that effective improvement requires sustained multimodal interventions such as locally produced alcohol-based hand rub, strengthened surveillance, workforce training, improved infrastructure, and African-led innovation supported by reliable financing, leadership, and community engagement to reduce infections and antimicrobial resistance.
- [Hu H, Tursuniyazi R. The efficacy of subglottic secretion drainage in preventing ventilator-associated pneumonia in the intensive care unit: a systematic review and meta-analysis. Front Med. 2026 Apr 15;13:1758199. doi:10.3389/fmed.2026.1758199](https://ad-id.co/4eMUttR)
  - Summary: In a systematic review and meta-analysis of 14 studies involving ICU patients on mechanical ventilation, subglottic secretion drainage (SSD) was associated with a significant reduction in ventilator-associated pneumonia incidence (OR 0.42), VAP per 1,000 ventilator days, bacterial detection rates (both Gram-positive and Gram-negative organisms), and a modest reduction in ICU length of stay, suggesting that SSD is an effective nursing intervention to prevent VAP and improve selected clinical outcomes in critically ill ventilated patients.
- [Hammond NE, Devaux A, Vlok R, Adigbli D, Cuthbertson BH, Finfer SR, et al. Selective decontamination of the digestive tract in adult mechanically ventilated patients: an updated systematic review with Bayesian meta-analysis. NEJM Evid. 2026 Apr 15;5(5). doi:10.1056/EVIDoa2500264](https://ad-id.co/3PrEuXD)
  - Summary: An updated Bayesian systematic review and meta-analysis of 32 randomized clinical trials involving 27,687 mechanically ventilated ICU patients found that selective decontamination of the digestive tract (SDD) was associated with a statistically significant reduction in hospital mortality compared with standard care (relative risk 0.91; 95% credible interval 0.82–0.99) and a 99.2% posterior probability of benefit, suggesting a high likelihood that SDD reduces in-hospital death in this population.
  - **Editorial Commentary:**
    [Normand SLT. Interpreting evidence with Bayesian meta-analysis. NEJM Evid. 2026 Apr 15;5(5). doi:10.1056/EVIDe2600097](https://ad-id.co/4ucKanF)

**Bloodstream Infections and Endocarditis**

- [Kimura S, Yoshimura K, Nakasone H, Hosoi H, Ishikawa K, Uchida N, et al. Bidirectional associations between bacteremia and cytomegalovirus infection after allogeneic hematopoietic cell transplantation: JSTCT Transplant Complications Working Group. Clin Microbiol Infect. 2026 May 13; doi:10.1016/j.cmi.2026.05.010](https://ad-id.co/3R9FLDj)
  - Summary: In a nationwide Japanese registry study of 23,841 patients undergoing first allogeneic hematopoietic cell transplantation, clinically significant cytomegalovirus infection and bacteremia were found to have bidirectional associations—each increasing the risk of the other—with stronger effects observed in patients receiving letermovir prophylaxis, while both complications were independently associated with higher non-relapse mortality and worse overall survival regardless of prophylaxis status, underscoring the need for integrated infection control strategies in the post-transplant setting.
- [Sousa-Regueiro D, Alonso-Alvarez A, Balboa-Barreiro V, Fernández Noya J, Suárez Gonzalez C, Bou Arévalo G, et al. Determinants of cure and survival in vascular graft and endograft infections: a 13-year single-centre cohort study. Clin Microbiol Infect. 2026 May 11; doi:10.1016/j.cmi.2026.05.001](https://ad-id.co/4dqKCYf)
  - Summary: In a retrospective single-centre study of 171 patients with vascular graft and endograft infections between 2010 and 2023, predominantly involving extracavitary grafts and commonly caused by Staphylococcus aureus with frequent polymicrobial infection, long-term outcomes were poor with 1- and 3-year mortality rates of 25.7% and 41.5%, while higher C-reactive protein levels, polymicrobial infection, and older age independently predicted mortality and infection-related death, and both partial graft excision and graft preservation were associated with lower clinical cure rates compared with complete graft removal, highlighting the severity of VGEI and the prognostic importance of inflammatory burden and microbiological complexity.
- [Glöckner S, Wendler S, Schlichting N, Freiburger S, Doenst T, Oberbach A, et al. Staphylococcus aureus strains sustain their high-virulent phenotype during native endocarditis. Clin Microbiol Infect. 2026 May 9; doi:10.1016/j.cmi.2026.04.029](https://ad-id.co/3R9FMal)
  - Summary: In a study of paired Staphylococcus aureus isolates from blood and valve tissue in 10 infective endocarditis patients, whole-genome and phenotypic analyses showed high genetic similarity and largely preserved virulence traits (including adhesin expression, cytotoxicity, invasion, and biofilm formation) between sites, with minimal evidence of adaptive phenotypic change during native valve infection except in isolated cases, suggesting that S. aureus maintains a predominantly highly virulent wild-type phenotype in endocarditis which may contribute to ongoing cardiac tissue destruction under continuous intravascular immune pressure.
- [Calderón-Parra J, Diego-Yagüe T, Muñoz P, Fariñas-Alvarez MC, Ruiz-Alonso N, Alvarez-Uría A, et al. Effectiveness and safety of daptomycin versus vancomycin in methicillin-resistant Staphylococci left-side infective endocarditis: results from a nationwide prospective multicenter cohort. Clin Microbiol Infect. 2026 May 5; doi:10.1016/j.cmi.2026.04.027](https://ad-id.co/3R9FMqR)
  - Summary: In a prospective multicenter cohort of 617 patients with left-sided infective endocarditis caused by methicillin-resistant staphylococci, daptomycin-based regimens (n=421) showed similar in-hospital mortality to vancomycin-based therapy (adjusted OR 0.79) but were associated with significantly fewer adverse drug reactions, particularly less acute kidney injury. Since there are potential biases and confounders in observational work such as this, potential safety and outcome advantages with high-dose combination daptomycin strategies need to be confirmed in RCTs.
- [Selleng K, Ismail H, Kohler C, Wesche J, Fuhrmann J, Kohler A, et al. Reducing bacterial contamination of blood bags with a double knot. N Engl J Med. 2026 May 6;394:1865-1866. doi:10.1056/NEJMc2600589](https://ad-id.co/4wwItCU)
  - Summary: In an experimental study simulating prolonged high-level bacterial exposure of blood bag tubing, double-knot closure was compared with standard single-knot sealing and showed complete prevention of contamination versus a 9% contamination rate in single-knot bags after 35 days of incubation with high concentrations of Staphylococcus aureus, Pseudomonas aeruginosa, and Escherichia coli, suggesting that a simple, low-cost double-knot technique may substantially improve blood bag safety in resource-limited settings where standard heat-sealing equipment is unavailable.
- [Rinaldi M, Bartoletti M, Cojutti PG, Escolà-Vergé L, Fernández-Hidalgo N, Hornuss D, et al. Managing Enterococcus faecium bloodstream infection: a Delphi document on clinical recommendations and research agenda. EClinicalMedicine. 2026 May;95:103925. doi:10.1016/j.eclinm.2026.103925](https://ad-id.co/4dJdVqa)
  - Summary: In a systematic review and three-round Delphi consensus process on Enterococcus faecium bloodstream infection management, experts concluded that patient outcomes are primarily driven by comorbidities rather than vancomycin resistance status, recommended follow-up blood cultures and clinical markers to assess treatment response, supported catheter removal with short-course antibiotics in selected uncomplicated catheter-related infections, and endorsed teicoplanin for vanB infections and linezolid or high-dose daptomycin (with combination therapy reserved for severe or high-MIC cases) for vanA infections, while highlighting key research gaps and advocating standardized outcome frameworks and further trials of agents such as oritavancin.

**Bone and Joint Infections**

- [Tai DBG, Poehlein E, O'Neil JC, Nelson SB, Tande AJ, De Lima Corvino D, et al. Antibiotic duration after debridement and implant retention for early postoperative spinal implant infections: a multicenter cohort study. Clin Infect Dis. 2026 May 5; doi:10.1093/cid/ciag300](https://ad-id.co/4wBytsq)
  - Summary: In a multicenter retrospective cohort of 499 adults with early postoperative spinal implant infections managed with debridement and implant retention, extended antibiotic therapy beyond 12 weeks was not associated with reduced treatment failure compared with shorter courses across time-varying, landmark, and sensitivity analyses. Since this is an observational study and can be subject to confounders and bias, an RCT is needed to determine if individualized, potentially shorter antibiotic regimens are more favourable than longer treatment courses.

**Sexually Transmitted Infections**

- [Matthews LG, Vodstrcil LA, Wild NL, Plummer EL, Aguirre I, Htaik K, et al. Declining efficacy of moxifloxacin for Mycoplasma genitalium infection: time for fluoroquinolone-resistance guided therapy? J Infect Dis. 2026 May 8; doi:10.1093/infdis/jiag251](https://ad-id.co/4wwzh1y)
  - Summary: In a 2015–2024 observational study of 5,739 Mycoplasma genitalium infections at a Melbourne sexual health centre, moxifloxacin use increased substantially over time but clinical efficacy declined in parallel to 79.3% by 2023, likely reflecting rising fluoroquinolone resistance, while the introduction of ParC-S83 mutation testing in 2024 reduced moxifloxacin use and improved cure rates to 89.2% in ParC-S83 wildtype infections, supporting resistance-guided therapy as a strategy to optimise treatment selection and outcomes.
- [Xiao K, Si Y. Cerebral syphilitic gumma. N Engl J Med. 2026 May 9;394:1939. doi:10.1056/NEJMicm2518333](https://ad-id.co/4dpvvON)
  - Summary: In a 50-year-old man presenting with progressive headache and left-sided weakness, brain MRI revealed an enhancing right frontoparietal lesion with edema, with serology and cerebrospinal fluid testing confirming Treponema pallidum infection (positive TPPA and TRUST 1:16), and brain biopsy demonstrating plasma-cell–rich arteriolitis with spirochetes consistent with a cerebral syphilitic gumma, which markedly improved clinically and radiologically after a 2-week course of intravenous penicillin, alongside a decline in TRUST titer to 1:2.
- [Glismann L, Petersen EWH, Høstgaard S, Hoffmann S, Jensen JS, Lisby JG, et al. Syphilis and the risk of stroke: a nationwide, population-based matched cohort study. Clin Microbiol Infect. 2026 May 4; doi:10.1016/j.cmi.2026.04.025](https://ad-id.co/4eIcQ2Z)
  - Summary: In a nationwide Danish population-based matched cohort study of 3,593 syphilis-positive individuals followed for over 300,000 person-years, syphilis seropositivity was associated with a higher 10-year stroke risk compared with the general population (HR 5.4), but this association was substantially attenuated and no longer statistically significant when compared with syphilis test-negative individuals (HR 1.5), while sibling analyses similarly suggested increased stroke risk driven by shared familial or testing-related factors rather than syphilis infection itself, indicating that confounding by test indication and underlying risk profiles largely explains the observed association between syphilis testing and stroke risk.
- [Yechezkel M, Helekal D, Kapadia B, Hong V, Pomichowski ME, Reyes ICA, et al. Durability of doxycycline effectiveness against gonorrhoea after implementation of post-exposure prophylaxis in southern California, USA: a retrospective, test-negative, observational study. Lancet Infect Dis. 2026 May 7; doi:10.1016/S1473-3099(26)00123-4](https://ad-id.co/4fn0c9Q)
  - Summary: In a retrospective test-negative cohort study of 26,582 individuals in a US integrated healthcare system receiving HIV prevention or care, doxycycline post-exposure prophylaxis showed sustained effectiveness against chlamydia (66.5%) and syphilis (60.7%) but no overall protective effect against gonorrhoea (–1.8%), with a marked decline in gonorrhoea prevention efficacy over time from 42.3% to –15.0% that correlated with increasing prevalence of tetM-mediated tetracycline resistance in Neisseria gonorrhoeae, suggesting that doxycycline PEP may have durable benefits for chlamydia and syphilis but rapidly diminishing effectiveness for gonorrhoea due to antimicrobial resistance emergence.

**CNS Infections**

- [Subedi S, Harris NAP, Hall L, Paterson LD. Implementation of the BIOFIRE meningitis/encephalitis panel: a mixed-methods implementation study in a nonmetropolitan tertiary hospital. Open Forum Infect Dis. 2026 May;13(5):ofag240. doi:10.1093/ofid/ofag240](https://ad-id.co/4wwVfkO)
  - Summary: In a mixed-methods implementation study of the BIOFIRE® FILMARRAY® meningitis/encephalitis panel at an Australian tertiary hospital, clinician surveys and interviews showed high perceived usefulness of rapid CSF PCR testing and increasing confidence in discontinuing antimicrobials (from 18% to 52% for antibiotics over 12 months), with qualitative findings highlighting improved diagnostic efficiency and antimicrobial stewardship decision-making driven by education, multidisciplinary collaboration, and infectious diseases support, indicating that structured implementation strategies can enhance adoption and clinical utility of syndromic rapid diagnostics in suspected CNS infections.
- [Zhou S, Malani P. What is tetanus? JAMA. 2026 Apr 30; doi:10.1001/jama.2026.2744](https://ad-id.co/3R9y00c)
  - Summary: This patient information piece has great graphics!

**Respiratory Tract Infections**

- [Lazarus G, Caddey B, Dean A, Wangsaputra VK, Febrina F, Radiani SP, et al. Antimicrobial resistance in bacterial pathogens causing community-acquired and hospital-acquired lower respiratory tract infections (2010–24): a systematic review and meta-analysis. Lancet Microbe. 2026 May 11; doi:10.1016/j.lanmic.2026.101361](https://ad-id.co/3PnRNby)
  - Summary: In a large systematic review and meta-analysis of 336,389 respiratory isolates from 51 countries, antimicrobial resistance in lower respiratory tract infections was found to be high and geographically variable, with substantial resistance in community-acquired infections including penicillin-resistant Streptococcus pneumoniae (32.4%), ampicillin-resistant Haemophilus influenzae (51.6%), and notable third-generation cephalosporin and carbapenem resistance in Klebsiella pneumoniae, while hospital-acquired infections showed even higher resistance levels, particularly carbapenem resistance in Acinetobacter spp. (86.0%) and Pseudomonas aeruginosa (42.1%), alongside rising global trends in carbapenem-resistant Gram-negative bacteria and MRSA, highlighting marked regional and socioeconomic disparities and the growing global burden of antimicrobial resistance in respiratory infections.
- [Miles AC, Vojicic J, Peyrani P, Rosenstock S, Li H, Vietri JT, et al. Real-world effectiveness of 20-valent pneumococcal conjugate vaccine against all-cause outcomes among Medicare beneficiaries aged 65 years and older in the USA: a retrospective cohort study. Lancet Infect Dis. 2026 Apr 30; doi:10.1016/S1473-3099(26)00115-5](https://ad-id.co/3P4zTuB)
  - Summary: In a large US Medicare-based retrospective cohort study of over 16 million adults aged 65 years or older, receipt of the 20-valent pneumococcal conjugate vaccine (PCV20) was associated with reduced risk of all invasive pneumococcal disease (25.6% effectiveness), pneumococcal pneumonia (23.3%), all-cause pneumonia (15.2%), and lower respiratory tract infections (7.4%), with substantial absolute reductions in event rates within the first year of vaccination.

**Urinary Tract Infections**

- [Nordman J, Najjuuko C, Jeschke N, Snyders RE, Vazquez Guillamet MC, Shapiro J, et al. The best of both worlds: how combining a large language model and a rules-based algorithm makes CAUTI surveillance more efficient. Clin Infect Dis. 2026 May 14; doi:10.1093/cid/ciag301](https://ad-id.co/4eJ4pED)
  - Summary: In a retrospective evaluation of 919 suspected catheter-associated urinary tract infection (CAUTI) cases from a US tertiary hospital, integrating a large language model with rules-based electronic surveillance and Clinical Entity Augmented Retrieval substantially improved detection performance compared with existing algorithmic approaches, achieving up to 93.6% sensitivity and 98.6% specificity after expert adjudication, with most discrepancies due to missing symptom documentation, demonstrating that LLM-augmented surveillance can enhance accuracy and efficiency of infection prevention workflows by reducing manual chart review burden.
- [Liu M, Wang Q, Yan PJ, Li ZF, Cai YT, Xu Z, et al. Shorter- versus longer-course antibiotics in children with acute uncomplicated UTIs: a systematic review and meta-analyses. Clin Microbiol Infect. 2026 May 7; doi:10.1016/j.cmi.2026.04.022](https://ad-id.co/3PoVpKn)
  - Summary: In a systematic review and meta-analysis of 13 randomized controlled trials involving 2,010 children with acute uncomplicated urinary tract infections, shorter-course antibiotic therapy (2–5 days) was found to have similar rates of relapse, reinfection, bacteriological and clinical cure, and adverse events compared with standard longer-course therapy (≥7 days), with mostly moderate-to-high certainty evidence showing no meaningful differences in outcomes, supporting shorter antibiotic courses as a potentially effective and safe alternative while highlighting the need for further subgroup-specific research in younger children and high-risk populations.
- [Tran HT, Gomes I, Chaudhuri A, Nazari A, Ahmadi S, Végh H, et al. Targeting the disease response with NlpD and LytM for effective non-antibiotic treatment of urinary tract infections. J Infect Dis. 2026 May 6; doi:10.1093/infdis/jiag243](https://ad-id.co/4drUBwz)
  - This experimental study identifies a bacterial protein (NlpD/LytM peptide) that protects against urinary tract infection in mouse models by inhibiting host RNA polymerase II–driven inflammatory gene expression, thereby dampening excessive immune responses while paradoxically improving bacterial clearance, suggesting a potential non-antibiotic host-directed therapeutic strategy.

**Infections in Children**

- [Alam M, Lee KH, Baillie V, Mahtab S, Rahman A, Chowdhury AIC, et al. Post-mortem characterisation of pathogen-specific causes of infection-related deaths in African and south Asian neonates: a prospective, observational, multicentre study. Lancet Infect Dis. 2026 May 13; doi:10.1016/S1473-3099(26)00136-2](https://ad-id.co/4dlnrOV)
  - Summary: This large multicountry post-mortem surveillance study found that infections contributed to nearly half of neonatal deaths across LMIC sites, with Gram-negative bacteria—especially *Klebsiella pneumoniae* and *Acinetobacter baumannii*—dominating the causal pathway, and concluded that most infection-related neonatal deaths might be preventable with improved healthcare quality and more context-appropriate empirical antibiotic strategies.
- [Otoo MN, Meador KJ, Brothers T, Lavoie T, Asal NJ, Quilliam BJ, et al. Prenatal azithromycin exposure and risk of neurodevelopmental disorders in children. JAMA Netw Open. 2026;9(5):e2612051. doi:10.1001/jamanetworkopen.2026.12051](https://ad-id.co/4eT5Ss9)
  - Summary: In a retrospective cohort study of 15,527 mother–infant dyads using US administrative claims data (2012–2024), late-pregnancy exposure to azithromycin was associated with a lower risk of neurodevelopmental disorders—particularly speech and language disorders—compared with exposure to other antibiotics (adjusted HR for overall NDDs 0.69, 95% CI 0.49–0.98; SLD 0.59, 0.39–0.91), while showing no statistically significant difference versus no antibiotic exposure, suggesting a potential protective association that requires confirmation in larger and more diverse populations.
- [Burstein B, Xie J, Kuppermann N. Bacteremia and bacterial meningitis among low-risk febrile infants aged 29 to 60 days. JAMA Pediatr. 2026 Apr 27; doi:10.1001/jamapediatrics.2026.0971](https://ad-id.co/4wNL6AL)
  - Summary: This pooled international diagnostic accuracy study evaluated the updated PECARN prediction rule (based on urinalysis, procalcitonin, and absolute neutrophil count) for febrile infants aged 29–60 days and aimed to identify low-risk infants for invasive bacterial infections, supporting safer reduction of unnecessary lumbar punctures, antibiotic use, and hospitalisation in well-appearing infants while addressing ongoing guideline non-adherence in clinical practice.

**Mycobacterial Infections**

- [Shivkumar PV, Rannaware A, Deshmukh RD, Bhide SM, Deotale VS, Jain J, et al. Performance of TB antigen-based skin tests amongst healthcare workers – Sevagram, India, 2023–2024. Open Forum Infect Dis. 2026 May 12;ofag268. doi:10.1093/ofid/ofag268](https://ad-id.co/4dvDTMR)
  - Summary: A prospective cohort study of healthcare workers in India found a 28.5% prevalence of tuberculosis infection and demonstrated that the novel antigen-based skin test SIILTIBCY® (CY-TB) showed comparable diagnostic performance to QuantiFERON-TB Gold In-Tube Plus, supporting its potential as a practical and low-cost screening tool for resource-limited healthcare settings.
- [Kim TO, Kwak N, Jeon D, Jhun BW, Jo KW, Kang YA, et al. Impact of vitamin D deficiency on 6-month culture conversion in nontuberculous mycobacterial pulmonary disease. Sci Rep. 2026 May 1. doi:10.1038/s41598-026-51274-8](https://ad-id.co/4dHXpqu)
  - Summary: In a multicenter cohort of 485 patients with nontuberculous mycobacterial pulmonary disease, baseline vitamin D deficiency was not associated with 6-month culture conversion or time to conversion after treatment, while guideline-based therapy was strongly associated with improved culture conversion outcomes.

**Fungal Infections and antifungal agents**

- [Das S, Singh S, Tawde Y, Spruijtenburg B, Chakrabarti A, Rudramurthy SM, et al. Trends in antifungal resistance and mechanistic insights into azole resistance in Candidozyma auris: a 13-year study of a comprehensive set of clinical isolates from India. Clin Infect Dis. 2026 Apr 30;ciag257. doi:10.1093/cid/ciag257](https://ad-id.co/4wuLHHf)
  - Summary: A 13-year multicentre study of 596 Candidozyma auris isolates across India revealed persistently high resistance to fluconazole and increasing resistance to amphotericin B and caspofungin, with 3.2% of isolates being multidrug-resistant, while genetic and expression analyses showed that azole resistance is associated not only with ERG11 mutations and efflux pump activity but likely also involves additional stress-response pathways contributing to the evolution of this pathogen.
  - **Editorial Commentary:**
    [Sprute R, Cornely OA. Rising antifungal resistance in Candidozyma auris: signals, gaps, and the path forward. Clin Infect Dis. 2026 May 2; doi:10.1093/cid/ciag258](https://ad-id.co/4dvDUjT)
- [Yoshida H, Adelman MW, Ifiora F, Pérez MA, Niravath M, Yoshimura H, et al. Utility of repeating blood cultures in candidemia: insights from 2 large clinical data repositories. Clin Infect Dis. 2026 May 14; doi:10.1093/cid/ciag216](https://ad-id.co/4dvDUAp)
  - Summary: In a retrospective analysis of 1,386 candidemia episodes across two electronic health record datasets, repeat blood cultures identified a substantial proportion of cases that were initially missed (23% at Houston Methodist Hospital and 45% in MIMIC-IV), supporting the clinical utility of repeating blood cultures when candidemia is strongly suspected despite initial negative results.
- [Di Pilato V, Willison E, Calabrese L, Matarazzo E, Magnasco L, Costa E, et al. Genomic landscape of Candidozyma auris in Italy, 2019 to 2025: emerging diversity of clade I sub-lineages associated with inter-facility transmission and cross-border transfers. Euro Surveill. 2026;31(18):2500730. doi:10.2807/1560-7917.ES.2026.31.18.2500730](https://ad-id.co/3R99mwK)
  - Summary: In a genomic surveillance study of 85 Italian *Candidozyma auris* isolates (68 from databases and 17 newly sequenced) compared with 139 international genomes, all strains belonged to South Asian clade I but were distributed across multiple divergent subclades (including a dominant Ic lineage with HMG1 P238H mutation and a potential new Id subclade), demonstrating repeated introductions and regional nosocomial spread with lineage-specific antifungal resistance mutations (CDR1, ERG11, TAC1B), highlighting ongoing transmission complexity and the need for strengthened screening and infection control across healthcare facilities.
- [de Melo Teixeira M, Gomes Gonçalves F, Alves Vianna L, Guerra Tenório B, Gomes de Brito Alves L, Durlacher RR, et al. Revisiting the natural history of Jorge Lobo’s disease: a mycological enigma spanning unresolved taxonomy, uncertain transmission, and inadequate therapy. Clin Microbiol Rev. 2026 May 14; doi:10.1128/cmr.00302-25](https://ad-id.co/3R83x2B)
  - Summary: This review synthesizes the historical, clinical, diagnostic, and ecoepidemiological evidence on Jorge Lobo’s disease, a neglected chronic cutaneous and subcutaneous mycosis endemic to the Amazon Basin, highlighting its characteristic keloid-like lesions, reliance on microscopy and histopathology for diagnosis due to limited culture and molecular tools, challenges in differentiating it from other chronic skin diseases, and variable treatment responses including surgery and prolonged azole therapy, while also presenting new phylogenomic insights reclassifying the causative organism within the *Paracoccidioides* genus and calling for improved surveillance, standardized diagnostics, and research into environmental reservoirs and host–pathogen interactions.
- [Lalljie A, Budvytiene I, Wheat JL, Theel ES, Banaei N. Accuracy of plasma cell-free DNA PCR for diagnosis of coccidioidomycosis. Clin Infect Dis. 2026 May 5; doi:10.1093/cid/ciag287](https://ad-id.co/4dvDV7r)
  - Summary: In a retrospective analysis of 362 plasma samples from patients with suspected coccidioidomycosis, Coccidioides plasma cell-free DNA PCR demonstrated moderate sensitivity (57.5% overall, varying by disease phenotype from 33.3% in focal pulmonary disease to 66.7% in multifocal pulmonary disease) but very high specificity (100%) and excellent positive and negative predictive values, suggesting that while the assay is not sufficiently sensitive to rule out disease, it provides a highly specific, rapid, non-invasive diagnostic tool that may complement conventional fungal testing for Coccidioides immitis and C. posadasii infection.
  - **Editorial Commentary:**
    [Donovan FM. A road not yet taken: cell-free DNA PCR testing for coccidioidomycosis. Clin Infect Dis. 2026 May 5; doi:10.1093/cid/ciag288](https://ad-id.co/4dvDVnX)
- [Bavastro M, Magnasco L, Mezzogori L, et al. Evaluation of persistence of *Candidozyma auris* colonization. Clin Microbiol Infect. 2026 May 9; doi:10.1016/j.cmi.2026.05.003](https://ad-id.co/4dvDVEt)
  - Summary: In a single-centre prospective study of 50 patients colonized with *Candidozyma auris* discharged from a previously endemic ICU, longitudinal PCR and culture-based surveillance showed that approximately 59% of patients cleared colonization within a median of 5 months (IQR 3–7), while 38% remained persistently colonized (with some progressing to candidemia), with persistence associated with higher functional status and longer antimicrobial exposure, suggesting that colonization duration may be shorter than the commonly assumed 12 months and that re-evaluation strategies for infection control precautions may be warranted.
- [Wang L, Jiang L, Liu S, Yi M. *Plectosphaerella cucumerina* fungal keratitis in an uncommon presentation. Clin Microbiol Infect. 2026 May 4; doi:10.1016/j.cmi.2026.04.024](https://ad-id.co/4dvDVUZ)
  - Summary: This case report describes a 71-year-old male farmer with severe right-eye fungal keratitis likely acquired through soil exposure and hand-to-eye inoculation, presenting with pain, corneal stromal infiltrate, and hypopyon, with in vivo confocal microscopy showing filamentous fungal structures and intraoperative confirmation of infection, who was managed with urgent surgical debridement, intrastromal and subconjunctival voriconazole injections, and anterior chamber irrigation, followed by microbiological culture confirmation from corneal scraping.

**Diagnostics**

- [Yusuf E, van den Bijlaardt W, van Dessel H, Dierikx T, van Hattem J, ten Have S, et al. Multicenter validation of EUCAST disk diffusion for frequently encountered anaerobic bacteria (excluding Cutibacterium acnes) in clinical microbiology laboratories in the Netherlands. Clin Microbiol Infect. 2026 May 13; doi:10.1016/j.cmi.2026.05.009](https://ad-id.co/4dvDWbv)
  - Summary: In a multicentre evaluation of the EUCAST disk diffusion method for anaerobic antimicrobial susceptibility testing using Fastidious Anaerobe Agar with horse blood (FAA-HB) from three manufacturers, performance was generally acceptable for *Bacteroides spp.*, *Fusobacterium necrophorum*, and *Clostridium perfringens*, with categorical agreement typically >85–90% for most antibiotic–organism combinations, but important limitations were identified including poor performance for β-lactam/β-lactamase inhibitor testing (particularly piperacillin–tazobactam in *Bacteroides spp.* with high very major discrepancies), frequent uninterpretable results in *Prevotella spp.* due to poor growth, and overall evidence that while FAA-HB is suitable for routine anaerobic disk diffusion AST, its reliability varies substantially by organism–antibiotic combination and manufacturer.
- [Tsalik EL, Prasad NK, Fleisher LA. Advancing antimicrobial stewardship with diagnostic excellence: the role of Bayesian reasoning. Open Forum Infect Dis. 2026 Apr;13(4):ofag213. doi:10.1093/ofid/ofag213](https://ad-id.co/4wApLdW)
  - Summary: This perspective article argues that antimicrobial stewardship should be strengthened by integrating diagnostic stewardship with Bayesian reasoning, proposing that clinical guidelines incorporate explicit probability thresholds to guide testing and antibiotic initiation, thereby improving decision-making under diagnostic uncertainty; it further emphasizes clinician training in Bayesian thinking and the use of real-time decision-support tools to reduce inappropriate antibiotic use, improve patient safety, and mitigate the progression of antimicrobial resistance.

**Improving Clinical Research**

- [Quartagno M, Ghorani E, Morris TP, Bern H, Clements MN, Walker AS, et al. How to design a ROCI (Response Over Continuous Intervention) randomised trial: guidance and a case study. BMC Med Res Methodol. 2026 Apr 25; doi:10.1186/s12874-026-02786-4](https://ad-id.co/4dvDWs1)
  - Summary: This methodological paper describes the Response Over Continuous Intervention (ROCI) randomized trial design as an alternative to traditional non-inferiority trials for optimizing continuous treatment parameters such as dose, duration, and dosing frequency, outlining key considerations including flexible treatment–response modelling (using fractional polynomials), arm selection, and power definitions; it illustrates the approach using the REFINE-Lung trial, which evaluates extended pembrolizumab dosing intervals (6- to 18-weekly) in advanced non-small cell lung cancer with a primary endpoint of 2-year overall survival, demonstrating how simulation-based and adaptive strategies can improve efficiency, ethical balance, and precision in identifying optimal treatment regimens.

**General Interest**

- [Mora-Rillo M, Gupta N, Gkrania-Klotsas E, Drexler JF, Jokelainen P, Tsiodras S, et al. Andes hantavirus outbreak on a cruise ship – an ESCMID Emerging Infections Subcommittee (EIS) rapid assessment. Clin Microbiol Infect. 2026 May 12; doi:10.1016/j.cmi.2026.05.016](https://ad-id.co/4dK5par)
  - Summary: This commentary describes an ongoing multinational outbreak of Andes hantavirus associated with the cruise ship MV Hondius in April–May 2026, involving at least eight linked cases (five confirmed, three suspected) including three deaths and additional severe cases requiring evacuation, with infections identified across multiple countries following exposure on board a 173-person, 23-nationality voyage departing Ushuaia; it highlights ongoing epidemiological investigations, including potential secondary cases detected after international travel, and emphasizes the complexities of outbreak control, surveillance, and intersectoral coordination under International Health Regulations in a confined maritime setting.
- [Fish D. Getting serious about tickborne diseases — shifting research priorities. N Engl J Med. 2026 May 13; doi:10.1056/NEJMp2516974](https://ad-id.co/4dKXf1y)
  - Summary: The article discusses the steadily rising incidence and economic burden of Lyme disease and other tickborne infections in the United States despite decades of research, arguing that prevention efforts have been misdirected toward human vaccines and pathogen-focused approaches rather than targeting the primary vector—Ixodes ticks maintained by deer populations—and proposes that more effective control strategies should prioritize ecological interventions such as deer-targeted vaccines, wildlife management, and tick population reduction to interrupt transmission at its environmental source.
- [Jeng WJ, Yip TCF, Lok AS. Hepatitis B: a review. JAMA. 2026 May 4; doi:10.1001/jama.2026.6070](https://ad-id.co/4dvDWZ3)
  - Summary: Hepatitis B virus (HBV) infection remains a major global health burden affecting about 254 million people and causing approximately 1.1 million deaths annually, with most chronic infections arising from perinatal transmission, and while universal birth-dose vaccination combined with hepatitis B immune globulin and maternal antiviral therapy can reduce transmission to under 1%, global vaccination coverage remains suboptimal at about 45%; chronic infection is diagnosed via serologic markers and viral DNA levels, can progress to cirrhosis and hepatocellular carcinoma, and is managed with antiviral therapies such as entecavir or tenofovir or pegylated interferon, which suppress viral replication, reduce liver disease progression, and lower hepatocellular carcinoma risk, with long-term management including routine fibrosis assessment and cancer surveillance in high-risk patients.
- [Dubin I, Schattner A. Ecchymosis—a neglected sign of necrotizing fasciitis. QJM. 2026 Apr;119(4):319-320. doi:10.1093/qjmed/hcaf207](https://ad-id.co/4dvDXfz)
  - Summary: A 72-year-old man on chronic hemodialysis for diabetic nephropathy presented with rapidly progressive severe leg pain, fever, and markedly elevated inflammatory markers initially treated as cellulitis, but despite non-diagnostic CT imaging showing only subcutaneous edema, evolving findings including lymphangitis and ecchymosis raised concern for necrotizing soft tissue infection, which was confirmed intraoperatively during urgent fasciotomy, leading to clinical improvement after broad-spectrum antibiotics and surgical intervention, although all cultures remained negative.
- [Michel K, Pruthi P, Corke C. Capnocytophaga canimorsus septicaemia presenting with multiorgan dysfunction syndrome (MODS) and disseminated intravascular coagulation (DIC) in an immunocompetent individual. Crit Care Case Rep. 2026 May 6; doi:10.1155/crcc/1505682](https://ad-id.co/3Pgbnql)
  - Summary: A previously healthy 52-year-old woman developed fulminant Capnocytophaga canimorsus sepsis following a minor dog-related abrasion despite prior amoxicillin/clavulanate, rapidly progressing to septic shock with disseminated intravascular coagulation, multiorgan failure, and acute renal failure requiring continuous renal replacement therapy, with blood cultures confirming infection and imaging showing bilateral renal cortical necrosis leading to permanent dialysis dependence, highlighting the need for early recognition of Capnocytophaga spp. in zoonotic exposure–associated sepsis even in immunocompetent patients.
- [Tejo AM, Duarte-Neto AN, Rena JA, Kruschewsky WLL, Romano CM, de Souza CS, et al. Fatal transfusion-transmitted severe dengue in an immunocompromised host: case report and systematic literature review. Clin Infect Dis. 2026 May 5;ciag292. doi:10.1093/cid/ciag292](https://ad-id.co/4wucljp)
  - Summary: This report describes a fatal case of transfusion-transmitted severe dengue in a hematopoietic stem cell transplant recipient during the 2024 dengue epidemic in Brazil, with phylogenetic evidence confirming transmission and underscoring the serious risks of dengue transmission through blood transfusion, particularly among immunocompromised patients.
- [Bernigaud C, Do-Pham G, Guichard E, Beylot-Barry M, Goujon E, Isnard C, et al. Combined oral ivermectin and 5% permethrin cream to treat severe scabies. N Engl J Med. 2026 May 6;394:1814-1823. doi:10.1056/NEJMoa2411721](https://ad-id.co/3Re3EcU)
  - Summary: In this blinded randomized clinical trial of 132 adults with severe scabies, participants received either higher-dose oral ivermectin (400 μg/kg) or standard-dose ivermectin (200 μg/kg) in combination with topical 5% permethrin, and results showed no superiority of the higher dose, with similar cure rates (75% vs 82%) and no safety concerns, indicating that standard-dose ivermectin plus permethrin remains an effective and sufficient regimen for severe scabies treatment.
- [Leroux-Roels I, Huang G, Ferguson M, Kohli A, Clark R, Bickel M, et al. Efficacy and safety of an mRNA seasonal influenza vaccine in adults. N Engl J Med. 2026 May 6;394:1803-1813. doi:10.1056/NEJMoa2516491](https://ad-id.co/4eJIJbv)
  - Summary: In a phase 3 double-blind randomized trial involving 40,703 adults aged 50 years and older, an investigational trivalent mRNA influenza vaccine (mRNA-1010) demonstrated superior efficacy compared with a licensed standard-dose influenza vaccine, reducing RT-PCR–confirmed influenza-like illness with a relative vaccine efficacy of 26.6% and meeting predefined noninferiority and superiority criteria, although it was associated with higher rates of mostly mild to moderate solicited adverse reactions such as injection-site pain, fatigue, headache, and myalgia.

**Target Trial Emulation**

- [Wong K, et al. Concordance between target trial emulation and randomised controlled trials: systematic review and meta-analysis. BMJ. 2026;393:e086810. doi:10.1136/bmj-2025-086810](https://ad-id.co/4nWJksV)
  - Summary: This systematic review and meta-analysis of 107 target trial emulation–randomised controlled trial pairs found moderate overall concordance between emulated and trial results (Pearson correlation 0.59, 79% agreement, ratio of ratios 0.96), with substantially higher agreement when emulation closely replicated trial design (correlation 0.83, 87% agreement), while also showing systematic outcome-dependent bias (underestimation for venous thromboembolism and major adverse cardiovascular events, overestimation for respiratory outcomes) and lower concordance in claims-based emulations, with poorer agreement linked to imbalanced baseline characteristics, hospital-initiated treatment, and lower outcome emulation quality, suggesting that improving baseline alignment, outcome definition, and use of linked multi-source data could enhance emulation validity. There has been online criticism about the methods used in this systematic review and meta-analysis (eg, on “X” by “NonsparseOncologist”)

**Trial Protocols / Trial Ideas**

- [Papp M, Turan C, Kiss N, Zubek L, Karim DM, Hegyi P, et al. Kinetics of procalcitonin to reduce unnecessary antibiotic use (KIPRUN): protocol for a multicentre, randomised superiority trial to compare the efficacy and safety of procalcitonin kinetics-guided and absolute procalcitonin value-guided antibiotic initiation in critically ill patients. BMJ Open. 2025; doi:10.1136/bmjopen-2025-115344](https://ad-id.co/3PysO5I)
  - Summary: This multicentre randomised controlled trial will compare a procalcitonin (PCT)-guided “kinetics” strategy (initiating antibiotics when PCT ≥0.5 ng/mL and increases >100% from the previous day) versus a standard “absolute” threshold strategy (PCT ≥0.5 ng/mL) in hemodynamically stable critically ill adults with suspected infection, with 250 participants planned, to determine whether incorporating PCT kinetics reduces unnecessary antibiotic therapy assessed at 72 hours by an independent adjudication panel, addressing a gap in evidence despite current guideline uncertainty on PCT-guided antibiotic initiation in ICUs.
- [Gallichan S, Lewis JM, Forrest S, Moore M, Picton-Barlow E, McKeown C, et al. A protocol for the TRACS-Liverpool study, tracking transmission of extended-spectrum beta-lactamase producing Enterobacterales across health and social care settings in the United Kingdom. 2026; doi:10.64898/2026.05.13.26352872](https://ad-id.co/3RxSTT2)
  - Summary: This protocol paper describes the TRACS Liverpool genomic surveillance programme designed to map transmission of extended-spectrum beta-lactamase-producing Enterobacterales (ESBL-E) across hospitals, intermediate care, and long-term care facilities in Liverpool, UK by integrating patient and healthcare facility data with standardised stool, rectal, environmental, and staff sampling, whole-genome sequencing, and statistical modelling to identify key transmission points and acquisition events, addressing current gaps in understanding of antimicrobial-resistant bacterial spread across the healthcare continuum and providing a reproducible framework to inform interventions targeting both resistant and broader enteric pathogen transmission.

**Antibiotic Therapy Reviews**

- [Frimodt-Møller N, Hertz FB. Pivmecillinam: past, present, and future. Expert Opin Drug Metab Toxicol. 2026 May 18; doi:10.1080/14656566.2026.2672731](https://ad-id.co/4feTJ0O)
  - Summary: This literature review of pivmecillinam, an oral prodrug of mecillinam widely used in Nordic countries for uncomplicated urinary tract infections, outlines its mechanism of action as a narrow-spectrum amidinopenicillin active against common uropathogens such as Escherichia coli with low resistance rates (~5%), describes its pharmacokinetics, pharmacodynamics, resistance stability against many broad-spectrum beta-lactamases, and safety profile, and highlights its clinical role as empiric and targeted therapy for uncomplicated UTI and occasionally pyelonephritis and bacterial diarrhoea, while noting ongoing uncertainty regarding optimal dosing and treatment duration despite its favourable microbiome-sparing properties.

**PK/PD and Drug Dosing**

- [Coyne AJK, Gray R, Gonnabathula P, May ES, Puccio P, Wu C. Durability and mechanistic performance of aztreonam/avibactam versus aztreonam plus ceftazidime/avibactam against *Stenotrophomonas maltophilia* complex. J Antimicrob Chemother. 2026 Jun;81(6):dkag177. doi:10.1093/jac/dkag177](https://ad-id.co/3PQ0b3N)
  - Summary: This hollow fibre infection model study compared aztreonam/avibactam with aztreonam plus ceftazidime/avibactam against Stenotrophomonas maltophilia complex under human-simulated prolonged dosing regimens and found that at lower dosing (6 g/day) neither regimen achieved sustained bactericidal activity, while at higher exposure (8 g/day) only continuous-infusion aztreonam/avibactam produced rapid and durable bactericidal effects with stable MICs and reduced blaL1/blaL2 induction, whereas the combination with ceftazidime led to early killing followed by regrowth and greater β-lactamase upregulation, suggesting that optimized aztreonam/avibactam exposure alone may be superior for achieving sustained antimicrobial activity against this intrinsically resistant pathogen.
- [Nothofer S, Dumps C, Dorn C, Lier C, Weiss M, Zeitlinger M, et al. Impact of cardiopulmonary bypass on cefuroxime plasma and interstitial tissue concentrations in patients undergoing cardiac bypass surgery—a prospective controlled clinical trial. J Antimicrob Chemother. 2026 Jun;81(6):dkag171. doi:10.1093/jac/dkag171](https://ad-id.co/4dSKPDV)
  - Summary: This pharmacokinetic study in patients undergoing on- versus off-pump coronary artery bypass graft surgery assessed plasma and interstitial fluid cefuroxime concentrations after standard surgical prophylaxis dosing (3 g pre-incision plus 1.5 g repeat dose at CPB weaning or sternal closure) and found no significant differences between groups despite cardiopulmonary bypass-related physiological changes, with adequate exposure maintained above an 8 mg/L MIC for approximately 4.5–4.8 hours, suggesting that current cefuroxime prophylaxis regimens provide sufficient tissue and plasma coverage in cardiac surgery irrespective of CPB use.
- [Joffe AR, Humber A, Bates A, Lipman J, Wallis S, Roberts J, et al. Achieving therapeutic antibiotic levels during intermittent dosing of meropenem and piperacillin-tazobactam in critically ill children: the ATACC study. Antimicrob Agents Chemother. 2026 May 28; doi:10.1128/aac.01968-25](https://ad-id.co/4u9x8qa)
  - Summary: This prospective observational study in two pediatric intensive care units evaluated pharmacodynamic target attainment (fCmin>MIC and fCmin>4×MIC) during the first 48 hours of high-dose 6-hour infusions of meropenem or piperacillin–tazobactam in 49 critically ill children and found that target attainment was frequently suboptimal (only 34–36% achieving fCmin>MIC and 6–11% achieving fCmin>4×MIC), augmented renal clearance was common and independently associated with failure to achieve target concentrations at 48 hours, potentially neurotoxic levels were rare, and achieving pharmacodynamic targets did not correlate with time to clinical resolution of severe sepsis signs.

**Antibiotics - In vitro susceptibility**

- [Alanbari N, Amoura A, Guérin F, Cosson G, Pistien C, Magreault S, et al. Temocillin efficacy and emergence of resistance in *Enterobacter cloacae* complex in a murine peritonitis model. J Antimicrob Chemother. 2026 Jun;81(6):dkag151. doi:10.1093/jac/dkag151](https://ad-id.co/4fPwMBn)
  - Summary: This in vitro and high-inoculum murine peritonitis model study evaluated temocillin against Enterobacter cloacae complex (ECC) and found frequent and rapid emergence of resistant mutants across most tested temocillin-susceptible isolates after short exposure, with corresponding in vivo experiments showing higher mortality and resistance development in mice infected with Enterobacter asburiae compared with cefepime, despite some strain-dependent variability, leading to the conclusion that temocillin may be unreliable for severe ECC infections even when initial susceptibility is demonstrated due to its propensity for rapid resistance selection.
- [Cantón R, García-Castillo M, Hernández-García M, Sastre-Femenía MÀ, López-Causapé C, Ruiz-Garbajosa P, et al. Cefiderocol activity against multidrug-resistant isolates from the PERSEUS study: relationship between cefiderocol minimum inhibitory concentration and clinical outcomes. J Antimicrob Chemother. 2026 Jun;81(6):dkag164. doi:10.1093/jac/dkag164](https://ad-id.co/4nYhix6)
  - Summary: This retrospective PERSEUS study of cefiderocol use under compassionate and early access programmes in Spain evaluated 57 multidrug-resistant Gram-negative isolates, predominantly Pseudomonas aeruginosa, and found an overall 86% susceptibility rate by EUCAST 2024 criteria with high concordance between commercial and reference broth microdilution methods, while whole-genome sequencing identified frequent metallo-β-lactamases and iron transport gene mutations among resistant isolates; importantly, clinical cure was sometimes achieved even in patients infected with isolates classified as resistant, highlighting discordance between MIC-based resistance and clinical outcomes and underscoring the need for standardized susceptibility testing and improved genotype–phenotype correlation.

**Beta-Lactamases and Other Resistance Mechanisms**

- [Joyce C, Anwar S, Hind C, Sutton JM, Sergaki C. Clinical impact of bacterial heteroresistance. JAC Antimicrob Resist. 2026 Jun;8(3):dlag075. doi:10.1093/jacamr/dlag075](https://ad-id.co/4nTaPTY)
  - Summary: This review describes bacterial heteroresistance as the presence of resistant subpopulations within an apparently antibiotic-susceptible bacterial population across multiple species and antibiotics, highlights its frequent under-detection in routine clinical microbiology due to limitations of standard susceptibility testing methods, and argues that failure to identify heteroresistance may contribute to misleading susceptibility classifications and suboptimal treatment outcomes, thereby calling for increased clinical awareness, incorporation into antimicrobial susceptibility testing guidelines, and development of improved diagnostic methods to better detect and manage heteroresistant infections.
- [Dorazio AJ, Aitken SL, Pierce VM, Kline EG, Squires KM, Pogue JM, et al. First report of treatment-emergent resistance to cefepime-zidebactam in *Pseudomonas aeruginosa*. Clin Infect Dis. 2026 Jan 15;82(1):44-8. doi:10.1093/cid/ciaf638](https://ad-id.co/3Rx4Lol)
  - Summary: This case report describes the first documented emergence of cefepime-zidebactam resistance leading to clinical failure in a patient with Pseudomonas aeruginosa pneumonia, where the organism’s MIC increased from 8/8 to 32/32 mg/L during therapy, and whole-genome sequencing identified novel mutations in the MexAB-OprM efflux pump operon associated with cross-resistance to other β-lactam/β-lactamase inhibitor combinations including ceftazidime-avibactam and imipenem-relebactam, highlighting the potential for rapid adaptive resistance and multidrug cross-resistance during treatment.
- [Itoh N, Kawabata T, Akazawa N, Hayakawa K, Suzuki M, Sakurai A, et al. Carbapenem-resistant Gram-negative bacilli infections in Japanese patients with cancer. Sci Rep. 2026 May 23; doi:10.1038/s41598-026-54591-0](https://ad-id.co/4famknS)
  - Summary: This multicenter prospective cohort study of 167 Japanese patients with carbapenem-resistant Gram-negative bacilli (CRGNB) infections compared outcomes between those with and without cancer and found no significant differences in 30-day mortality or length of hospital stay in crude and propensity-adjusted analyses, although cancer patients had a significantly higher risk of a composite adverse outcome (30-day mortality or clinical deterioration; OR 2.36), indicating that while mortality alone was not statistically different, CRGNB infections in cancer patients are associated with poorer overall clinical trajectories and underscore the need for enhanced preventive strategies in this high-risk population.
- [Rasmussen A, Porsbo LJ, Roer L, Sydenham TV, Hallstrøm S, Schønning K, et al. Gene transfer from NDM-5-producing and OXA-48-producing *Enterobacter hormaechei* ST79 on contaminated dicloxacillin capsules to other Enterobacterales in Europe, 2020–23: a retrospective, observational, molecular epidemiological study. Lancet Microbe. 2026 May 21; doi:10.1016/j.lanmic.2026.101354](https://ad-id.co/4dz22Dm)
  - Summary: Wow!! This retrospective genomic and epidemiological investigation of a large Danish outbreak linked to contaminated dicloxacillin capsules carrying Enterobacter hormaechei ST79 with blaNDM-5 and blaOXA-48 found that while initial clonal surveillance identified only a subset of cases, expanded plasmid-specific screening and long-read sequencing revealed extensive undetected dissemination of outbreak-associated IncX3 and IncL plasmids across multiple Enterobacterales species in 1829 isolates, demonstrating that the outbreak evolved from a single clonal event into a broader plasmid-mediated transmission network and highlighting the limitations of conventional strain-based outbreak detection and the importance of plasmid-focused genomic surveillance.
- [Barman J, Biswas P, Roy P, Hazra S. A divergent MBL-fold metallo-hydrolase (Kmh-1) from *Klebsiella pneumoniae* confers aztreonam degradation and expands the β-lactamase landscape. ACS Infect Dis. 2026 May 19; doi:10.1021/acsinfecdis.5c01093](https://ad-id.co/3Psm2OU)
  - Summary: This functional and structural characterization study describes Kmh-1, a novel metallo-β-lactamase-fold enzyme identified in Klebsiella pneumoniae with low sequence similarity to classical B1 metallo-β-lactamases and a divergent zinc-binding motif retaining only key residues, demonstrating that it requires zinc for activity and is capable of hydrolysing a broad range of β-lactams including aztreonam.
- [Mylona E, Kostourou S, Giankoula D, Kolokotroni C, Tsilikis P, Koudoumnakis N, et al. A seven-year study of carbapenem-resistant *Klebsiella pneumoniae* bloodstream infections in a tertiary hospital in Greece: a shift toward metallo-β-lactamase and dual carbapenemase strains. Antibiotics (Basel). 2026 May 13;15(5):491. doi:10.3390/antibiotics15050491](https://ad-id.co/4dM2RsJ)
  - Summary: This seven-year (2019–2025) single-centre study of 671 carbapenem-resistant Klebsiella pneumoniae bloodstream infection isolates in a Greek tertiary hospital found a shifting epidemiology with declining KPC producers after 2022 and a concurrent rise in dual carbapenemase-producing strains (KPC plus VIM/NDM) and single metallo-β-lactamase producers, particularly NDM, alongside increasing colistin resistance.
- [Egge SL, Kang D, Pouya N, Thornton PC, Flores E, Clarke K, et al. Genomic and clinical features associated with reduced susceptibility to cefiderocol and cross-resistance to β-lactam/β-lactamase inhibitors in a global cohort of *Pseudomonas aeruginosa*. J Infect Dis. 2026 May 15; doi:10.1093/infdis/jiag263](https://ad-id.co/4dOFI7x)
  - Summary: This global Prospective Observational Pseudomonas (POP) study analysed 186 carbapenem-resistant Pseudomonas aeruginosa isolates from 10 countries using whole-genome sequencing, cefiderocol susceptibility testing, and hollow-fibre infection modelling, finding that 5.9–10.8% were non-susceptible and up to 15.1% exhibited heteroresistance depending on breakpoints, with reduced susceptibility linked to diverse mechanisms including PirRS-regulated siderophore receptor alterations, AmpC and cpxS mutations, and presence of blaNDM and blaVEB, and independently associated with resistance to ceftolozane/tazobactam and ceftazidime/avibactam, while experimental models showed resistance emergence under simulated human exposures in heteroresistant but not fully susceptible isolates, collectively indicating that cefiderocol non-susceptibility is globally distributed across lineages and raising concerns about its durability as a salvage therapy for multidrug-resistant P. aeruginosa infections.
- [Gottesdiener LS, Li Y, Greenwood-Quaintance KE, Komarow L, Arias CA, Cober E, et al. Resistance to novel β-lactam/β-lactamase inhibitors among carbapenem-resistant *Pseudomonas aeruginosa* and clinical implications in the prospective observational Pseudomonas study. Antimicrob Agents Chemother. 2026 May 15; doi:10.1128/aac.00388-25](https://ad-id.co/4dEdyNU)
  - Summary: This multinational study of 800 carbapenem-resistant Pseudomonas aeruginosa isolates from 35 hospitals across nine countries assessed susceptibility to novel β-lactam/β-lactamase inhibitor combinations and found moderate overall activity for ceftolozane-tazobactam (69%) and ceftazidime-avibactam (67%), but substantially lower susceptibility to imipenem-relebactam (33%), with markedly poorer activity in carbapenemase-producing isolates and outside the USA.

**Adverse Effects from Antimicrobial Agents**

- [Özdede M, Balli Turhan FN, Geridönmez Ö, Kara E, Metan G. Comparative nephrotoxicity of polymyxin B versus colistin: evidence from ensemble learning and propensity score matching. J Antimicrob Chemother. 2026 Jun;81(6):dkag044. doi:10.1093/jac/dkag044](https://ad-id.co/4fNlfm3)
  - Summary: This retrospective analysis of 258 patients compared nephrotoxicity between colistin and polymyxin B for multidrug-resistant Gram-negative infections and found that colistin was associated with significantly higher rates of acute kidney injury (KDIGO stage II or higher) than polymyxin B in both unmatched (31% vs 19%) and propensity-matched cohorts (34.4% vs 14.1%). Complete renal recovery occurred in only 41% of cases within 30 days. As an observational study this is subject to a number of biases and confounders. These results are yet to be confirmed in an RCT.

**Antibiotic Stewardship and Hospital in the Home**

- [Kuijpers SME, Siepel M, Schut MC, van Hest R, Spijker R, Schade RP, et al. Methods for determining indications for antibiotic prescriptions to facilitate antimicrobial stewardship: a scoping review. Clin Microbiol Infect. 2026 May 24; doi:10.1016/j.cmi.2026.05.021](https://ad-id.co/4dDqa7Z)
  - Summary: This scoping review of 157 studies on methods for determining antibiotic prescription indications for antimicrobial stewardship found that most research was conducted in high-income, multicentre, retrospective settings and relied either on manual extraction (43%) or automated approaches (57%). Overall, the review highlights substantial heterogeneity and key limitations in current approaches, including lack of granularity in coded data, workflow barriers in clinician-entered systems, and underuse of scalable AI methods, underscoring the need for standardised definitions and improved hybrid data-driven tools to enable more accurate and sustainable monitoring of antibiotic use in stewardship programmes.
- [Uren HD, Aliieva N, Jensen MPN, Matolinets N, Holcomb JB, Fedchyshyn N, et al. Can antimicrobial stewardship programmes be implemented during an armed conflict? A retrospective interventional cohort study. J Antimicrob Chemother. 2026 Jun;81(6):dkag115. doi:10.1093/jac/dkag115](https://ad-id.co/3Psm4q0)
  - Summary: This retrospective before-and-after study evaluated the implementation of an antimicrobial stewardship programme in a civilian hospital within the trauma evacuation pathway in Ukraine during active armed conflict.
- [Weiss ZF, Basu SS. Point-Counterpoint: The “obsoletogram”—are annual hospital antibiograms still relevant? J Clin Microbiol. 2026 May 22; doi:10.1128/jcm.00476-26](https://ad-id.co/3Psm4Gw)
  - Summary: This perspective article discusses the role and evolving relevance of annual hospital antibiograms, while also addressing growing debate over their limitations in the context of modern precision medicine and rapid diagnostic technologies that may offer more individualized and timely resistance information.

**Infection Prevention / Antibiotic Prophylaxis**

- [Alzyood M, Veldhuis A, Stevenson H, Sheikh S. Hidden failure modes of large language models in healthcare-associated infection surveillance: a structured evaluation using NHSN definitions. Infect Control Hosp Epidemiol. 2026;47(6):568-73. doi:10.1017/ice.2026.10444](https://ad-id.co/3Psm4X2)
  - Summary: This study evaluated GPT-5.1 Thinking for classifying healthcare-associated infections using National Healthcare Safety Network (NHSN) definitions across 70 synthetic clinical vignettes and found that accuracy improved substantially with increasingly structured prompting (78.6% to 95.7%), particularly for infections with clearer diagnostic criteria such as surgical site infections and ventilator-associated pneumonia, but performance remained limited for complex definitions involving exclusion rules like CLABSI and CDI due to systematic errors including prioritization of clinical plausibility over surveillance rules, failure to apply temporal or quantitative thresholds, and incorrect hierarchical attribution; overall, the findings suggest that while large language models may assist infection surveillance under constrained, supervised settings, they are not yet reliable for autonomous NHSN classification and require further development and validation before clinical deployment.

**Bloodstream Infections and Endocarditis**

- [Timbrook TT, Neuner EA. How likely is non-inferiority? A Bayesian reappraisal of 7-day antibiotic trials in bacteremia. J Infect Public Health. 2026 May 21; doi:10.1016/j.jiph.2026.103266](https://ad-id.co/4nZfV1c)
  - Summary: This letter presents a Bayesian reanalysis of a prior systematic review and meta-analysis comparing 7- versus 14-day antibiotic therapy for Gram-negative bloodstream infections, using probabilistic noninferiority thresholds and sensitivity analyses with neutral and informative priors, and finds that shorter treatment durations are likely noninferior across multiple outcomes including 90-day mortality, relapse, acute kidney injury, diarrhea, and allergic reactions, with probabilities of noninferiority generally exceeding 66% and reaching up to 94% for mortality. Overall, the analysis supports shorter antibiotic courses as a generally safe and potentially beneficial antimicrobial stewardship strategy while acknowledging residual uncertainty in recurrence risk.

**Gastrointestinal Tract Infections**

- [Talan DA, Moran GJ, Machado-Aranda D, Chiang WK, Faine BA, Fleischman R, et al. Nonoperative treatment of appendicitis and implications for emergency department management: a narrative review. Ann Emerg Med. 2026 Jun;87(6):735-45. doi:10.1016/j.annemergmed.2025.09.035](https://ad-id.co/4nRrwPG)
  - Summary: This review summarizes evolving evidence challenging the traditional view of appendicitis as a surgical emergency, highlighting that modern imaging distinguishes uncomplicated from perforated disease and that uncomplicated appendicitis may often be infection-driven and self-limiting, with multiple multicenter trials involving over 4,000 adults and children demonstrating that nonoperative management using antibiotics and observation with selective surgery is a safe and effective alternative to urgent appendectomy; these findings have led the American College of Surgeons to endorse nonoperative treatment and suggest that most patients (up to 90%) may be safely managed in outpatient settings, with important implications for emergency care pathways and treatment de-escalation strategies.
- [Verma N, Mehtani R, Valsan A, Garg P, Nadda RK, Prasad S, et al. Epidemiology, predictors of antimicrobial resistance and empiric treatment strategies for spontaneous bacterial peritonitis in cirrhosis: a multicentre SBP-INDIA study. Aliment Pharmacol Ther. 2026 May 13; doi:10.1111/apt.70716](https://ad-id.co/3Psm5dy)
  - Summary: This multicentre study of 319 hospitalized patients with cirrhosis and culture-positive ascitic fluid infections in India found a high prevalence of multidrug-resistant (53.6%) and carbapenem-resistant organisms (24.1%), predominantly Gram-negative bacteria - MDRO infection was associated with worse survival, underscoring that empiric therapy should be risk-stratified based on clinical setting and severity to improve antimicrobial stewardship and outcomes.
- [Cadoli C, Müller V, Welsch C, Mühl H, Trebicka J, Kempf VAJ. Role of Gram-negative bacterial infections in acute-on-chronic liver failure. Liver Int. 2026 Jul;46(7):e70710. doi:10.1111/liv.70710](https://ad-id.co/43I1Rje)
  - Summary: This review examines the role of bacteria, bacterial metabolites, and antimicrobial resistance in the pathogenesis and progression of acute-on-chronic liver failure (ACLF), highlighting how Gram-negative bacterial translocation from the gut and infections such as peritonitis, pneumonia, urinary tract infections, and skin infections contribute to systemic inflammation, acute decompensation of cirrhosis, multi-organ failure, and high mortality, while also emphasizing that rising multidrug resistance limits antimicrobial treatment options and that although preventive strategies show some promise, important limitations remain.
- [Olaru-Stavila C, Steinmann SM, Mester P, Müller M, Tcaciuc E, Gülow K. The gastrointestinal barrier—mechanisms of barrier dysfunction in liver cirrhosis and spontaneous bacterial peritonitis. Biomedicines. 2026 May;14(5):1084. doi:10.3390/biomedicines14051084](https://ad-id.co/4u4F4ZH)
  - Summary: This review describes how gastrointestinal (GI) barrier dysfunction in liver cirrhosis arises from a combination of disrupted epithelial tight junctions, impaired mucus layer integrity, microbial dysbiosis with loss of beneficial short-chain fatty acid–producing bacteria and expansion of pathobionts, cirrhosis-associated immune dysfunction, and gut–vascular barrier breakdown, all of which synergistically promote bacterial translocation and drive spontaneous bacterial peritonitis (SBP), systemic inflammation, circulatory dysfunction, and acute decompensation, while also highlighting emerging mechanistic insights such as bile acid–FXR signalling and microbiome-derived metabolites that may offer future therapeutic targets beyond current antibiotic-based and supportive management.

**CNS Infections**

- [Waldrop G, Reddy SP. Metagenomic next-generation sequencing in central nervous system infections: clinical strategies, evidence, and best practices. Clin Infect Dis. 2026 May 15;82(Suppl 4):S92-S99. doi:10.1093/cid/ciag120](https://ad-id.co/3PyvQH7)
  - Summary: This guidance article discusses the clinical application and interpretation of cerebrospinal fluid metagenomic next-generation sequencing (mNGS) for suspected central nervous system infections, emphasizing its shift from targeted molecular diagnostics to an agnostic, broad-spectrum approach that improves pathogen detection especially in atypical and immunocompromised cases. Although mNGS is described as a transformative tool capable of narrowing the diagnostic gap in CNS infections and aiding clinical decision-making, the authors stress that its utility is limited by cost, turnaround time, and the need for careful multidisciplinary interpretation, and it should complement rather than replace clinical judgment.

**Respiratory Tract Infections**

- [Cilloniz C, Pericàs JM, Miró JM, Torres A. Pulmonary infections in patients with human immunodeficiency virus infection. Semin Respir Crit Care Med. 2026;47(2):204-214. doi:10.1055/a-2725-7350](https://ad-id.co/4u8fdzY)
  - Summary: This review summarizes the current evidence on pulmonary infections in people living with HIV, emphasizing that respiratory infections remain a major cause of hospitalization, morbidity, and mortality particularly in individuals with advanced immunosuppression, with pneumonia due to pathogens such as Pneumocystis jirovecii and Mycobacterium tuberculosis being most common, while also highlighting the diagnostic challenges posed by overlapping clinical presentations and frequent co-infections, and discussing updated approaches to epidemiology, clinical features, diagnosis, and management in the context of antiretroviral therapy.
- [Pitrowsky M, Pacheco MB, Ranzani O, Amado FS, Nassar Junior AP, Besen BAM, et al. Outcomes of pneumonia in ICUs in low- and middle-income countries: a systematic review. N Engl J Med Evid. 2026 Jun;5(6):EVIDoa2500244. doi:10.1056/EVIDoa2500244](https://ad-id.co/4x0ipjQ)
  - Summary: This systematic review and meta-analysis of 52 studies involving 48,707 patients across 18 countries estimated that community-acquired pneumonia (CAP) in ICU patients in low- and middle-income settings is associated with high mortality (pooled ICU/28–30-day mortality 37%), rising substantially to 61% among those requiring mechanical ventilation, with age and mechanical ventilation identified as the strongest predictors of mortality explaining over half of between-study heterogeneity, while also highlighting that most available data come from upper-middle-income countries and that there is a notable absence of evidence from low-income countries.
  - **Editorial Commentary:**
    [Helms L, Bebell LM. Severe pneumonia in low- and middle-income countries—a gap we can close. N Engl J Med Evid. 2026 Jun;5(6):EVIDe2600111. doi:10.1056/EVIDe2600111](https://ad-id.co/4ehlh4v)

**Urinary Tract Infections**

- [Beaumont AL, Danjean M, Jacquier H, Boyd A, Jaspard M, Royer G, et al. Dissemination of ESBL-producing *Escherichia coli* and carriage in the community worldwide: epidemiology, transmission pathways, molecular insights, and practical aspects. Clin Microbiol Rev. 2026 May 27; doi:10.1128/cmr.00274-25](https://ad-id.co/3Psm5u4)
  - Summary: This review examines global differences in community carriage of extended-spectrum β-lactamase-producing Escherichia coli (ESBL-Ec) since their worldwide spread in the early 2000s, highlighting lower prevalence in high-income countries compared with higher and more concerning rates in low- and middle-income countries, and attributing these disparities to a complex interplay of socioeconomic factors (such as income and Human Development Index), differing transmission dynamics (predominantly human–animal–environmental transmission in LMICs versus mainly inter-human spread in HICs), and distinct bacterial population structures (with LMICs enriched for phylogroups A and B1 and HICs for phylogroup B2); overall, the authors emphasize that ESBL-Ec dissemination is multifactorial and requires tailored public health interventions, including strengthened antimicrobial stewardship and context-specific control strategies to curb further global spread.

**Infections in Children**

- [Tenforde MW, Ujamaa D, Talbot HK, O’Halloran AC, Chai SJ, Sachdev D, et al. Prevalence and outcomes of bacterial co-detections by blood culture among children and adults hospitalized with laboratory-confirmed influenza, Influenza Hospitalization Surveillance Network, 2022–2024. Clin Infect Dis. 2026 May 21; doi:10.1093/cid/ciag242](https://ad-id.co/4nSIRI4)
  - Summary: This large US population-based FluSurv-NET surveillance study of 14,316 patients hospitalized with laboratory-confirmed influenza across the 2022–2023 and 2023–2024 seasons found that bacterial blood culture co-detections occurred in 5.2% of patients and were associated with progressively worse outcomes, including higher ICU admission rates and in-hospital mortality that increased from 1.5% in patients without cultures to 14.5% in those with multiple positive cultures; among identified pathogens, Staphylococcus aureus and Streptococcus pneumoniae were the most common and were each associated with substantial severity and approximately 22% mortality, underscoring the significant clinical burden of influenza-associated bacterial co-infections and supporting the importance of preventive strategies such as vaccination to reduce morbidity and mortality.
- [O’Leary ST, Danchin M. Childhood vaccine hesitancy. N Engl J Med. 2026 May 20; doi:10.1056/NEJMcp2516616](https://ad-id.co/3Psm616)
  - Summary: This review discusses vaccine hesitancy along a spectrum in which most concerned parents remain motivated to protect their children but are primarily driven by safety concerns.
- [Msanga DR, Minja CA, Härtel C, Claus H, Mshana SE, Kurzai O, et al. Epidemiology, resistance and virulence of neonatal *Klebsiella pneumoniae* isolates from a tertiary hospital in Tanzania. J Infect Dis. 2026 May 11; doi:10.1093/infdis/jiag253](https://ad-id.co/4vgTE1g)
  - Summary: This retrospective molecular epidemiology study of 41 Klebsiella pneumoniae isolates from neonates with bloodstream infections at Bugando Medical Centre in Mwanza, Tanzania, demonstrated high levels of resistance to penicillins, cephalosporins, and aminoglycosides with frequent ESBL production, while carbapenems remained largely active (95.1% susceptibility). Phylogenetic clustering revealed multiple transmission events within the hospital and possible gut-to-bloodstream spread, highlighting significant nosocomial transmission dynamics and reinforcing the need for strengthened infection control and antimicrobial stewardship in neonatal care settings in low-resource environments.
- [Andersen M, Matthiesen NB, Murra M, Nielsen SY, Henriksen TB. Early-onset neonatal infection and cerebral palsy. JAMA Netw Open. 2026 May 26;9(5):e2614775. doi:10.1001/jamanetworkopen.2026.14775](https://ad-id.co/4o43BwM)
  - Summary: This nationwide Danish cohort study of 1,034,416 near-term and full-term singleton births found that early-onset neonatal invasive bacterial infections were strongly associated with later development of cerebral palsy, with 0.8% experiencing sepsis and <0.1% meningitis, and demonstrating markedly increased adjusted odds of cerebral palsy in children with sepsis (OR 8.13), particularly culture-positive cases (OR 23.58), and even higher risks with meningitis (OR up to 79.57), with sepsis also more strongly linked to severe cerebral palsy phenotypes such as nonambulatory status and bilateral involvement, highlighting early neonatal infection as a major potentially preventable risk factor for long-term neurodevelopmental disability.
- [Dionisopoulos Z, Sabhaney V, D’Arienzo D, Fung A, Dent A, Cunningham J, et al. Prevalence of invasive bacterial infections among febrile infants aged 60 to 90 days: a systematic review and meta-analysis. JAMA Pediatr. 2026 May 26; doi:10.1001/jamapediatrics.2026.1815](https://ad-id.co/3Psm6hC)
  - Summary: This systematic review and meta-analysis of 20 cohorts including 34,835 well-appearing febrile infants aged 60–90 days evaluated in emergency and outpatient settings found a pooled prevalence of invasive bacterial infection of 1.11%, with bacteremia occurring in 1.01% and bacterial meningitis in 0.11%, providing contemporary risk estimates to inform clinical guidelines and shared decision-making between clinicians and parents regarding evaluation and management of fever in young infants.
- [Castejon-Ramirez S, Hijano DR, Wattier RL, Ferrolino J, Peterson M, Merritt P, et al. Early discontinuation of empiric antibiotics in pediatric haploidentical hematopoietic cell transplant recipients with febrile neutropenia. Clin Infect Dis. 2026 May 26; doi:10.1093/infdis/ciag327](https://ad-id.co/4dT78ZW)
  - Summary: This retrospective quasi-experimental study of children, adolescents, and young adults undergoing haploidentical hematopoietic cell transplantation evaluated a 2017 guideline recommending discontinuation of empiric antibiotics within 72 hours of febrile neutropenia if no infection was identified, and found that implementation of the policy significantly reduced antibiotic days of therapy by a median of 8 days without increasing bloodstream infection rates, ICU admission, or mortality, while DOOR-modified RADAR analysis showed a 70% probability of improved overall clinical outcomes in the post-implementation group; overall, the findings suggest that early antibiotic discontinuation in this high-risk population is safe, reduces antibiotic exposure, and supports antimicrobial stewardship, although confirmation in prospective multicentre trials is needed.
  - **Editorial Commentary:**
    [Song E, Ardura MI. What if Goldilocks had a choice? Rethinking the optimal duration of empiric antibiotics in pediatric hematopoietic cell transplant recipients with persistent fever and neutropenia. Clin Infect Dis. 2026 May 26; doi:10.1093/cid/ciag329](https://ad-id.co/4nTdb5k)

**Mycobacterial Infections**

- [Brown TS, Nelson K, Kissler S, Martinez L, Horsburgh CR, White LF, et al. Understanding and exploiting superspreading to disrupt Mycobacterium tuberculosis transmission. Lancet Infect Dis. 2026 May 14; doi:10.1016/S1473-3099(26)00168-4](https://ad-id.co/3PKcFKw)
  - Summary: This Personal View discusses tuberculosis transmission dynamics with a focus on individual heterogeneity in infectiousness and the concept of Mycobacterium tuberculosis superspreading, in which a small proportion of highly infectious individuals account for most secondary infections and clinical cases, and argues that this widely observed epidemiological pattern is supported by both historical and contemporary data and represents a critical opportunity for targeted public health interventions that could disproportionately reduce transmission and accelerate progress toward tuberculosis elimination by interrupting spread from the most infectious individuals.
- [Chihota V, Waggie Z, Cardenas V, Mngadi K, Ndebele F, Brumskine W, et al. Safety of short-course weekly rifapentine and isoniazid (3HP) for TB preventive treatment at conception and during first trimester in women living with HIV: a secondary analysis of WHIP3TB trial. Clin Infect Dis. 2026 May 17; doi:10.1093/cid/ciag315](https://ad-id.co/4nVT3iU)
  - Summary: This analysis of pregnancy outcomes within the WHIP3TB trial evaluated HIV-positive pregnant women receiving tuberculosis preventive therapy with once-weekly rifapentine–isoniazid for 3 months (3HP) or 6 months of isoniazid, and found that among 236 pregnancies in 222 participants, including 57 with first-trimester study drug exposure, rates of spontaneous abortion and a composite adverse pregnancy outcome (stillbirth, spontaneous abortion, low birth weight, preterm delivery, or congenital anomalies) were similar between exposed and unexposed groups (approximately 33–35%), with no evidence of increased harm associated with 3HP exposure during early pregnancy, thereby supporting its safety for tuberculosis prevention in this population.
- [Bandyopadhyay R, Manesh A, Kundu D, George MM, Nagaraj V, Varghese GM. Anti-IFN-γ autoantibodies and disseminated opportunistic infections in immunocompetent Indian adults. Int J Infect Dis. 2026 May 20; doi:10.1016/j.ijid.2026.108806](https://ad-id.co/4fxzQlR)
  - Summary: This cross-sectional study compared HIV-negative, apparently immunocompetent patients with disseminated intracellular opportunistic infections (n=44) or persistent tuberculosis (n=41) against healthy controls (n=34) and found significantly higher plasma anti-interferon-γ autoantibody levels in both patient groups, which were associated with severe opportunistic infections such as disseminated nontuberculous mycobacterial disease, cryptococcosis, and histoplasmosis as well as persistent tuberculosis, while elevated IL-12 levels were observed only in patients with disseminated intracellular infections; overall, the findings support anti-IFN-γ autoantibodies as a key immunological marker linked to adult-onset immunodeficiency phenotypes and severe opportunistic infections, highlighting their potential role in explaining disseminated infections in otherwise immunocompetent individuals.
- [Chen Y, Liang J, Liu D, Xu P, Jiang Q, Yang T, et al. Long-term spatiotemporal evolution of drug-resistant *Mycobacterium tuberculosis* in China. Nat Commun. 2026 May 27; doi:10.1038/s41467-026-73577-0](https://ad-id.co/4fe6XLh)
  - Summary: This large-scale genomic study analyzing over 18,000 Mycobacterium tuberculosis genomes across China reconstructed the temporal and spatial evolution of drug resistance and found that resistance-conferring mutations to multiple antibiotics have repeatedly emerged, giving rise to hundreds to over a thousand small drug-resistant clades that have largely arisen within the past two decades, with transmission predominantly occurring within provinces but occasionally spreading geographically in association with human mobility and bacterial fitness; integration of genomic and CRISPRi screening data further identified mutations linked to the emergence and dissemination of resistance, highlighting the combined roles of bacterial adaptation and population movement in shaping local and regional tuberculosis epidemics in China.

**Fungal Infections and Antifungal Agents**

- [Wehbe E, Sreeharan T, Sutrave G, Bui J, Lau C, Park JY, et al. Voriconazole therapy and CYP2C19 phenotype: identifying patients who may need alternative antifungal therapy. J Antimicrob Chemother. 2026 Jun;81(6):dkag168. doi:10.1093/jac/dkag168](https://ad-id.co/4ofzWAV)
  - Summary: This multicentre retrospective study of 194 patients receiving voriconazole across three Australian hospitals evaluated whether CYP2C19 metabolizer phenotype influenced clinical switching to alternative antifungal therapy and found that, despite known pharmacogenomic effects on voriconazole exposure, switching decisions were not associated with CYP2C19 phenotype but were more strongly linked to adverse effects and higher inflammatory status (CRP), while pharmacokinetic analysis showed that all ultrarapid metabolizers on standard dosing had subtherapeutic drug levels unless doses were increased; overall, the findings suggest that voriconazole prescribing decisions are multifactorial and support a combined approach of pharmacogenomic testing to identify patients requiring dose adjustments alongside therapeutic drug monitoring to optimize exposure.
- [Standl L, Huber T, Bloos F, Thomas-Rüddel D, Träger J, Kluge S, et al. Diagnostic performance of β-(1→3)-D-glucan, two Candida antigen, and five anti-Candida antibody assays in ICU patients with sepsis and high risk for invasive candidiasis: a secondary endpoint of the CandiSep randomized clinical trial. J Clin Microbiol. 2026 May;64(5):e01500-25. doi:10.1128/jcm.01500-25](https://ad-id.co/4dSAKXC)
  - Summary: This analysis of sera from the CandiSep multicentre randomized trial involving 342 sepsis patients at high risk for invasive Candida infection found that 14.0% developed invasive candidiasis and 4.1% candidemia, and demonstrated that antigen-based biomarkers (β-(1→3)-D-glucan, Platelia mannan, and Serion mannan) were significantly elevated in infected patients whereas anti-Candida antibody levels were largely influenced by colonization rather than invasive disease; however, all assays showed suboptimal sensitivity and specificity at manufacturer cutoffs, with improved but still limited performance after recalibration (e.g., BDG sensitivity 46% for ICI and 64% for candidemia at 80% specificity), no significant differences between antigen tests, and no added diagnostic benefit from combining biomarkers, leading to the conclusion that while antigen assays have moderate utility for diagnosing invasive candidiasis in critically ill patients, antibody assays are of limited value and overall biomarker performance requires optimized thresholds rather than multiplexing strategies.
- [Feser M, Maphosa T, Shroufi A, Rangaraj A, Talbot VR, Songane M, et al. Clinical impact and cost-effectiveness of improving access to cryptococcal meningitis diagnostics and treatment in Malawi. Clin Infect Dis. 2026 May 28; doi:10.1093/infdis/ciag270](https://ad-id.co/3RTDexn)
  - Summary: This cost-effectiveness modelling study using the CEPAC-I simulation framework evaluated strategies to improve cryptococcal meningitis (CM) outcomes among people living with HIV initiating care in Malawi with positive serum cryptococcal antigen testing and found that expanding access to optimal CM therapy (liposomal amphotericin B, flucytosine, and fluconazole) and enhancing diagnostic pathways (including semi-quantitative CrAg testing with confirmatory lumbar puncture for asymptomatic CrAg-positive individuals) modestly increased 1-year survival (up to +6.0%) and quality-adjusted life years while remaining highly cost-effective (ICERs US$140–380/QALY), demonstrating that both improved treatment access and earlier detection of asymptomatic CM are economically and clinically beneficial in high-burden, resource-limited settings.
- [Tan RYM, Chen ZX, Sivaraman J, Kong KW, Tan Z, Xu S, et al. Ubiquitination defect of XIAP as novel susceptibility to invasive fungal pneumonia. J Infect Dis. 2026 May 26; doi:10.1093/infdis/jiag272](https://ad-id.co/4u7cqas)
  - Summary: This study demonstrates that hypomorphic mutations in the X-linked Inhibitor of Apoptosis Protein (XIAP) gene lead to dysregulated ubiquitination and enhanced apoptosis, resulting in impaired innate immune function and increased susceptibility to invasive fungal pneumonia, thereby highlighting the critical role of programmed cell death and protein ubiquitination pathways in maintaining immune homeostasis and antifungal defence.

**Diagnostics**

- [Bhatnagar AS, Harris H, Jacobs E, Bumpus-White P, Balbuena R, Stambaugh H, et al. Comparison of laboratory-developed methods for aztreonam plus ceftazidime-avibactam antimicrobial susceptibility testing for metallo-β-lactamase-producing Enterobacterales. J Clin Microbiol. 2026 May 22; doi:10.1128/jcm.01800-25](https://ad-id.co/4feTJhk)
  - Summary: This multicentre evaluation of four laboratory-developed phenotypic methods for assessing aztreonam-based combination susceptibility in 102 metallo-β-lactamase-producing Enterobacterales compared performance across two institutions against reference broth microdilution and found substantial variability in accuracy and reproducibility depending on method, reagent, and media manufacturer, with broth disk elution achieving the highest and most consistent performance (>90% categorical agreement and reproducibility), while disk stacking and other strip-based approaches showed lower and more variable agreement; overall, the study highlights significant methodological heterogeneity in LDT-based testing for aztreonam-avibactam combinations and underscores the need for improved standardisation and harmonisation to ensure reliable clinical susceptibility results in the absence of FDA-cleared testing platforms.
- [Samuelsen Ø, López-Causapé C, Aarestrup FM, Bortolaia V, Brouwer MSM, Cantón R, et al. The role of whole genome sequencing in antimicrobial susceptibility prediction of bacteria: 2025 update from the EUCAST Subcommittee. Clin Microbiol Infect. 2026 May 15; doi:10.1016/j.cmi.2026.05.012](https://ad-id.co/4nUZZx1)
  - Summary: Recent EUCAST review highlights major advances in whole genome sequencing–based antimicrobial susceptibility prediction, particularly for Mycobacterium tuberculosis, but concludes that it is still not ready for routine clinical decision-making due to gaps in validation, standardization, and resistance mechanism coverage; it calls for integrated phenotypic-genotypic datasets, harmonized bioinformatics frameworks, and stronger external quality assurance to enable future clinical adoption.
- [Rotundo S, Russo A, Morena R, Garofalo E, Morrone HL, Mazza G, et al. Early targeted therapy guided by rapid phenotypic antimicrobial susceptibility testing in critically ill patients with Gram-negative bacterial bloodstream infections: a retrospective cohort study. J Antimicrob Chemother. 2026 Mar;81(3):dkag056. doi:10.1093/jac/dkag056](https://ad-id.co/3PQ0bkj)
  - Summary: In a retrospective study of critically ill patients with Gram-negative bloodstream infections, rapid antimicrobial susceptibility testing (RAST) was associated with earlier initiation of active therapy, reduced clinical failure, and significantly lower 30-day mortality compared with conventional susceptibility testing. Observational studies such as these may be subject to many biases and confounders - to suggest that RAST may improve survival outcomes in high MDR settings by enabling faster optimization of antibiotic treatment requires robust RCTs.
- [Govender KN, Street TL, Sanderson ND, Leach L, Morgan M, Eyre DW. Rapid diagnosis of common, undetected, and uncultivable bloodstream infections from positive blood cultures using Oxford Nanopore sequencing: a metagenomic pipeline analysis. Lancet Microbe. 2026 May 14; doi:10.1016/j.lanmic.2025.101333](https://ad-id.co/4dBpJuN)
  - Summary: A nanopore sequencing–based metagenomic workflow applied directly from positive blood cultures enabled rapid pathogen identification (within ~3.3 hours) and earlier antimicrobial resistance prediction (≈20 hours faster than standard testing), achieving high overall accuracy versus conventional diagnostics and demonstrating strong potential for faster, more comprehensive bloodstream infection management, although species-dependent variability in AMR prediction and the need for further validation limit immediate clinical adoption.

**Improving Clinical Research**

- [Albin O, Dickson RP, Rao K, Wunderink RG, Pogue J, Kaye KS. Derivation and preliminary validation of novel hierarchical composite endpoints for severe pneumonia clinical trials. Am J Respir Crit Care Med. 2026 May;212(Suppl 1):aamag162.4537. doi:10.1093/ajrccm/aamag162.4537](https://ad-id.co/4dSKPUr)
  - Summary: This study derived and validated novel hierarchical composite endpoints (HCEs) for severe pneumonia trials using clinician preference–informed frameworks (standard DOOR, partial credit DOOR, and Win Ratio) that integrate mortality, ventilation duration, discharge status, antibiotic toxicity, clinical cure, and recurrence, and applied them to the OVERCOME randomized trial comparing colistin monotherapy versus colistin plus carbapenem for extensively drug-resistant gram-negative infections, where conventional endpoints (28-day mortality and clinical failure) showed no significant differences but the Win Ratio analysis indicated a significant benefit for combination therapy, suggesting that HCEs may provide more sensitive and clinically informative interpretations of pneumonia trial outcomes.
- [Harrison LJ, Chow FC. Proportional odds or win probability as methods for assessing ordinal outcomes in infectious disease clinical trials. J Infect Dis. 2026 May 18; doi:10.1093/infdis/jiag193](https://ad-id.co/4dMiL4R)
  - Summary: This review discusses the use of ordinal outcomes in infectious disease trials and compares two key summary statistics—the proportional odds ratio, which estimates how much more likely treated participants are to fall into better outcome categories than controls, and the win probability, which quantifies the likelihood that a treated individual outperforms a control in a random pairwise comparison—while also introducing stacked-bar charts and bubble plots as visual tools to improve the interpretability and accessibility of ordinal trial results.
- [Kaddaj D, Baas S, Tang EYN, Robertson DS, Pin L, Villar SS. Thompson, Ulam, or Gauss? Multi-criteria recommendations for posterior probability computation methods in Bayesian response-adaptive trials with binary endpoints. Comput Stat Data Anal. 2026 Nov;223:108401. doi:10.1016/j.csda.2026.108401](https://ad-id.co/4nWJ1hv)
  - Summary: This study develops a novel exact algorithm for efficiently computing posterior probabilities in Bayesian response-adaptive randomisation (BRAR) clinical trials with binary endpoints and uses it to benchmark common approximation methods, showing that exact computation is often faster and more reliable for small to moderate numbers of treatment arms, while widely used Gaussian and other approximations can lead to reduced power and inflated type I error rates, and it further provides a practical framework to guide when to use exact versus approximate methods to balance computational efficiency, accuracy, and patient benefit in adaptive trial design.
- [Antola S, Gallone S, Murgia Y, Giacobbe DR, Bassetti M, Giacomini M. A unified database for a set of clinical studies on the treatment of bacterial and fungal infections within the MULTI-SITA project. Stud Health Technol Inform. 2026;336:1880-4. doi:10.3233/SHTI260564](https://ad-id.co/4fOhCwj)
  - Summary: This paper describes the MULTI-SITA observational pharmacological study across 30 Italian centers and its transition from multiple study-specific databases to a unified SQL Server-based data management system with a modular architecture and Blazor Server web interface, improving data interoperability, scalability, and usability for clinicians while supporting future research studies and enabling potential alignment with the OMOP Common Data Model.
- [Kessels R, Long Y, Spijker R, Schuit E, Hollestelle M, van Lieshout C, et al. A knowledge base of designs and statistical methods for adaptive clinical dose-finding trials. Pharm Stat. 2026 May 19; doi:10.1002/pst.70074](https://ad-id.co/4nWJkJr)
  - Summary: This literature review of 315 methodological papers on adaptive dose-finding clinical trials summarizes the evolving landscape of designs used to identify optimal drug doses for later-phase studies, highlighting a strong oncology focus and recent methodological advances that incorporate both toxicity and efficacy outcomes, subgroup- and combination-therapy dose finding, and delayed responses, while noting a shift from traditional model-based approaches such as the Continual Reassessment Method (CRM) toward model-assisted and interval-based designs like Toxicity Probability Interval (TPI) and Bayesian Optimal Interval (BOIN), and it further proposes an interactive classification map to help researchers navigate and select appropriate dose-finding methodologies amid the growing diversity of available approaches.

**Company Announcements**

- [Pfizer Inc. Pfizer advances pivotal pediatric pneumococcal vaccine program following strong positive phase 2 results. 2026 May 20. Available from: https://www.pfizer.com/news/press-release/press-release-detail/pfizer-advances-pivotal-pediatric-pneumococcal-vaccine](https://ad-id.co/3PuqJYv)
  - Summary: Pfizer reported Phase 2 results for its investigational 25-valent pneumococcal conjugate vaccine (25vPnC) in infants, showing strong immunogenicity across all 25 serotypes with particularly marked improvements against serotype 3 (approximately 9-fold higher after dose 3 and 15-fold higher after dose 4 compared with Prevnar 20), along with a safety and tolerability profile consistent with existing pneumococcal vaccines and no new safety concerns, supporting advancement into a pivotal Phase 3 pediatric program aiming to expand protection to up to 90% of disease-causing serotypes in young children while also noting Pfizer’s broader strategy shift toward a next-generation 35-valent adult pneumococcal vaccine candidate entering development by 2026.
- [Gilead Sciences Inc. FDA grants accelerated approval to Gilead’s Hepcludex (bulevirtide-gmod), the first and only approved treatment for chronic hepatitis delta virus (HDV). 2026 May 22. Available from: https://www.gilead.com/news/news-details/2026/fda-grants-accelerated-approval-to-gileads-hepcludex-bulevirtide-gmod-the-first-and-only-approved-treatment-for-chronic-hepatitis-delta-virus-hdv](https://ad-id.co/4o1vllI)
  - Summary: Gilead Sciences announced that the FDA has granted accelerated approval for Hepcludex (bulevirtide-gmod) 8.5 mg, making it the first and only approved treatment in the United States for chronic hepatitis delta virus (HDV) infection in adults, based on Phase 3 MYR301 trial data showing statistically significant reductions in HDV RNA and ALT normalization versus delayed treatment, with sustained virologic and biochemical responses and generally good tolerability; however, clinical outcome benefits remain unproven and continued approval will depend on confirmatory trials, marking a major milestone for a severe and high-risk liver disease with substantial unmet medical need.
- [GlaxoSmithKline plc. Bepirovirsen achieves unprecedented functional cure rates with potential to redefine treatment for chronic hepatitis B. 2026 May 28. Available from: https://www.gsk.com/en-gb/media/press-releases/bepirovirsen-achieves-unprecedented-functional-cure-rates-with-potential-to-redefine-treatment-for-chronic-hepatitis-b](https://ad-id.co/4dWoZiP)
  - Summary: GSK announced positive pivotal Phase III B-Well 1 and B-Well 2 trial results for bepirovirsen, an investigational antisense oligonucleotide for chronic hepatitis B, showing a statistically significant 19% functional cure rate in the overall population and 26% in patients with lower baseline viral activity compared with 0% on standard of care alone, alongside exploratory findings that 49% of treated patients achieved low hepatitis B surface antigen levels (≤100 IU/mL) and meaningful sustained viral suppression after treatment cessation, with an acceptable safety profile and no new safety concerns, supporting its potential as the first therapy to meaningfully increase functional cure rates in a disease affecting over 240 million people worldwide and currently requiring lifelong antiviral therapy in most patients. This announcement coincided with a publication on the trial results in NEJM (see below).
- [Hou J, Lim SG, Buti M, Yuen MF, Gane E, Lampertico P, et al. Phase 3 results of bepirovirsen treatment for chronic hepatitis B virus infection. N Engl J Med. 2026 May 28; doi:10.1056/NEJMoa2515131](https://ad-id.co/4nX2UVJ)
  - Summary: In two replicate phase III randomized, double-blind B-Well trials involving adults with noncirrhotic chronic hepatitis B receiving stable nucleos(t)ide analogue therapy, 24 weeks of bepirovirsen treatment followed by treatment cessation led to a significantly higher functional cure rate at week 72 compared with placebo (20% vs 0% in B-Well 1 and 19% vs 0% in B-Well 2), defined as sustained HBV DNA below the lower limit of quantification and hepatitis B surface antigen loss after stopping therapy, although the treatment was associated with higher rates of adverse events including increased grade 3 events such as alanine aminotransferase elevations.
  - **Editorial Commentary:**
    [Lok AS. A major step toward a cure for hepatitis B infection. N Engl J Med. 2026 May 28; doi:10.1056/NEJMe2605575](https://ad-id.co/4dWbdg1)

**General Interest**

- [Dalovisio JR, Winslow DL. A tribute to George Atkinson Pankey MD, MACP, FIDSA, 1933–2026. J Infect Dis. 2026 May 19; doi:10.1093/infdis/jiag233](https://ad-id.co/42YEXE3)
  - Summary: George Atkinson “Kin” Pankey, MD, a pioneering infectious diseases physician and one of the earliest specialists board certified in infectious diseases in 1972, died at age 92 on 31 March 2026, and is remembered for founding the infectious diseases discipline at Ochsner Clinic in New Orleans in 1963.
- [Laxminarayan R, Limmathurotsakul D, de Abreu AL, Alimi Y, Karkey A, Kanj SS, et al. Meeting the 2024 UN General Assembly declaration targets on antimicrobial resistance. Lancet. 2026 May 19; doi:10.1016/S0140-6736(26)00979-7](https://ad-id.co/4dDssUA)
  - Summary: At the 2024 UN General Assembly high-level meeting on antimicrobial resistance, countries unanimously agreed to global targets including a 10% reduction in bacterial AMR-related mortality by 2030 compared with 2019 levels, an increase in Access-classified antibiotics to at least 70% of total human antibiotic use under the WHO AWaRe framework to promote first-line, lower-resistance treatments, and strengthened efforts to control and reduce antibiotic use in animals as part of a broader global strategy to address antimicrobial resistance.
- [Aronson NE, Musa AM, Satoskar AR. Leishmaniasis. N Engl J Med. 2026;394:2026-39. doi:10.1056/NEJMra2403309](https://ad-id.co/4dN0owD)
  - Summary: Leishmaniasis is discussed with key points including rising cutaneous cases particularly in the Eastern Mediterranean region and a global decline in visceral disease, alongside evolving diagnostic practices toward molecular testing of tissue samples for species identification, limited treatment options now increasingly supplemented by combination therapies for visceral disease, and emerging vaccine candidates currently in preclinical or early human testing stages.
- [Jaber WA, Motairek I, Cho L, Sperry BW. The unpaid infrastructure of cardiovascular science. JACC Adv. 2026 Jun;5(6 Pt 2):102837. doi:10.1016/j.jacadv.2026.102837](https://ad-id.co/4fPwMRT)
  - Summary: Peer review and professional society service in cardiovascular medicine rely heavily on unpaid physician volunteer labor, with millions of peer reviews performed annually representing tens of millions of hours dedicated to evaluating manuscripts and ensuring scientific integrity, forming an essential but often unrecognized foundation behind major advances in cardiology including clinical trials, imaging innovations, and device therapies. The same could be said of infectious diseases..
- [Hojat LS, Furin JJ, Hudda NM, Bonomo RA. Antimicrobial resistance and climate change: interlinked crises demanding global response. In: Henao-Martínez AF, editor. Global Health, Climate Change, and Emerging Infectious Diseases. Cham: Springer; 2026. p. 295-327. doi:10.1007/978-3-032-21237-5_16](https://ad-id.co/3PSEccx)
  - Summary: This article argues that climate change and antimicrobial resistance (AMR) should be viewed as an interconnected global public health crisis rather than separate issues, as climate change accelerates AMR through mechanisms such as increased pathogen growth, extreme weather impacts, and reduced healthcare access, while AMR in turn affects ecosystems, biodiversity, and industry, with both disproportionately harming socioeconomically vulnerable populations and therefore requiring urgent, equity-focused global action grounded in the One Health framework to promote sustainability, climate justice, and coordinated international accountability to mitigate and potentially reverse these combined threats.
- [Karthikeyan G, Devasenapathy N, Ghosh A, et al. Digoxin in patients with symptomatic rheumatic heart disease: a randomized clinical trial. JAMA. 2026 May 10; doi:10.1001/jama.2026.7335](https://ad-id.co/3RADykI)
  - Summary: In a multicenter, randomized, placebo-controlled trial of 1,769 patients with symptomatic rheumatic heart disease in India, digoxin compared with placebo modestly reduced the composite outcome of all-cause death or new-onset or worsening heart failure over a median 2.1-year follow-up (31.4% vs 35.5%; hazard ratio 0.82), driven mainly by fewer heart failure events rather than mortality, while showing low rates of discontinuation due to suspected toxicity, suggesting a potential therapeutic benefit for symptom and heart failure progression management in this high-burden population.
- [Islam MS, Chughtai AA, Wood JG, Sawleshwarakar S, Muscatello DJ, Seale H. Hantavirus outbreak on a cruise ship in the South Atlantic. Lancet. 2026 May 15; doi:10.1016/S0140-6736(26)00934-7](https://ad-id.co/4dPSAKx)
  - Summary: A suspected hantavirus outbreak involving Andes virus was reported among passengers and crew on a cruise ship traveling from Ushuaia, Argentina, with seven cases (including two confirmed) and three deaths among 147 individuals across multiple countries, suggesting a high case-fatality rate and possible person-to-person transmission in a confined maritime setting, with epidemiologic evidence indicating an incubation-consistent index exposure likely occurring in Argentina followed by secondary transmission onboard, highlighting concerns about rapid spread, multi-generational transmission potential, and the need for strengthened surveillance, infection control, and early detection strategies such as wastewater monitoring in cruise ship environments.
- [Klompas M, Al-Hasan M, Al Mohajer M, Colgrove R, Doron S, File T, et al. Infectious Diseases Society of America (IDSA) position statement: why IDSA did not endorse the community-acquired pneumonia guidelines 2025 update. Am J Respir Crit Care Med. 2026 May;212(5):1064-6. doi:10.1093/ajrccm/aamag065](https://ad-id.co/3RxSU9y)
  - Summary: In a formal correspondence regarding the 2025 American Thoracic Society community-acquired pneumonia guideline update, the Infectious Diseases Society of America (IDSA) explains its decision not to endorse the revision despite agreement with most recommendations, citing disagreement with guidance that supports empiric antibiotic use in certain adults with viral-positive pneumonia—specifically outpatients with comorbidities and inpatients with non-severe disease—due to concerns that such practices may lead to unnecessary antibacterial exposure in viral infections, increasing risks of harm and undermining antimicrobial stewardship at both individual and population levels.
- [Jones BE, Ramirez JA. Reply to the Infectious Diseases Society of America (IDSA) position statement. Am J Respir Crit Care Med. 2026 May;212(5):1067-8. doi:10.1093/ajrccm/aamag074](https://ad-id.co/4dRTxSL)
  - Summary: In response to the Infectious Diseases Society of America’s decision not to endorse updated American Thoracic Society–IDSA community-acquired pneumonia guidelines, the guideline committee explains that its consensus-based recommendations were developed through a multidisciplinary and patient-inclusive process and support empiric antibiotic use in most hospitalized adults with viral-positive non-severe pneumonia and in outpatients with comorbidities due to an estimated ~20% risk of bacterial–viral coinfection, while reserving antibiotic withholding for low-risk outpatients without comorbidities; the authors argue that this reflects a deliberate balance between antimicrobial stewardship and individual patient safety in the context of diagnostic uncertainty, limited outcome data, and differing interpretations of acceptable risk, and they express concern that IDSA’s non-endorsement may hinder future collaborative guideline development.
- [Arias CA. The Maxwell Finland Lecture 2025: antimicrobial resistance at the bedside: translating science into patient outcomes. J Infect Dis. 2026 May 26; doi:10.1093/infdis/jiag273](https://ad-id.co/43GNBqV)
  - Summary: This article discusses antimicrobial resistance (AMR) as a major global public health threat driven by the rapid evolutionary capacity of microorganisms and multifactorial selective pressures, highlighting how rising multidrug-resistant organisms continue to undermine the effectiveness of antibiotics and challenge infectious disease management, while also reflecting on the historical contributions of Dr. Maxwell Finland in shaping modern antimicrobial therapy and emphasizing the increasing relevance of his legacy in the current era of diminishing antibiotic efficacy.

**Target Trial Emulation**

- [Christian-Miller N, Lee KT, Pashun R, Ballut K, Prescott HC, Cascino T, et al. Continuing vs withholding home beta-blockers at admission for suspected infection: a target trial emulation. Crit Care Med. 2026 Jun 9; doi:10.1097/CCM.0000000000007229](https://ad-id.co/4vPmOF2)
  - Summary: This retrospective target trial emulation study of patients admitted from the emergency department with suspected infection found that continuation of chronic beta-blocker therapy within 48 hours of admission was associated with lower 90-day all-cause mortality and shorter hospital length of stay compared with discontinuation, while showing no significant difference in in-hospital mortality, suggesting potential clinical benefit of maintaining beta-blockers during acute infectious hospitalisation and supporting the need for randomized trials to confirm causality.
- [Romo M, LaHood A, Mitnick CD, Rich ML, Trevisi L, Skrahina A, et al. Comparison of three linezolid management strategies for peripheral neuropathy in multidrug- or rifampicin-resistant tuberculosis treatment: a target trial emulation. Clin Infect Dis. 2026 Jun 12; doi:10.1093/cid/ciag361](https://ad-id.co/4fM7Afi)
  - Summary: This target trial emulation using data from the endTB Observational Study of patients with multidrug- or rifampicin-resistant tuberculosis who developed non-severe peripheral neuropathy on linezolid found that immediate or deferred dose modification (dose reduction, interruption, or discontinuation) within 26 weeks of neuropathy onset was not associated with reduced treatment success compared with continuing full-dose linezolid, suggesting that cautious linezolid adjustment to manage neuropathy is unlikely to substantially compromise overall MDR/RR-TB treatment outcomes.

**Trial Protocols / Trial Ideas**

- [Mokrani D, Deconinck L, Peiffer-Smadja N, Thy M, Le Hingrat Q, Duval X, et al. Which trial do we need? Evaluating plasma microbial cell-free DNA in blood culture-negative infective endocarditis. Clin Microbiol Infect. 2026 Jun 8; doi:10.1016/j.cmi.2026.06.004](https://ad-id.co/4a24lwv)
  - Summary: This study outlines a two-phase research strategy to evaluate the diagnostic performance, clinical impact, and cost-effectiveness of plasma microbial cell-free DNA sequencing as a non-invasive method for pathogen identification in blood culture–negative infective endocarditis, a condition associated with delayed diagnosis, limited microbiological confirmation, and suboptimal treatment outcomes.

**Phage Therapy**

- [Ulloa ER, Jaffurs D, Tran MT, Lin C, Tran H, Chan D, et al. Calciphylaxis complicated by NDM-1-producing Pseudomonas aeruginosa: first experience with cefepime–zidebactam alongside phage therapy in the USA. Lancet Infect Dis. 2026 Jun 9; doi:10.1016/S1473-3099(26)00176-3](https://ad-id.co/3Q12OjF)
  - Summary: This case report describes a young woman with acute myeloid leukaemia who developed calciphylaxis complicated by superinfection with a pan-resistant New Delhi metallo-β-lactamase-1-producing Pseudomonas aeruginosa, successfully managed using a combination of calciphylaxis-directed therapy, bacteriophage therapy, and the first reported use of cefepime–zidebactam in the USA, highlighting the potential of innovative treatments for rare and highly resistant infections.
- [Classen AY, Clokie MRJ, Giske CG, Nir-Paz R, Pirnay JP, Resch G, et al. Towards a reference methodology for bacteriophage susceptibility testing – position paper of the EUCAST Phage Susceptibility Testing subcommittee. Clin Microbiol Infect. 2026 Jun 13; doi:10.1016/j.cmi.2026.06.005](https://ad-id.co/4fMrVRK)
  - Summary: This perspective argues that while bacteriophage therapy offers a highly targeted and promising approach for treating multidrug-resistant bacterial infections due to its strain-specific activity, its broader clinical adoption is limited by the absence of a standardized, reproducible phage susceptibility testing (PST) framework analogous to antimicrobial susceptibility testing for antibiotics, and emphasizes that establishing such a reference methodology is essential for enabling reliable phage selection, comparability across centers, and meaningful evaluation in clinical trials and outcome correlations.

**PK/PD and Drug Dosing**

- [Pai MP, Cojutti PG, De Paola R, Zamparini E, Tedeschi S, Giuliano S, et al. Optimizing dalbavancin dosing for complicated MRSA infections: a multicenter population pharmacokinetic study. Clin Microbiol Infect. 2026 Jun 8; doi:10.1016/j.cmi.2026.05.049](https://ad-id.co/3QDa1GN)
  - Summary: This multicenter pharmacokinetic modeling study found that obesity and preserved kidney function substantially reduce dalbavancin exposure during prolonged treatment of MRSA infections, and demonstrated that BMI- and eGFR-informed dosing strategies can achieve high probabilities of target attainment for 4–8 weeks of therapy, although effectiveness may be reduced under higher protein-binding assumptions, particularly in high-risk patients.
- [Cree ML, Abdul-Aziz MH, Wallis SC, Won H, Sumi CD, Marjanovic D, et al. The impact of an ex vivo paediatric renal replacement therapy circuit on antimicrobial concentrations. J Antimicrob Chemother. 2026 Jul;81(7):dkag189; doi:10.1093/jac/dkag189](https://ad-id.co/4aOYL0J)
  - Summary: This ex vivo study of a paediatric continuous renal replacement therapy model demonstrated that CRRT significantly reduced concentrations of more than 70% of tested antimicrobials through increased filter clearance and drug-circuit adsorption, highlighting the need to reassess antimicrobial dosing in critically ill children receiving CRRT to ensure therapeutic drug exposure and reduce the risk of treatment failure.
- [Cojutti PG, Toschi A, Pasquini Z, Paolini S, Maffini E, Bonifazi F, et al. Likelihood of aggressive PK/PD target attainment of continuous-infusion beta-lactams during the first week of treatment of febrile neutropenia: findings from a 1-year prospective, monocentric study in onco-haematological patients. J Antimicrob Chemother. 2026 Jul;81(7):dkag183. doi:10.1093/jac/dkag183](https://ad-id.co/43ANspa)
  - Summary: This prospective study of onco-haematological patients with febrile neutropenia found that continuous-infusion beta-lactams achieved aggressive pharmacokinetic/pharmacodynamic targets more reliably during targeted than empirical therapy, with augmented renal clearance emerging as the strongest predictor of underexposure, supporting the use of therapeutic drug monitoring to optimize dosing in this high-risk population.
- [al Jalali V, Aranzana-Climent V, Jorda A, Bergmann F, Lackner E, Wölfl-Duchek M, et al. Prostatic tissue distribution of gepotidacin in patients undergoing prostatectomy: an ex-vivo microdialysis and pharmacokinetic modelling study. Clin Microbiol Infect. 2026 Jun 3; doi:10.1016/j.cmi.2026.05.024](https://ad-id.co/43w75yF)
  - Summary: This study used a novel ex-vivo microdialysis approach and population pharmacokinetic modelling to demonstrate that oral gepotidacin achieves unbound concentrations in human prostate tissue comparable to plasma, supporting its potential use for bacterial prostatitis while highlighting the need to establish pharmacokinetic/pharmacodynamic targets against relevant pathogens to predict clinical efficacy.
- [Matcha S, Rao GG, Hanafin PO, Liu X, Yu Z, Zhang J, et al. Population pharmacokinetics of polymyxin B: an individual participant data meta-analysis. Clin Microbiol Infect. 2026 Jun 1; doi:10.1016/j.cmi.2026.05.036](https://ad-id.co/4aQ0KSu)
  - Summary: This individual participant data meta-analysis of five clinical studies developed a population pharmacokinetic model for polymyxin B, identifying creatinine clearance, sex, and age as significant determinants of drug disposition and supporting a regimen of a 100 mg loading dose followed by 75 mg every 12 hours for non-pulmonary infections, while highlighting reduced target attainment at MICs >1 mg/L and the need to reassess current susceptibility breakpoints.

**Beta-Lactamases and Other Resistance Mechanisms**

- [Ansari UA, Campbell D, Brandenburg JM, Duffy N, Grass JE, Guh AY, et al. Increase in blaNDM among carbapenemase-producing, carbapenem-resistant Enterobacterales, United States, 2016–2023. Emerg Infect Dis. 2026 Jun;32(6):1028-1030. doi:10.3201/eid3206.251404](https://ad-id.co/4v5ItJ2)
  - Summary: Surveillance data from 1,288 carbapenemase-producing carbapenem-resistant Enterobacterales collected across 10 US sites between 2016 and 2023 revealed a marked increase in blaNDM prevalence from 5.4% to 39.8%, accompanied by a decline in blaKPC, raising concerns about the growing spread of highly drug-resistant NDM-producing organisms and the limited treatment options available against metallo-β-lactamase-mediated resistance.
- [Macesic N, Harris PNA, Mo Y, Gomez-Simmonds A. Carbapenemases: epidemiology, detection and management in a changing global landscape. JAC Antimicrob Resist. 2026 Jun;8(3):dlag099. doi:10.1093/jacamr/dlag099](https://ad-id.co/4fHHRoh)
  - Summary: This review examines the global emergence and spread of carbapenemase-producing Gram-negative bacteria, outlining their molecular epidemiology, diagnostic approaches, and mechanism-directed treatment strategies, while highlighting the growing burden of metallo-β-lactamase producers, limited therapeutic options, disparities in access to diagnostics and novel agents, and the critical role of infection prevention and antimicrobial stewardship in controlling resistance.
- [Brunetti F, Marshall SH, Bethel CR, Hujer AM, Hujer KM, Haider S, et al. Standardized numbering and alignment of the KPC family of β-lactamases. Antimicrob Agents Chemother. 2026 May 29; doi:10.1128/aac.01868-25](https://ad-id.co/4v4jN3E)
  - Summary: This study analyzed 267 KPC β-lactamase variants and identified widespread inconsistencies in residue numbering and mutation annotation, particularly among insertion/deletion variants associated with ceftazidime-avibactam resistance, leading to the proposal of a standardized Ambler-based annotation framework to improve consistency in the description and reporting of emerging KPC variants.
- [Abe R, Sugawara Y, Akeda Y, Kerdsin A, Santanirand P, Iida T, et al. Multidimensional prophage profiling of carbapenem-resistant Enterobacteriaceae in Thailand: a nationwide, multicentre, genomic study. Lancet Microbe. 2026 Jun 12; doi:10.1016/j.lanmic.2026.101382](https://ad-id.co/4a3i2LD)
  - Summary: This nationwide genomic study of 747 carbapenem-resistant Enterobacteriaceae isolates from Thailand found extensive diversity in prophage content, with 374 distinct prophage clusters that varied by species, sequence type, clinical specimen, and carbapenemase genotype, and demonstrated that prophage repertoires provide additional epidemiological resolution beyond standard multilocus sequence typing by revealing lineage- and niche-associated patterns, suggesting that integrating prophage profiling into molecular surveillance could improve transmission tracking and infection control.

**Antibiotic Stewardship and Hospital in the Home**

- [Manesh A, Aslan AT, George MM, Sulaiman H, Huttner A, Mathers AJ, et al. Global survey on the management of multidrug-resistant Gram-negative bacilli: divergence of practice between high-income countries and the rest of the world. Clin Infect Dis. 2026 Jun 15;82(Suppl 5):S105-S115. doi:10.1093/cid/ciag025](https://ad-id.co/3QDklP0)
  - Summary: This global survey of 1,320 healthcare professionals from 76 countries found substantial variation in the management of multidrug-resistant Gram-negative bacterial infections, particularly between high-income and low- and middle-income countries, with limited access to newer antimicrobial agents contributing to treatment disparities and highlighting the need to evaluate their impact on outcomes in resource-limited settings.
- [Cidade JP, Póvoa P, Depuydt P, Dhaese S, De Smet K, Tabah A, et al. Impact of appropriate antimicrobial therapy on patient outcomes and antimicrobial use: a sub analysis of the DIANA Study Dataset. Intensive Care Med. 2026 Jun 2; doi:10.1007/s00134-026-08448-9](https://ad-id.co/4vceC1y)
  - Summary: This predefined analysis of the international DIANA cohort found that appropriate empirical antimicrobial therapy was administered to 87.7% of critically ill ICU patients with microbiologically confirmed infections and was independently associated with lower 28-day mortality, as well as more antimicrobial-free and mechanical ventilation-free days, with the greatest survival benefit observed in patients with moderate illness severity.
- [Kim D, Kim YJ, Ryoo SM, Kim WY, Korean Sepsis Alliance (KSA) Investigators. Impact of time to antibiotics on in-hospital mortality in neutropenic sepsis: a prospective multicenter cohort study. Crit Care Med. 2026 Jun 3; doi:10.1097/CCM.0000000000007184](https://ad-id.co/4vdwMQJ)
  - Summary: This prospective multicenter cohort study of 942 patients with neutropenic sepsis found that delayed time to antibiotics was independently associated with increased in-hospital mortality in a dose–response manner, with the strongest harmful effects observed in patients with septic shock and hematologic malignancy, highlighting heterogeneity in the survival impact of antibiotic timing across clinical subgroups.
- [Seibert AM, Hersh AL, Willis P, Nelson B, Bledsoe J, Patel PK, et al. Check engine light, red herring, or MacGuffin? Clinician-level correlations of the antibiotic utilization for respiratory conditions (AXR) metric with outpatient antimicrobial stewardship metrics, diagnostic practices, and prescription characteristics. Clin Infect Dis. 2026 Jun 3; doi:10.1093/cid/ciag255](https://ad-id.co/4v9CvXv)
  - Summary: This retrospective study of 528,444 respiratory urgent care encounters across 32 centers found that higher clinician-level antibiotic utilization for respiratory conditions (AXR) was strongly associated with increased inappropriate antibiotic prescribing and diagnosis rates (including tier 3 antibiotics, treatment despite negative streptococcal testing, and higher sinusitis/otitis media labeling), while being associated with lower use of narrow-spectrum and shorter-duration therapy, supporting AXR as a useful but incomplete screening metric for outpatient antimicrobial stewardship.
  - **Editorial Commentary:**
    [Holubar M, Ha D. Decoding AXR: separating signal from noise in outpatient antibiotic stewardship. Clin Infect Dis. 2026 Jun 3; doi:10.1093/cid/ciag256](https://ad-id.co/3QmNQnZ)
- [Eyre DW, Corrigan R, Hookham L, Lumley S, Clarke D, Jeffery K, et al. An antibiotic chatbot: evaluation of a retrieval-augmented generation approach for providing guideline-based antimicrobial advice. J Infect. 2026 Aug;93:106789. doi:10.1016/j.jinf.2026.106789](https://ad-id.co/3SpMV6V)
  - Summary: This study evaluated a retrieval-augmented generation (RAG) antibiotic chatbot trained on local hospital guidelines and found that a locally constrained large language model could provide mostly accurate infection management advice (81–87% fully correct responses) with improved performance compared with non-grounded models, but still produced occasional errors and inconsistently identified out-of-scope queries, indicating that further optimization and safety safeguards are required before clinical deployment.
- [Bankar P. From principles to practice: learning antimicrobial stewardship through the ESICM fellowship. Intensive Care Med. 2026 Jun 8; doi:10.1007/s00134-026-08509-z](https://ad-id.co/4epO45O)
  - Summary: This narrative describes an ESICM fellowship on sepsis and antimicrobial stewardship in ICU practice, highlighting how multidisciplinary collaboration, bedside stewardship rounds, microbiology integration, pharmacokinetic/pharmacodynamic individualization, and diagnostic stewardship were embedded into routine care, and reflecting how the experience reshaped the author’s clinical approach through structured antibiotic reassessment, improved use of microbiology and biomarkers, and initiation of local stewardship initiatives, reinforcing that effective antimicrobial stewardship in critical care depends on shared responsibility and coordinated team-based decision-making rather than simple antibiotic restriction.

**Infection Prevention / Antibiotic Prophylaxis**

- [Mellinghoff SC, Grossi AA, Recanatini C, Breull-Wierschem L, Salm F, Gadebusch-Bondio M, et al. The human cost of resistance: ethical implications of coping with isolation for multi-drug resistant organisms. Clin Microbiol Infect. 2026 Jun 5; doi:10.1016/j.cmi.2026.05.043](https://ad-id.co/3ScrlTq)
  - Summary: This narrative review examines the clinical and ethical implications of isolating patients colonized or infected with multidrug-resistant organisms, highlighting the tension between infection control benefits and potential harms such as reduced quality of care, treatment delays, and psychosocial burden, and argues for more transparent, proportionate, and evidence-based isolation strategies that balance patient safety with patient wellbeing.
- [Yu Q, Wu Z, Ge Q, Zhang Z, Zhai S. Efficacy and safety of systemic prophylactic antibacterials in mechanically ventilated patients: systematic review and meta-analysis. Crit Care Med. 2026 Jun 1; doi:10.1097/CCM.0000000000007183](https://ad-id.co/4vGY5mi)
  - Summary: This systematic review and meta-analysis of 11 randomized controlled trials involving 5,562 mechanically ventilated ICU patients found that systemic prophylactic antibiotics likely reduce the incidence of ventilator-associated pneumonia, particularly early VAP and in patients with brain injury, but do not reduce in-hospital mortality, duration of mechanical ventilation, or length of stay, with no clear increase in adverse events or antimicrobial resistance reported, though further studies are needed to better define safety and cost-effectiveness.

**Bloodstream Infections and Endocarditis**

- [Bavaro DF, Belati A, Sacchet D, Cilli A, Diella L, Pocaterra D, et al. Anti–methicillin-resistant Staphylococcus aureus (MRSA) cephalosporins plus daptomycin as initial therapy for MRSA bacteremia: does a “hit hard and fast” strategy improve outcomes? Clin Infect Dis. 2026 Jun 9; doi:10.1093/cid/ciag228](https://ad-id.co/4eptlzi)
  - Summary: This multicenter retrospective cohort study of 465 patients with MRSA bloodstream infection found that initial combination therapy with anti–MRSA cephalosporins plus daptomycin was associated with lower 90-day all-cause mortality and a higher probability of overall favourable outcomes compared with other antimicrobial regimens, with consistent results across adjusted Cox regression and propensity-weighted analyses. This combination may be worth studying in an RCT in high-risk patients.
  - **Editorial Commentary:**
    [Doernberg SB, Holland TL. Early combination therapy for MRSA bacteremia: double coverage or double trouble? Clin Infect Dis. 2026 Jun 9; doi:10.1093/cid/ciag229](https://ad-id.co/4vbkRTt)
- [Pogue JM, Rybak MJ, Abdul-Mutakabbir JC, Stamper K, Marchaim D, Thamlikitkul V, et al. The impact of colistin MIC on clinical failure and mortality: insights from the OVERCOME trial. Clin Microbiol Infect. 2026 Jun 5; doi:10.1016/j.cmi.2026.05.046](https://ad-id.co/43zTIgQ)
  - Summary: This post-hoc analysis of the randomized OVERCOME trial evaluated whether colistin minimum inhibitory concentration (MIC) influences outcomes in carbapenem-resistant Gram-negative infections and found that an MIC of 2 mg/L was associated with significantly higher clinical failure and 28-day mortality in patients receiving colistin monotherapy, while no adverse MIC–outcome association was observed with colistin–meropenem combination therapy, supporting combination therapy over monotherapy for higher-MIC pathogens, particularly in A. baumannii pneumonia.
- [Uhm SA, Alexander BT, Lyden ER, Cortes-Penfield N, Sayles HR, Azimi SF, et al. Practical vs recent clinical trial use of dalbavancin. JAMA Netw Open. 2026 Jun;9(6):e2616508. doi:10.1001/jamanetworkopen.2026.16508](https://ad-id.co/4gkXemK)
  - Summary: This quality-improvement study compared real-world patients receiving dalbavancin for Staphylococcus aureus bacteremia who retrospectively met DOTS trial criteria with participants in the DOTS randomized trial, finding that the clinical cohort was younger and more socially complex, with substantial proportions experiencing housing instability, injection drug use, and variable insurance coverage—characteristics not reported in the trial—highlighting important differences between trial populations and routine clinical practice that may affect generalisability of trial findings.
- [Shah S, Smith J, Smith BJ, Shields RK. Evaluation of combination therapy duration for endocarditis secondary to Enterobacterales. Antimicrob Agents Chemother. 2026 May 28; doi:10.1128/aac.00357-26](https://ad-id.co/4vbdzzb)
  - Summary: This multicenter retrospective study of 73 patients with Enterobacterales endocarditis found that short-course combination antimicrobial therapy (≤14 days) was not associated with increased 90-day mortality or higher rates of treatment discontinuation due to adverse drug reactions compared with longer courses, while being associated with a shorter post-infection hospital stay, supporting the need for an RCT to define the optimal duration of combination therapy in this population.
- [Meena DS, Kumar D, Bohra GK. Combination antibiotic therapy in Staphylococcus aureus endocarditis: evidence, controversies, and future directions. Infection. 2026 May 18; doi:10.1007/s15010-026-02815-w](https://ad-id.co/4vaP5pt)
  - Summary: This narrative review of experimental and clinical evidence on combination antimicrobial therapy for Staphylococcus aureus infective endocarditis concludes that while combination regimens may reduce bacteremia duration in selected cases—particularly emerging daptomycin plus ceftaroline for persistent bacteremia—overall evidence remains largely observational with no consistent survival benefit, increased toxicity observed with adjunctive rifampin or aminoglycosides, and no support for routine combination therapy, thereby favoring a selective, case-based approach pending higher-quality multicenter trials.
- [Martins MF, Govindan R, Almaghlouth NK, Kirby JE, Kentoffio KJ, Farmakiotis D, et al. A fatal case of *Legionella micdadei* prosthetic valve endocarditis diagnosed by plasma microbial cell-free DNA metagenomic sequencing. Lancet Infect Dis. 2026 Jun 8; doi:10.1016/S1473-3099(26)00223-9](https://ad-id.co/4fKUEX5)
  - Summary: This case report describes fatal Legionella micdadei prosthetic valve endocarditis in an immunocompromised patient, where delayed recognition of an initially indolent outpatient course led to rapid deterioration into mixed shock from valve dehiscence and sepsis, with diagnosis ultimately achieved through plasma microbial cell-free DNA metagenomic next-generation sequencing and confirmatory culture, highlighting the limitations of conventional diagnostics in culture-negative endocarditis and proposing earlier use of metagenomic testing to improve timely pathogen identification in high-risk patients.

**Gastrointestinal Tract Infections**

- [Ryan ET, Qadri F, Lynch JA. Global cholera-control efforts — progress and remaining challenges. N Engl J Med. 2026 Jun 6;394:2177-2180. doi:10.1056/NEJMp2600873](https://ad-id.co/4fMKqW3)
  - Summary: This perspective highlights that ending the ongoing cholera pandemic, which began in 1961, will require improved vaccines with better efficacy in young children alongside sustained global investment in access to safe water and adequate sanitation to address the underlying drivers of transmission.
- [GBD 2023 Diarrhoeal Disease and Enteric Infectious Diseases Collaborators. Global burden of enteric infectious diseases, diarrhoeal diseases, and corresponding aetiologies, 1990–2023: a systematic analysis for the Global Burden of Disease Study 2023. Lancet Infect Dis. 2026 Jun 2; doi:10.1016/S1473-3099(26)00194-5](https://ad-id.co/4vTBovo)
  - Summary: This Global Burden of Diseases 2023 analysis estimated that enteric infectious diseases caused approximately 1.27 million deaths worldwide in 2023, showing substantial declines since 1990 but with persistently high mortality concentrated in South Asia and sub-Saharan Africa, particularly among children under five, and identified rotavirus, Shigella, and adenovirus as leading aetiological drivers, while noting that 141 of 204 countries met the GAPPD child mortality target but sustained global efforts are still required to further reduce preventable deaths.
- [Lee YC, Liu JH, Mülder DT, Yang JY, Chiang TH, Hsu WF, et al. Cost-effectiveness of fecal immunochemical testing alone vs co-testing with *Helicobacter pylori* stool antigen. JAMA. 2026 Jun 1; doi:10.1001/jama.2026.6908](https://ad-id.co/4vOJd59)
  - Summary: This economic evaluation based on a pragmatic randomized trial and lifetime Markov modelling found that inviting individuals to undergo combined Helicobacter pylori stool antigen testing alongside fecal immunochemical testing for colorectal cancer screening was generally more cost-effective than FIT alone, being cost-saving in most simulations in Taiwan and remaining cost-effective in other settings, with results strongly driven by underlying H. pylori prevalence and real-world adherence patterns.

**Sexually Transmitted Infections**

- [Visser M, Wolffs PFG, Hoebe CJPA, van Benthem BHB, van Dam AP, Twisk DE, et al. Antimicrobial susceptibility patterns of *Neisseria* *gonorrhoeae* across anatomical sites within and between individuals in the Netherlands: a retrospective observational study. Lancet Microbe. 2026 Jun 3; doi:10.1016/j.lanmic.2026.101381](https://ad-id.co/43wuY9h)
  - Summary: This large retrospective surveillance study of Neisseria gonorrhoeae infections in MSM and women attending Dutch sexual health clinics found that pharyngeal isolates appeared to have higher ceftriaxone MICs and azithromycin resistance at the population level, but within-individual multi-site sampling showed similar susceptibility across anatomical sites with discordant MIC patterns in about 10% of cases, suggesting that apparent pharyngeal predominance of antimicrobial resistance may largely reflect between-person variation rather than true site-specific differences, while also indicating that single-site sampling may miss a meaningful proportion of resistance and that multi-site culture could improve AMR detection and surveillance accuracy.

**CNS Infections**

- [Edwards KM, Rathore MH. Resurgence of tetanus—reasons why the vaccine remains instrumental. JAMA. 2026 Jun 8; doi:10.1001/jama.2026.9347](https://ad-id.co/4vJmnMj)
  - Summary: This brief clinical overview describes tetanus as a severe and potentially fatal disease caused by Clostridium tetani spores, which germinate in anaerobic wound environments and release tetanospasmin toxin that disrupts neurotransmitter release, resulting in sustained muscle contraction and severe, intractable spasms that often require intensive care management.

**Urinary Tract Infections**

- [Razazi K, Blot PL, Renou A, Lombardi Y, Hille H, Devaquet J, et al. Clinical spectrum, outcome and impact of nephrectomy in critically ill adult patients with emphysematous pyelonephritis: the PYELEMPHY observational retrospective multicenter study. Ann Intensive Care. 2026;16:100086. doi:10.1016/j.aicoj.2026.100086](https://ad-id.co/4vUmUvb)
  - Summary: This multicenter retrospective ICU cohort study of 109 patients with emphysematous pyelonephritis found high morbidity and mortality associated with the condition, with septic shock occurring in most cases, and showed that early nephrectomy (within 24 hours of ICU admission) was associated with improved 90-day survival without a significant increase in major adverse kidney events, suggesting a potential survival benefit for early surgical intervention in severe cases alongside antibiotics and drainage.

**Ocular Infections**

- [Tuft S, Taylor EH, McLean K, Thirunavukarasu AJ, Herbert R, Liu S, et al. Regional and temporal trends in antimicrobial susceptibility among isolates from bacterial keratitis: a systematic review and meta-analysis. Lancet Microbe. 2026 Jun 2; doi:10.1016/j.lanmic.2026.101359](https://ad-id.co/4elEXDd)
  - Summary: This systematic review and meta-analysis of 340 studies including over 129,000 culture-positive bacterial keratitis cases from 47 countries found significant regional differences in antimicrobial susceptibility, with lower fluoroquinolone (e.g., ciprofloxacin) susceptibility in South Asia compared with high-income and some other regions for key pathogens such as Staphylococcus spp and Pseudomonas spp, while showing no clear evidence of temporal decline in susceptibility over time, and concluding that standardized reporting is needed to reduce heterogeneity and improve future surveillance and empirical treatment guidance.

**Respiratory Tract Infections**

- [Dona D, Brigadoi G, Silva R, Liberati C, Zin A, Minotti C, et al. Effects of antibiotic treatment on clinical outcomes in patients with COVID-19: a systematic review and meta-analysis. Lancet Microbe. 2026 Jun 10; doi:10.1016/j.lanmic.2026.101358](https://ad-id.co/4vPeuoP)
  - Summary: This systematic review and meta-analysis of 11 randomized controlled trials found that antibiotic use in patients with non-severe COVID-19 does not improve mortality, hospitalisation, or need for invasive mechanical ventilation, while showing no difference in serious adverse events, and similarly provides no clear clinical benefit in severe or critical COVID-19 aside from a possible but low-certainty small mortality reduction, supporting WHO 2025 guidance that antibiotics should be avoided in COVID-19 patients without strong suspicion of bacterial infection to prevent unnecessary antimicrobial use and resistance.
- [van Daalen FV, Boersma WG, van de Garde EWM, van der Mooren FM, Roescher N, Schouten JA, et al. Management of community-acquired pneumonia in adults: practice guideline from the Dutch Working Party on Antibiotic Policy (SWAB) and Dutch Association of Chest Physicians (NVALT). CMI Communications. 2026 May 28; doi:10.1016/j.cmicom.2026.105206](https://ad-id.co/3RYXr5b)
  - Summary: This Dutch multidisciplinary guideline for the diagnosis and antibiotic treatment of community-acquired pneumonia (CAP) in hospitalized adults provides evidence-based recommendations across severity categories, advising against routine sputum and blood cultures in mild to moderately severe cases and against procalcitonin-guided antibiotic initiation, while recommending targeted empirical antibiotic choices ranging from amoxicillin or doxycycline in mild CAP to cephalosporins with fluoroquinolones in ICU patients, a typical five-day treatment duration in patients with clinical improvement, selective use of corticosteroids in severe hypoxia or mechanical ventilation, and avoidance of routine follow-up imaging in uncomplicated recovery.
- [Keske Ş, Prendki V, Fragkou PC, Rello J. A European perspective on antibiotic use in viral respiratory infections: new paradigm integrating pathogens with host factors. Clin Microbiol Infect. 2026 Jun 6; doi:10.1016/j.cmi.2026.06.001](https://ad-id.co/49V0TE0)
  - Summary: Respiratory viruses, including influenza, RSV, and COVID-19, contribute substantially to emergency department visits and hospital admissions—particularly in infants and older adults—and remain a major driver of severe acute respiratory infections in Europe, with ongoing stewardship concerns around frequent but often unconfirmed bacterial coinfection and implications for guideline-based antimicrobial use.
- [Kumar A, Le MPT. Point-of-care ultrasound for the diagnosis of pneumonia. JAMA. 2026 Jun 3; doi:10.1001/jama.2026.4782](https://ad-id.co/49ZCHQT)
  - Summary: Community-acquired pneumonia accounts for a substantial healthcare burden with over 1.4 million emergency department visits and 740,000 hospitalizations annually in the US, and while chest radiography remains the most common diagnostic imaging modality, it has only moderate sensitivity and specificity compared with CT imaging—which is more accurate but less accessible, more costly, and associated with higher radiation exposure—highlighting an ongoing trade-off between diagnostic accuracy and feasibility in pneumonia diagnosis, particularly in resource-limited settings.
- [Nguyen ATK, Paterson DL, Mo Y, Ezure Y, Thanh Duy P, Thwaites CL. Efficacy and safety of newer antibiotics versus generic antibiotics for hospital-acquired and ventilator-associated bacterial pneumonia: a systematic review and meta-analysis of randomized controlled trials. Clin Infect Dis. 2026 Jun 2;82(Suppl 5):S116-S125. doi:10.1093/cid/ciag045](https://ad-id.co/4atbxlu)
  - Summary: In a systematic review and meta-analysis of 8 RCTs including 2881 patients with hospital-acquired and ventilator-associated bacterial pneumonia, newer antibiotics showed no significant difference compared with generic antibiotics in 28-day mortality, clinical response, or overall microbiological response, but were associated with significantly lower nephrotoxicity than colistin-based regimens and improved microbiological eradication in carbapenem-resistant Gram-negative infections, suggesting similar overall efficacy but potential advantages in toxicity and targeted resistant pathogens.
- [Potter-Schwartz L, Swe MM, Sharland M, Bielicki JA, Cooper BS, Lim C. Effectiveness of amoxicillin and amoxicillin-clavulanate for the treatment of community-acquired pneumonia in adults and children: a systematic review and meta-analysis. BMJ Open. 2026 Jun; doi:10.1136/bmjopen-2025-112219](https://ad-id.co/4vdIivt)
  - Summary: In a systematic review and meta-analysis of 44 studies involving 45,400 patients, amoxicillin and amoxicillin-clavulanate showed no clear difference in clinical resolution or clinical failure compared with each other or other antibiotics for community-acquired pneumonia in adults and children, with results limited by heterogeneity and uncertainty across trials, indicating that current evidence does not support clear superiority of either regimen in CAP treatment.

**Infections in Children**

- [de Sousa Orlandin EA, Oharomari LK, Gonçalves-Ferri WA. The management of neonatal sepsis and septic shock. Curr Treat Options Pediatr. 2026 Jun 3;12:19. doi:10.1007/s40746-026-00379-3](https://ad-id.co/4vNfmu3)
  - Summary: This review of neonatal sepsis and neonatal septic shock highlights the evolving shift from protocol-based management toward individualized, multimodal care integrating clinical assessment with biomarkers, point-of-care ultrasound, echocardiography, and emerging machine learning tools, while noting that although these diagnostic and monitoring approaches may improve early recognition and treatment, most biomarkers and adjunctive therapies remain limited or investigational, and future advances will depend on robust validation, implementation studies, and context-adapted integration into neonatal care pathways.

**Mycobacterial Infections**

- [Gray RM, Wasserman S, Wilkinson RJ. An update on recent developments in the drug treatment of tuberculous meningitis. Expert Rev Anti Infect Ther. 2026 Jun 11; doi:10.1080/14656566.2026.2686440](https://ad-id.co/4ew8evg)
  - Summary: Tuberculous meningitis remains a highly lethal and disabling form of tuberculosis treated with a longstanding standard regimen of four antitubercular drugs plus corticosteroids, and although recent trials and pharmacokinetic studies have explored treatment intensification, no clear clinical benefit has been demonstrated so far, suggesting that future improvements in outcomes may depend less on increasing antimycobacterial activity and more on targeted modulation of the host neuroinflammatory response.
- [Siddiqi OK, Jahn B, Rochau U, Walukaga S, Rajasingham R, Siebert U. Cost-effectiveness analysis of Xpert MTB/RIF Ultra for the diagnosis of HIV-associated tuberculous meningitis. Clin Infect Dis. 2026 Jun 9; doi:10.1093/cid/ciag348](https://ad-id.co/3QoysYq)
  - Summary: In a decision-analytic cost-effectiveness study of HIV-associated tuberculous meningitis in Zambia, incorporation of CSF Xpert MTB/RIF Ultra alongside standard clinical assessment was found to be highly cost-effective at $242.76 per QALY gained, with an estimated number needed to test of 125 to prevent one TBM death, and results were robust across sensitivity analyses, supporting WHO recommendations for its use in high-burden, resource-limited settings where empiric treatment is often required due to limited diagnostic sensitivity.
- [Warp PV, Kim M, Williams K, Miner A, Maderal AD. Risk of immunologic reactions during monthly therapy for Hansen disease. JAMA Dermatol. 2026 Jun 3; doi:10.1001/jamadermatol.2026.1262](https://ad-id.co/4veKt1M)
  - Summary: Leprosy (Hansen disease) is a chronic granulomatous infection caused by the Mycobacterium leprae complex that primarily affects skin and peripheral nerves, with clinical manifestations determined by host cellular immune response, and major morbidity and disability arising from immunologically driven reactions—particularly type 1 (reversal) reactions and erythema nodosum leprosum—which can lead to neuritis and progressive nerve damage.
- [Ma Z, Yu X, Yu Y, Wang Y, Shi J, Jing W, et al. Near-point-of-care diagnosis of tuberculosis with LyocartE MTB assay: a multicentre diagnostic accuracy study. Clin Infect Dis. 2026 May 29; doi:10.1093/cid/ciag342](https://ad-id.co/4vJEm5f)
  - Summary: In a multicentre prospective diagnostic accuracy study of 689 individuals with presumptive tuberculosis in China, the LyocartE near point-of-care assay demonstrated high sensitivity and specificity for TB detection using both sputum (95.2% sensitivity, 98.6% specificity versus microbiological reference standard) and tongue swab specimens (up to 87.4% sensitivity and 99.7% specificity versus Xpert Ultra), with strong correlation to Xpert Ultra cycle threshold values, suggesting it is a rapid and accurate non-sputum diagnostic tool with potential to expand TB detection pending further validation in paucibacillary and asymptomatic populations.
- [Goletti D, Aiello A, Chandran S, Martineau AR. Diagnosing tuberculosis infection: from the host to the pathogen. Cold Spring Harb Perspect Med. 2026 Jan 20; doi:10.1101/cshperspect.a041955](https://ad-id.co/4en04oy)
  - Summary: Tuberculosis infection (TBI) affects approximately one-quarter of the global population and carries a lifelong 5–10% risk of progression to active TB disease, with current WHO-endorsed diagnostics relying on immune-based tests such as interferon-γ release assays and tuberculin skin testing, while emerging research focuses on developing assays that directly detect circulating Mycobacterium tuberculosis components (including DNA, proteins, peptides, lipoglycans, and lipoproteins) to overcome limitations of immunodiagnostic approaches, although these novel methods remain experimental and not yet clinically validated.
- [Alipoor SD, Guthrie J, Davies Forsman L, Di Nardo AR, Brighenti S. Immune endotypes in tuberculosis: keys to decoding disease complexity. J Intern Med. 2026 Apr 3; doi:10.1111/joim.70092](https://ad-id.co/4vNG7hS)
  - Summary: Tuberculosis remains a major global health challenge treated with standard multidrug regimens that effectively kill Mycobacterium tuberculosis but fail to address persistent inflammation, lung damage, or immune dysregulation contributing to poor outcomes and recurrence, and this review proposes immune endotyping as a precision medicine approach to stratify TB into biologically distinct immune-driven subtypes using multi-omics and computational profiling to better guide host-directed therapies targeting pathways such as hyperinflammation, immunosuppression, coagulopathy, and metabolic dysfunction, with the goal of enabling more individualized and effective treatment strategies beyond traditional one-size-fits-all regimens.
- [Suresh J, Kumar R, Hissar S, Shukla S, Vivekanandan K. Ocular toxicity associated with ethambutol in the South-East Asia Region. Int Ophthalmol. 2026 Jun 4;46:251. doi:10.1007/s10792-026-04131-6](https://ad-id.co/4a1sm73)
  - Summary: Post-market pharmacovigilance data from VigiBase (to Jan 2026) identified 49,327 ethambutol-suspected adverse events globally, including 4,748 eye disorder cases and 220 blindness cases—predominantly in South-East Asia (notably India)—raising concern that dose-frequency changes to daily ethambutol may be associated with increased optic neuropathy and blindness risk, and highlighting the need for routine ophthalmic monitoring and safer TB drug alternatives.
- [Wang X, Wang Y, Yan G, Chu N, Huang H, Nie W, et al. The clinical diagnostic value of metagenomic next-generation sequencing for patients with suspected nontuberculous mycobacterial osteoarticular infection: a national multicentre clinical cohort. Int J Infect Dis. 2026 Jun;167:108578. doi:10.1016/j.ijid.2026.108578](https://ad-id.co/4avWLdG)
  - Summary: In a multicentre diagnostic accuracy study of 193 patients with suspected nontuberculous mycobacterial osteoarticular infection (NTM OAI), metagenomic next-generation sequencing (mNGS) demonstrated near-perfect diagnostic performance compared with MGIT culture (sensitivity 100% vs 7.7%, specificity 99.4% vs 80.2%, AUC 0.997), indicating substantially superior ability to detect low-burden infections and supporting mNGS as a highly accurate diagnostic tool for NTM OAI.
- [Martín C, Gonzalo-Asensio J, Aguiló N, Arbués A. Toward tuberculosis elimination: an update on tuberculosis vaccines in clinical trials. Int J Infect Dis. 2026 Jun;167:108513. doi:10.1016/j.ijid.2026.108513](https://ad-id.co/4vdBwpu)
  - Summary: Tuberculosis elimination will not be achievable without a vaccine that prevents pulmonary disease and transmission, as BCG provides strong protection against severe childhood TB but limited and inconsistent protection against adult pulmonary disease, and although 16 vaccine candidates (including 4 in Phase 3 trials) are in development, progress is constrained by the absence of validated correlates of protection, challenges in trial design, and the need to align prevention-of-disease, infection, and recurrence endpoints with evolving understanding of TB infection biology.
- [Nyang’wa BT, Motta I, Moodliar R, Solodovnikova V, Rajaram S, Rasool M, et al. Pharmacokinetics, bactericidal activity and toxicity of short oral regimens for rifampicin-resistant tuberculosis treatment. Nat Commun. 2026; doi:10.1038/s41467-026-74335-y](https://ad-id.co/4eMjDar)
  - Summary: In the PRACTECAL-PKPD pharmacokinetic–pharmacodynamic study of rifampicin-resistant tuberculosis in Belarus and South Africa, exposures to bedaquiline, pretomanid, linezolid, moxifloxacin, and clofazimine in BPaL-based regimens did not correlate with sputum bacterial clearance speed, although BPaLM showed a 20% increased bacillary killing rate compared with standard of care while BPaL and BPaLC showed reduced killing rates; severe disease was independently associated with slower bacterial clearance, and linezolid exposure was higher in patients with anaemia or neutropenia, overall supporting WHO recommendations favouring BPaLM as the preferred regimen balancing efficacy and safety.
- [Araújo-Pereira M, Andrade BB. Toward tuberculosis elimination strategy: the role of IGRA on immunocompetent individuals. Int J Infect Dis. 2026 Jun;167:108518. doi:10.1016/j.ijid.2026.108518](https://ad-id.co/4ayiwtn)
  - Summary: This narrative review argues that interferon-gamma release assays (IGRAs) in tuberculosis infection should be interpreted as reflecting dynamic and quantitative immune states rather than fixed infection categories, highlighting that immunocompetent individuals exhibit multiple IGRA-defined trajectories (including conversion and reversion near assay cut-offs), and proposing that longitudinal and quantitative IGRA interpretation integrated with exposure and host risk factors could improve risk stratification, prioritization of preventive therapy, and support tuberculosis elimination strategies under the WHO End TB framework.
- [Esmail H, Thienemann F, Sossen B, Mukasa SL, Lakay F, Munro JE, et al. PET–CT benchmarked detection and 5-year progression of asymptomatic tuberculosis: a longitudinal, prospective cohort study. Lancet Respir Med. 2026;14:505-520. doi:10.1016/S2213-2600(26)00056-1](https://ad-id.co/4gfvnV2)
  - Summary: In a prospective cohort of 250 asymptomatic HIV-negative high-risk tuberculosis contacts in South Africa followed for up to 5 years, baseline [18F]-FDG PET–CT identified radiographically evident tuberculosis lesions that strongly predicted future disease (HR 28.54 vs normal lungs), with most subsequent bacteriologically confirmed cases already showing PET–CT abnormalities despite being asymptomatic at diagnosis, while chest X-ray computer-aided detection systems demonstrated good diagnostic performance (AUC 0.86–0.89), supporting PET–CT as a sensitive reference standard and suggesting that subclinical radiographic disease is common prior to clinical tuberculosis onset.
- [Eick KL, Gan M, Thompson S, Dial CN, Deal M, DiBiase LM, et al. Hospital environment–associated sources of Mycobacterium abscessus infection in transplant recipients. JAMA Netw Open. 2026 Jun 11;9(6):e2617898. doi:10.1001/jamanetworkopen.2026.17898](https://ad-id.co/4vSQDEQ)
  - Summary: In a whole-genome sequencing cohort study of 24 transplant patients with Mycobacterium abscessus infection and 54 environmental isolates in a tertiary hospital, patient strains clustered genetically with organisms recovered specifically from hospital tap water but not other environmental sources, indicating persistent, unit-specific water system reservoirs as the primary source of healthcare-associated transmission rather than person-to-person spread, with most infections linked to a dominant circulating clone (DCC7), underscoring the importance of water system control measures to prevent infection in vulnerable patients.
  - **Editorial Commentary:**
    [Hopman J, Meda M. Mycobacterium abscessus and the built environment—A preventable source of infections. JAMA Netw Open. 2026 Jun 11;9(6):e2617907. doi:10.1001/jamanetworkopen.2026.17907](https://ad-id.co/43wJuhg)
- [da Silva KE, dos Santos PCP, Tsuha DH, Walter KS, Cunha EAT, Colijn C, et al. Comparison of phylogenetic metrics of transmission between symptomatic and asymptomatic tuberculosis in individuals who were incarcerated in Brazil in 2008–24: a retrospective genomic epidemiology study. Lancet Microbe. 2026 Jun 11; doi:10.1016/j.lanmic.2026.101407](https://ad-id.co/4ej7OYN)
  - Summary: In a retrospective genomic epidemiology study of 2362 Mycobacterium tuberculosis lineage 4 cases in Mato Grosso do Sul, Brazil, including active case finding in prisons, asymptomatic tuberculosis cases (45.1%) showed no significant differences from symptomatic cases in genomic clustering, transmission fitness metrics, or inferred secondary transmission, indicating that asymptomatic individuals contribute substantially to ongoing transmission in high-burden settings and that symptom-based screening alone is insufficient to interrupt transmission, supporting the need for expanded active screening strategies in high-risk populations.

**Fungal Infections and Antifungal Agents**

- [Smith AGC, Valentine A, D’Angeli MD, Brezak A, Spence Davizon E, Makarewicz VA, et al. Enhanced detection of *Candidozyma auris* colonization with universal screening on admission. Clin Infect Dis. 2026 Jun 9; doi:10.1093/cid/ciag351](https://ad-id.co/4vcFEGk)
  - Summary: In a universal admission screening study of 22,742 patients, Candidozyma auris colonization was detected in 0.1% (25 cases), with 80% of colonized patients not meeting existing public health risk-based screening criteria, suggesting that current targeted screening approaches may miss a substantial proportion of colonized individuals and that broader screening strategies may be needed, although findings may not be generalizable to higher-incidence settings.
- [Seeßle J, Pelivan E, Kirchner M, Boxberger M, Michl P, Merle U. Clinical characteristics and outcomes of invasive fungal infections in critically ill patients with liver cirrhosis. BMC Infect Dis. 2026 Jun 4;26:1088. doi:10.1186/s12879-026-13709-5](https://ad-id.co/4ettPEv)
  - Summary: In a retrospective single-center cohort of 488 ICU patients with liver cirrhosis, invasive fungal infections occurred in 6.1%—mainly invasive candidiasis and pulmonary aspergillosis with onset around 2–3 weeks after admission—and were associated with more severe liver disease, higher need for invasive supportive therapies, and significantly reduced 30- and 90-day survival, particularly in patients with acute-on-chronic liver failure, highlighting the importance of early surveillance for invasive fungal infections in high-risk cirrhotic ICU patients.
- [Hemmings SJ, Varaden D, Barnes J, Elmi MS, Skillern A, Barratt B, et al. Diversity analysis of indoor and outdoor fungal bioaerosols in UK households: a prospective, observational, longitudinal study. Lancet Microbe. 2026 Jun 4; doi:10.1016/j.lanmic.2026.101379](https://ad-id.co/4vM7HMt)
  - Summary: In the West London Healthy Home and Environment Study of 118 households (262 air samples), indoor fungal bioaerosols were found to be significantly more diverse than outdoor air (higher richness and Shannon diversity), with distinct community composition dominated by Penicillium, Aspergillus, and Wallemia, stronger seasonal variation indoors, and higher fungal burdens in homes with visible mould, supporting the conclusion that indoor fungal exposure in urban UK homes is highly dynamic and potentially relevant to respiratory health risks, particularly in vulnerable individuals such as those with asthma or allergic sensitisation.
- [Gibert C, Bigot J, Brissot E, Banet A, Millet N, Henequin C, et al. Clinical and analytical evaluation of a commercial *Aspergillus fumigatus* real-time PCR assay for the diagnosis of invasive pulmonary aspergillosis in patients with hematological malignancies. J Infect Dis. 2026 May 31; doi:10.1093/infdis/jiag280](https://ad-id.co/4fFYErM)
  - Summary: In a retrospective study of 153 patients with hematological malignancies evaluated for invasive pulmonary aspergillosis, Aspergillus fumigatus qPCR showed variable diagnostic performance depending on specimen type and positivity thresholds (sensitivity 0.73 and specificity 0.71 with a single positive test, versus higher specificity but lower sensitivity with stricter criteria), with evidence of cross-reactivity with non-fumigatus Aspergillus and other molds, leading to reclassification and antifungal treatment in some cases, highlighting that qPCR results should be interpreted cautiously alongside clinical and other mycological evidence.
- [Li LX, Xue J, Kates OS, Avery RK, Zhang SX, Baddley JW, et al. Demographics and geographic accessibility of invasive fungal infection clinical trial sites. JAMA Netw Open. 2026 Jun 11;9(6):e2617927. doi:10.1001/jamanetworkopen.2026.17927](https://ad-id.co/4veHYN0)
  - Summary: In a US cross-sectional analysis of 27 systemic antifungal clinical trials (249 sites) registered from 2015–2025, trial locations were unevenly distributed geographically with marked overrepresentation in certain regions (notably the South Atlantic division) and substantial within-region variability, suggesting that access to antifungal clinical trials is geographically heterogeneous and may contribute to inequities in participation, particularly for socioeconomically disadvantaged and rural populations, although further demographic analysis was ongoing beyond the reported results.

**Diagnostics**

- [Thaden JT, Ruffin F, Park LP, Farahani P, Goel D, Parsons JB, et al. The impact of follow-up blood cultures on mortality and management in patients with gram-negative bloodstream infections: a validation cohort study. BMC Infect Dis. 2026 May 28; doi:10.1186/s12879-026-13579-x](https://ad-id.co/43D2dI1)
  - Summary: In adults with gram-negative bloodstream infections, obtaining follow-up blood cultures was associated with reduced attributable in-hospital mortality even after adjustment for immortal time bias, while persistent bacteremia was linked to increased mortality, with potential benefit mediated through increased infectious diseases consultation and greater likelihood of source control interventions. The pending results from the BALANCE+ trial should give further information on this question.
- [Britt NS, Khader K, He T, Prinzi AM, Hommel B, Timbrook TT, et al. Clinical impact of the BIOFIRE® FILMARRAY® Pneumonia Panel in the Veterans Affairs Healthcare System: a difference-in-differences study. Clin Infect Dis. 2026 Jun 12; doi:10.1093/cid/ciag328](https://ad-id.co/4eoVvKy)
  - Summary: In a multicentre retrospective study of ICU patients with pneumonia in Veterans Health Administration hospitals, implementation of the BIOFIRE® FILMARRAY® Pneumonia Panel was associated with improved antibiotic optimization, including increased early antimicrobial de-escalation and appropriate therapy—particularly in culture-positive cases—with signals of reduced ICU mortality, especially in hospitals with stronger antimicrobial stewardship capacity, although no overall difference in 30-day mortality was observed.
  - **Editorial Commentary:**
    [Klompas M, Rhee C. Test first, treat later: could shifting the pneumonia treatment paradigm help us better realize the potential benefits of rapid molecular panels? Clin Infect Dis. 2026 Jun 12; doi:10.1093/cid/ciag330](https://ad-id.co/4viDOUs)

**Improving Clinical Research**

- [Hardy MJ, Harris PNA, Mo Y, Venkatesh B, Hammond NE, Peel TN, et al. Antibiotic clinical trials: a review of the past, present challenges, and potential solutions. Clin Infect Dis. 2026 Jun 2;82(Suppl 5):S126-S137. doi:10.1093/cid/ciag017](https://ad-id.co/3QHwkLt)
  - Summary: A targeted review of 193 antibiotic randomized controlled trials published between 2013 and 2022 found that most were traditional multicentre, two-arm parallel designs predominantly conducted in high-income settings, with limited representation from low- and middle-income countries despite high antimicrobial resistance burdens, and the authors highlight the need for more innovative trial designs such as adaptive, platform, and pathogen-directed approaches to improve efficiency, global relevance, and equity in future antibiotic research.
- [David S, Leone M, Girardis M, Müller MM, Gavrilovic S, Domizi R, et al. Innovation in sepsis trials: rethinking endpoints, statistics and patient stratification. Intensive Care Med Exp. 2026 Jun 1;14:65. doi:10.1186/s40635-026-00911-7](https://ad-id.co/4et0dqJ)
  - Summary: Despite repeated failures of traditional randomized controlled trials to identify effective causal therapies for sepsis, emerging precision medicine–oriented trials such as ANDROMEDA-SHOCK-2, IMMUNOSEP, INSPIRE, and TIGRIS suggest that patient stratification based on physiological or immunological phenotypes, combined with adaptive designs and alternative endpoints beyond mortality, may uncover clinically meaningful treatment effects, indicating a shift toward more biologically informed and targeted approaches in sepsis research.
- [Paterson DL, Melnick D, Dane A, Goldberger M, Schramm M, Huang SS, et al. Considerations in designing clinical trials of novel antibacterial agents using a superiority design. Clin Infect Dis. 2026 Jun 2;82(Suppl 5):S138-S146. doi:10.1093/cid/ciag207](https://ad-id.co/4vlsXZJ)
  - Summary: Regulatory approval trials for new antibacterial agents are most commonly designed as noninferiority studies and are recommended as the default approach in antibiotic development; however, superiority trial designs may be appropriate for nontraditional agents used as add-on therapies, and careful consideration is required regarding optimal study populations, endpoints, trial structure, and regulatory engagement to ensure appropriate evaluation of clinical benefit.
- [Shou Y, Ezure Y, Kang MP, Yeo JE, Muthiah VK, Tan YQD, et al. Consent modes and consent rates in randomized controlled trials for severe infections: a scoping review. Clin Infect Dis. 2026 Jun 2;82(Suppl 5):S147-S153. doi:10.1093/cid/ciaf715](https://ad-id.co/4aNBuw6)
  - Summary: A scoping review of 112 randomized controlled trials of antibiotics in critically ill patients with severe infections found that most required pre-randomization written consent with surrogate consent commonly allowed, but reporting of consent processes and failure rates was inconsistent and incomplete; where reported, consent failure rates varied widely (median 13%, up to 66%), highlighting substantial heterogeneity in recruitment loss and the need for standardized reporting to better understand and address consent-related barriers in acute infection trials.
- [Mafiz T, Rieg S, Wolkewitz M. How to evaluate the robustness of causal findings in observational studies: application of E-values and bounding factors. Clin Microbiol Infect. 2026 Jun 8; doi:10.1016/j.cmi.2026.05.048](https://ad-id.co/4vaqJw9)
  - Summary: This methodological review describes the use of E-values and bounding factors as complementary sensitivity analysis tools for assessing the robustness of causal inferences from observational studies to unmeasured confounding, highlighting their ease of application, limitations, and role in improving transparency and interpretation within frameworks such as GRADE and TARGET.

**Company Announcements**

- [Eli Lilly and Company. Lilly announces three acquisitions to build infectious disease portfolio. 2026 May 26. https://investor.lilly.com/news-releases/news-release-details/lilly-announces-three-acquisitions-build-infectious-disease](https://ad-id.co/4eeBQwI)
  - Summary: Eli Lilly and Company announced agreements to acquire Curevo Inc., LimmaTech Biologics AG, and a third vaccine-focused company for up to approximately $3.8 billion in total combined milestone-based payments, expanding its infectious disease pipeline to include vaccines targeting viral pathogens such as shingles and Epstein–Barr virus (linked to long-term neurological and oncological outcomes) and bacterial pathogens including Staphylococcus aureus, Neisseria gonorrhoeae, and Chlamydia trachomatis, with the strategic goal of shifting from treatment to prevention in response to rising antimicrobial resistance and growing evidence of long-term sequelae following common infections.
- [Omnix Medical. Omnix Medical announces first patients dosed in Phase II trial of antimicrobial lead compound OMN6. 2026 Jun 4. https://www.biospectrumasia.com/news/26/27744/omnix-medical-announces-first-patients-dosed-in-phase-ii-trial-of-antimicrobial-lead-compound-omn6.html](https://ad-id.co/4vNblWr)
  - Summary: Omnix Medical has initiated dosing in a Phase II randomized, double-blind, placebo-controlled, dose-ranging trial of its first-in-class antimicrobial peptide OMN6 for hospital-acquired and ventilator-associated pneumonia caused by carbapenem-resistant Acinetobacter baumannii, a WHO critical priority pathogen, aiming to evaluate safety, tolerability, and pharmacokinetics of a novel membrane-disrupting mechanism designed to rapidly destroy multidrug-resistant Gram-negative bacteria with high unmet clinical need and limited therapeutic options.

**General Interest**

- [Paterson DL. Advancing clinical evidence in infectious diseases: an infectious diseases clinical trials network for Asia, the “antimicrobial resistance hot zone”. Clin Infect Dis. 2026 Jun 2;82(Suppl 5):S101-S104. doi:10.1093/cid/ciag122](https://ad-id.co/4ep30B8)
  - Summary: The ADVANCE-ID clinical trials network, comprising over 100 hospitals across Asia with expansion planned into Africa, the Middle East, and Latin America, has enrolled more than 12,000 patients since 2022 and aims to generate high-quality randomized controlled trial evidence to improve treatment, diagnostics, and prevention of serious infections caused by antimicrobial-resistant bacteria, while also promoting equitable access to proven antibiotics and diagnostics in low- and middle-income countries.
- [Aligui AF, Winter Hor S, Blanco-Arévalo A, Gupta A, Saravanan R, Leong J, et al. Challenges to accessing newer antibiotics for multidrug-resistant bacterial infections in Asia: a literature review of multi-stakeholder perspectives. Clin Infect Dis. 2026 Jun 2;82(Suppl 5):S154-S161. doi:10.1093/cid/ciag024](https://ad-id.co/4fLPcTI)
  - Summary: A literature review of 134 publications (2010–2024) examining access to FDA-approved newer antibiotics in Asia identified major barriers across multiple stakeholders, including fragmented epidemiological data, limited health economic evaluations, manufacturing and regulatory constraints, inefficient distribution systems, poor affordability in low-resource settings, and weak governance structures, highlighting the need for coordinated, context-specific strategies to improve equitable antibiotic access in the region.

1. The article summaries in this supplement were generated with assistance from ChatGPT (OpenAI) and reviewed and edited by the authors for accuracy and content.
